# Efficient catenane synthesis by cucurbit[6]uril-mediated azide–alkyne cycloaddition

**DOI:** 10.3762/bjoc.14.158

**Published:** 2018-07-20

**Authors:** Antony Wing Hung Ng, Chi-Chung Yee, Kai Wang, Ho Yu Au-Yeung

**Affiliations:** 1 Department of Chemistry, The University of Hong Kong, Pokfulam Road, Hong Kong, P. R. China

**Keywords:** azide–alkyne cycloaddition, catenane, click chemistry, cucurbit[6]uril, mechanical bond

## Abstract

We report here the efficient synthesis of a series of [3]catenanes featuring the use of cucurbit[6]uril to simultaneously mediate the mechanical and covalent bond formations. By coupling the mechanical interlocking with covalent macrocyclization, formation of topological isomers is eliminated and the [3]catenanes are formed exclusively in good yields. The efficient access to these [3]catenanes and the presence of other recognition units render them promising building blocks for the construction of other high-order interlocked structures.

## Introduction

Catenanes are topologically non-trivial molecules possessing mechanically interlocked macrocycles. The flexible but strong mechanical bond between the interlocked macrocycles offers a unique opportunity for exploiting catenanes as molecular machines or new materials with unusual mechanical properties [1–9]. Over the years, different templates and ring-closing reactions to form the interlocked macrocycles have been developed, but the majority of the reported catenanes is limited to the Hopf link (i.e., the simplest [2]catenane with two crossings) [9]. Synthesis of other high-order catenanes with more interlocked macrocycles and/or more crossing points remain challenging and more general synthetic strategies to catenanes are yet to be realized [10–12]. A major challenge in [*n*]catenane synthesis is the precise spatiotemporal control of the covalent formation of the macrocycles and their mechanical interlocking. Otherwise, various topological isomers such as the non-interlocked products or lower-order catenanes will form, which are detrimental to the synthesis efficiency as well as complicating the purification process [13–19].

One strategy to overcome this challenge is to couple the mechanical bond and covalent bond formation in a single step. If the building block preorganization by reversible interactions (which will lead to mechanical interlocking) is also necessary for the covalent bond formation, the covalent trapping of the non-interlocked precursors will be suppressed and the mechanical interlocking of the macrocycles will be ensured. One example is the use of an active metal template, in which the macrocyclization is mediated by the metal ion inside the cavity of a metal-coordinating macrocycle [20]. Cucurbit[6]uril (CB[6]) has also been demonstrated to bind to ammonium-containing azides and alkynes and to mediate their cycloaddition inside the CB[6] cavity [21–22]. Yet, these strategies have been largely limited to the synthesis of rotaxane-based interlocked systems [23], probably because of the additional challenges associated with the macrocyclization in catenane synthesis. Previously, we have reported a preliminary study of a [6]catenane synthesis featuring the CB[6]-mediated azide–alkyne cycloaddition (CBAAC) using phenanthroline-based building blocks [24]. To further explore the applicability and generality of the CBAAC in the construction of mechanically interlocked molecules, we report here the efficient synthesis of a series of [3]catenanes from a combination of different azide and alkyne building blocks. These [3]catenanes were obtained in good yields (>85%) with straightforward purification procedures. The good compatibility of CBAAC with these various building blocks and the good yields of the [3]catenanes can serve as an entry point to other high-order [*n*]catenanes by introducing additional macrocycles through other non-covalent interactions.

## Results and Discussion

### Building block design

**Scheme 1:**
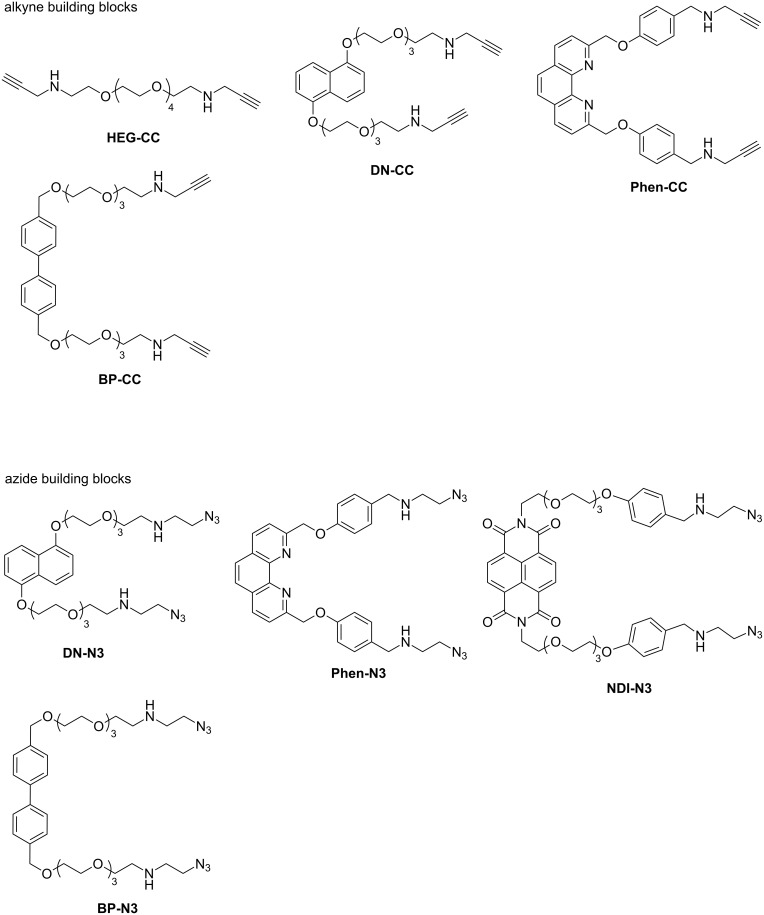
Diazide and dialkyne building blocks used in this study.

The bis(aminoalkyne) and bis(aminoazide) building blocks used in this study are summarized in Scheme 1. The secondary amines are designed to form ammoniums that will strongly bind to CB[6] through ion-dipole interactions under aqueous acidic conditions. Upon formation of the inclusion complex with CB[6], the azide and alkyne functionalities inside the CB[6] cavity will be placed in close proximity and their cycloaddition will be facilitated. As the cycloaddition is preceded by CB[6] binding, triazole formation will therefore ensure the interlocking of the CB[6]. A [1 + 1] macrocyclization of the diazide and dialkyne will then result in the exclusive formation of the [3]catenane with no other topological isomers.

The building blocks contain either a central hexaethylene glycol (HEG) unit or are derived from 1,5-dioxynaphthalene (DN), naphthalenediimide (NDI), 2,9-phenanthroline (Phen) or 4,4’-biphenyl (BP) cores which can engage in additional non-covalent interactions, such as metal–ligand coordination, charge transfer, π-stacking and hydrophobic interactions, for the later interlocking of additional macrocycles to give higher-order [*n*]catenanes. The new building blocks were synthesized following similar procedures as previously described [24].

### Catenane synthesis by CBAAC

#### Synthesis of [3]catenanes

**Scheme 2:**
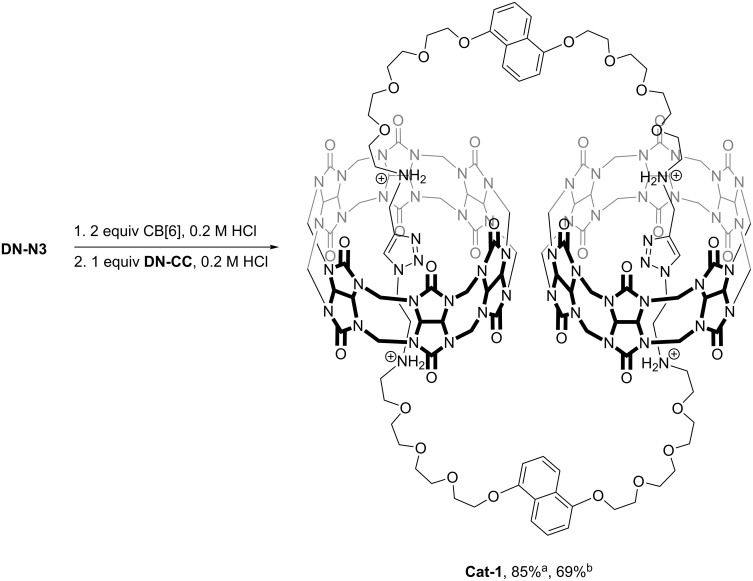
Synthesis of **Cat-1** by CBAAC. ^a^Assembly yield by HPLC; ^b^isolated yield as PF_6_^−^ salt.

The CBAAC-mediated [3]catenane synthesis was first investigated using the dioxynaphthalene building blocks **DN-N3** and **DN-CC**. Different from the previously reported [3]catenane **Cat-0** which is derived from **DN-N3** and **Phen-CC** [24], both **DN-N3** and **DN-CC** contain the flexible ethylene glycol linkers between the terminal azide and alkyne groups, so that any other possible preorganization effects, except that of the ammonium binding to CB[6] could be eliminated to study the efficiency of CBAAC in the catenane formation. In our first trial, a 1:1:2 mixture of **DN-N3**, **DN-CC** and CB[6] in 0.2 M HCl was heated at 70 °C and the reaction mixture was analyzed by LC–MS. After heating for 1 hour, although a peak corresponding to the [1 + 1] cyclized product with *m/z* values consistent with the [3]catenane **Cat-1** could be observed, some unreacted building blocks and unidentified products were also found. Further extending the reaction time to 3, 5 or 8 hours resulted in similar chromatograms with no significant improvement of the yield of **Cat-1**. A similar observation has been reported in a CBAAC-mediated rotaxane synthesis and additional templates have been used to positively cooperate with the CB[6] binding to the ammoniums on the building blocks to improve the efficiency of CBAAC [23]. In view of the low solubility of CB[6] and the possibility of thermal-assisted azide–alkyne cycloaddition of the unbound building blocks that may lead to the incomplete cycloaddition and other side products, we decided to first assemble a pseudo[3]rotaxane CB[6] complex with either the azide or alkyne building block, before introducing the other building block to the reaction mixture. By heating a solution of **DN-N3** in the presence of two equivalents of CB[6] in 0.2 M aq HCl for 2 hours, a clear solution was formed that indicated CB[6] dissolution and formation of the inclusion complex. One equivalent of **DN-CC** was added and the mixture was further heated at 70 °C. LC–MS analysis of the reaction mixture showed that the building blocks were completely consumed after one hour of heating, and that the crude mixture contained the [3]catenane **Cat-1** as the major product with >85% yield (Scheme 2). Using **DN-CC** to first form the pseudo[3]rotaxane CB[6] complex did not affect the efficiency of CBAAC and **Cat-1** was obtained in a similar yield. These results show that the initial formation of the pseudorotaxane complex helps the CBAAC by avoiding any cycloadditions outside of the CB[6] cavity and that CBAAC can be an efficient strategy for catenane synthesis.

**Cat-1** and its interlocked nature were further characterized by MS^2^, ^1^H and ^13^C NMR spectroscopy. The PF_6_^−^ salt of **Cat-1** was isolated in 69% yield as a white solid by precipitation with a saturated NH_4_PF_6_ solution and simple filtration. The ESIMS spectrum showed two peaks at *m/z* = 808.5 and 1076.3, consistent with the 3+ and 4+ ions of **Cat-1**. Similar to **Cat-0**, the MS^2^ spectrum of **Cat-1** revealed CB[6]-bound fragments, showing the strong CB[6]–ammonium interactions are preserved under the MS conditions. Inclusion of the triazole in the CB[6] cavity was evidenced by the upfield shifted triazole resonance from 8.5 ppm to 6.4 ppm when compared with that of free triazole in non-interlocked system [22,24–25]. NOE cross peaks between the triazole and CB[6] protons were also observed. The ^1^H NMR spectrum of **Cat-1** showed one set of aromatic resonances, suggesting the chemical environments of two dioxynaphthalene units are highly similar and that **Cat-1** is adopting a symmetrical conformation in solution, consistent with the tight binding of the diammonium to the CB[6] and the overall flexibility of the [3]catenane structure.

With the success of CBAAC in the efficient synthesis of **Cat-1**, nine other [3]catenanes (**Cat-2**–**Cat-10**) were synthesized from different combinations of the alkyne and azide building blocks under similar conditions (Scheme 3). In all cases, LC–MS analysis of the crude reaction mixtures showed the complete consumption of the building blocks with the [3]catenane as the only major product with yields >85%. This demonstrates not only the high efficiency of CBAAC in catenane synthesis, but also the good compatibility of CBAAC with various types of building blocks. Because of the high yields of these [3]catenanes, their isolations were all straightforward. **Cat-2**–**Cat-10** were isolated either by precipitation as the PF_6_^−^ salts or by preparative HPLC, and were fully characterized by MS, NMR and UV–vis spectroscopy. Similar to **Cat-1**, the upfield shifted triazole ^1^H NMR resonances of **Cat-2**–**Cat-10** at ca. 6.4 ppm are consistent with the inclusion of the triazole in the CB[6] cavity [22,24–25]. The ESIMS, HRMS and MS^2^ spectra are all consistent with the [3]catenanes with a similar fragmentation behavior as that of **Cat-1**. The ^1^H NMR, ^13^C NMR and ESIMS spectra of **Cat-2**–**Cat-10** are shown in Figure 1 and Figure 2, and in Supporting Information File 1, Figures S29–S55 and S60–S68.

**Scheme 3:**
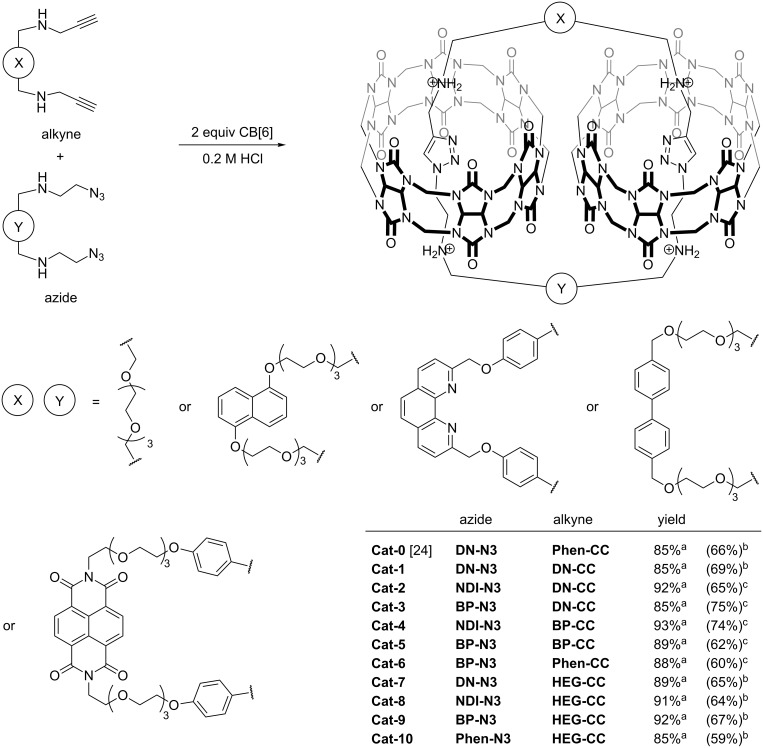
Synthesis of [3]catenanes by CBAAC. ^a^Assembly yield by HPLC; ^b^isolated yield by precipitation as PF_6_^−^ salt; ^c^isolated yield by preparative HPLC.

**Figure 1:**
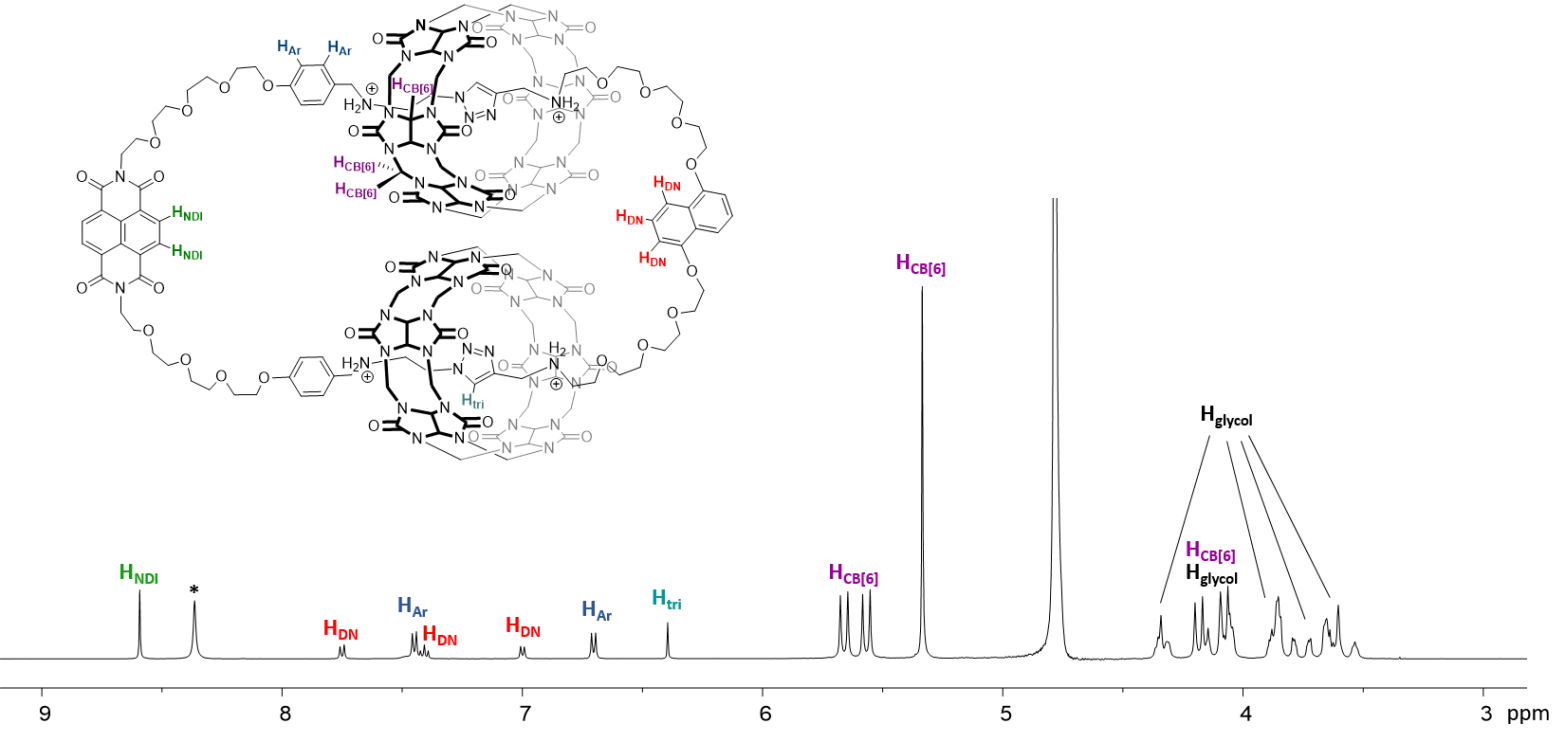
^1^H NMR (500 MHz, D_2_O, 298 K) of **Cat-2**. The signal at ca. 8.3 ppm is the residual formate from preparative HPLC.

**Figure 2:**
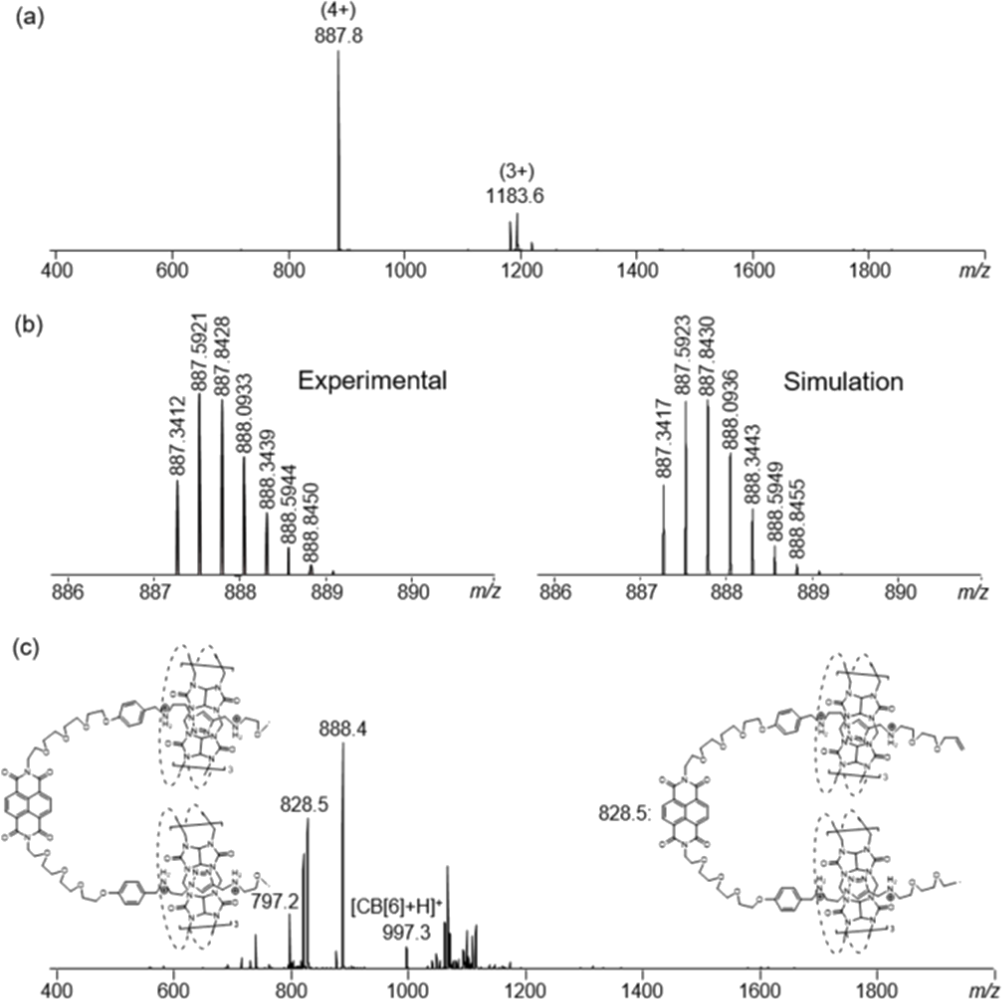
(a) ESIMS, (b) HRMS, and (c) MS^2^ spectrum (parent ion at *m/z* = 887.8) of **Cat-2**.

**Scheme 4:**
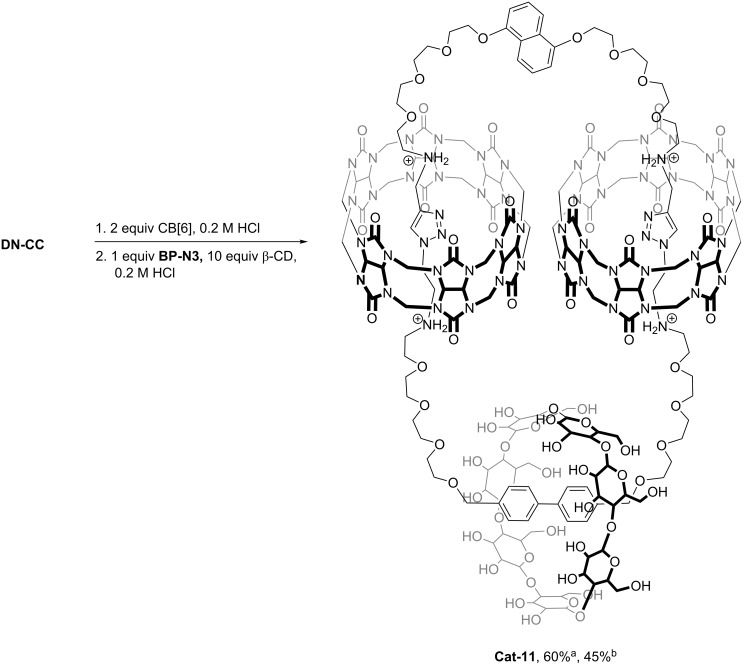
Synthesis of the [4]catenane **Cat-11**. ^a^Assembly yield by HPLC; ^b^isolated yield by preparative HPLC.

With the different combinations of the additional recognition units (i.e., DN, NDI, Phen and BP) in these [3]catenanes, other macrocycles/building blocks that could favorably interact with these units could be introduced to give higher-order catenanes with multiple interlocked rings. As a preliminary study of high-order [*n*]catenane assembly using this approach, CBAAC of **BP-N3** and **DN-CC** was repeated in the presence of 10 equivalents of β-cyclodextrin (β-CD), which is known to form a stable inclusion complex with the BP unit due to hydrophobic effects (*K*_a_ ≈ 7 × 10^3^ M^−1^ in D_2_O) [26] (Scheme 4). To our delight, a [4]catenane (**Cat-11**) was obtained in 60% yield along with 30% of **Cat-3**. Of note, due to a much weaker binding between the 1,5-dioxynaphthalene unit and β-CD [26–27], no [5]catenane was observed. ESIMS and MS^2^ spectra of **Cat-11** are consistent with the inclusion of one β-CD (and two CB[6]). The ^1^H NMR spectrum showed a slight upfield shift of the BP protons to 7.61 and 7.48 ppm when compared with that of **Cat-3** at 7.75 and 7.54 ppm. These observations are all consistent with a [4]catenane structure with the BP unit being included in the cavity of the β-CD, which is further supported by the appearance of the BP protons as two broad signals due to their slightly different chemical environment inside the β-CD (Supporting Information File 1, Figure S56). Together with our previous demonstration of the compatibility of CBAAC with Cu^+^-phenanthroline coordination in a [6]catenane assembly, the successful synthesis of **Cat-11** shows the feasibility of using CBAAC with a versatile selection of building blocks and non-covalent interactions to construct complex high-order [*n*]catenanes.

## Conclusion

In summary, the use of CBAAC in the efficient [3]catenane syntheses is described. Ten new [3]catenanes and a [4]catenane were obtained in a simple one-pot procedure. The key feature of this strategy is that the use of CB[6] couples the mechanical interlocking with the covalent macrocycle formation, so that the catenanes were formed exclusively with no formation of other topological isomers. This CBAAC strategy is versatile and building blocks containing various recognition units can be used, therefore offering a convenient entry point to access more complex high-order catenanes with multiple interlocked macrocycles.

## Supporting Information

File 1 Detailed experimental procedures and characterization data (MS, MS^2^, ^1^H and ^13^C NMR and UV–vis spectra).

## References

[1] Sauvage J-P (1998). Acc Chem Res.

[2] Nepogodiev S A, Stoddart J F (1998). Chem Soc Rev.

[3] Erbas-Cakmak S, Leigh D A, McTernan C T, Nussbaumer A L (2015). Chem Rev.

[4] Evans N H, Beer P D (2014). Chem Soc Rev.

[5] Dietrich-Buchecker C O, Sauvage J P (1987). Chem Rev.

[6] Fang L, Olson M A, Benítez D, Tkatchouk E, Goddard W A, Stoddart J F (2010). Chem Soc Rev.

[7] Beves J E, Blight B A, Campbell C J, Leigh D A, McBurney R T (2011). Angew Chem, Int Ed.

[8] Forgan R S, Sauvage J-P, Stoddart J F (2011). Chem Rev.

[9] Gil-Ramírez G, Leigh D A, Stephens A J (2015). Angew Chem, Int Ed.

[10] Niu Z, Gibson H W (2009). Chem Rev.

[11] Raehm L, Hamilton D G, Sanders J K M (2002). Synlett.

[12] Wu Q, Rauscher P M, Lang X, Wojtecki R J, de Pablo J J, Hore M J, Rowan S J (2017). Science.

[13] Amabilino D B, Ashton P R, Boyd S E, Lee J Y, Menzer S, Stoddart J F, Williams D J (1997). Angew Chem, Int Ed Engl.

[14] Bitsch F, Dietrich-Buchecker C O, Khemiss A K, Sauvage J P, Van Dorsselaer A (1991). J Am Chem Soc.

[15] Li S, Huang J, Zhou F, Cook T R, Yan X, Ye Y, Zhu B, Zheng B, Stang P J (2014). J Am Chem Soc.

[16] Langton M J, Matichak J D, Thompson A L, Anderson H L (2011). Chem Sci.

[17] Iwamoto H, Tafuku S, Sato Y, Takizawa W, Katagiri W, Tayama E, Hasegawa E, Fukazawa Y, Haino T (2016). Chem Commun.

[18] Black S P, Stefankiewicz A R, Smulders M M J, Sattler D, Schalley C A, Nitschke J R, Sanders J K M (2013). Angew Chem, Int Ed.

[19] Au-Yeung H Y, Yee C-C, Ng A W H, Hu K (2018). Inorg Chem.

[20] Crowley J D, Goldup S M, Lee A-L, Leigh D A, McBurney R T (2009). Chem Soc Rev.

[21] Tuncel D, Özsar Ö, Tiftik H B, Salih B (2007). Chem Commun.

[22] Sinha M K, Reany O, Yefet M, Botoshansky M, Keinan E (2012). Chem – Eur J.

[23] Ke C, Smaldone R A, Kikuchi T, Li H, Davis A P, Stoddart J F (2013). Angew Chem, Int Ed.

[24] Wang K, Yee C-C, Au-Yeung H Y (2016). Chem Sci.

[25] Celtek G, Artar M, Scherman O A, Tuncel D (2009). Chem – Eur J.

[26] Armspach D, Ashton P R, Ballardini R, Balzani V, Godi A, Moore C P, Prodi L, Spencer N, Stoddart J F, Tolley M S (1995). Chem – Eur J.

[R27] 27It has been reported that the bindings of a methylated derivative of β-CD to 1,5-dioxynaphthalene derivatives were at least two order-of-magnitude weaker than that of the bindings to biphenyl guests. See reference [26] for details.

