# Supplementary material for: Efficient catenane synthesis by cucurbit[6]uril-mediated azide–alkyne cycloaddition
Source: Beilstein J Org Chem. 2018 Jul 20;14:1846–53. doi: 10.3762/bjoc.14.158 (PMC6071691; doi:10.3762/bjoc.14.158)
Supplement: File 1 — Detailed experimental procedures and characterization data (MS, MS2, 1H and 13C NMR and UV–vis spectra). [file Beilstein_J_Org_Chem-14-1846-s001.pdf]

Supporting Information
for
Efficient catenane synthesis by cucurbit[6]uril-mediated azide–alkyne cycloaddition

Antony Wing Hung Ng, Chi-Chung Yee, Kai Wang and Ho Yu Au-Yeung*

Address: Department of Chemistry, The University of Hong Kong, Pokfulam Road, Hong Kong, P. R. China

Email: Ho Yu Au-Yeung - hoyuay@hku.hk

*Corresponding author

Detailed experimental procedures and characterization data (MS, MS^2, ^1H and ^{13}C NMR and UV–vis spectra)

Table of Contents

1. Synthesis S2
2. HPLC analysis S18
3. NMR S24
4. ESIMS S53
5. UV–vis S64
6. References S70

1. Synthesis

General. All reagents were purchased from commercial suppliers (Aldrich, Dkmchem and J & K) and used without further purification. All solvents for syntheses were of analytical grade (ACI Labscan and DUKSAN Pure Chemicals). MeCN, $CHCl_3$ and MeOH were distilled over CaH_2 before use. **DN-OTs**,[1] **DN-N3**,[2] **NDI-Br**,[3] **BP-OTs**,[4] **Phen-CHO**,[5] **Phen-CC**,[5] 2-azidoethylamine[6] and cucurbit[6]uril (CB[6]) were synthesized according to literature procedures. Microwave-assisted reactions were carried out using a Discover SP microwave synthesizer (CEM, USA) in the closed vessel focused single. Thin layer chromatography (TLC) was performed on silica gel 60 F254 (Merck, Germany, aluminium sheet) and column chromatography was carried out on silica gel 60F (Silicycle, Canada). HPLC analyses were carried out using a Waters-Alliance e2695 system coupled to a 2489 UV–vis detector. ESIMS analyses were carried out using a Waters-Acquity UPLC H-Class system coupled with a QDa MS detector. NMR spectra were recorded on Bruker DPX spectrometers with working frequencies of 400 MHz or 500 MHz for ^1H, and 100 MHz or 125 MHz for ^{13}C, respectively. Chemical shifts are reported in ppm and referenced to residual solvent signals (for ^1H: $CDCl_3$: δ = 7.26 ppm, DMSO: δ = 2.50 ppm, D_2O: δ = 4.79 ppm, CD_3CN: δ = 1.94 ppm; For ^{13}C: $CDCl_3$: δ = 77.16 ppm, DMSO: δ = 39.52 ppm, CD_3CN: δ = 118.26 ppm).

A. Building block synthesis

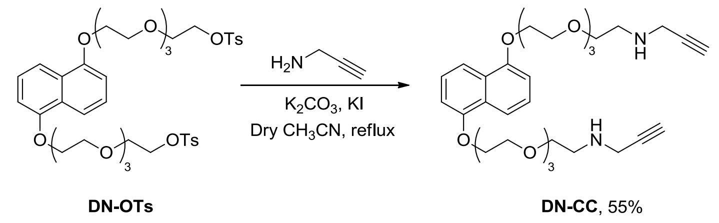

DN-CC. A mixture of propargylamine (550 mg, 10.0 mmol), K_2CO_3 (830 mg, 6.0 mmol) and KI (33.0 mg, 0.2 mmol) in dried CH_3CN (2 mL) was heated to reflux. A solution of **DN-OTs**[1] (820 mg, 1.00 mmol, 10 mL) in the same solvent was added via a syringe pump at 1 mL/h. Heating was continued overnight. The solvents were removed using a rotary evaporator and the residue partitioned between CH_2Cl_2 (20 mL) and water (10 mL). The aqueous layer was extracted with CH_2Cl_2 (2 × 20 mL). The organic fractions were combined and washed with brine, dried over $MgSO_4$, concentrated and purified by column chromatography on silica ($CH_2Cl_2/CH_3OH/Et_3N$ 120:3:0.5 to 120:5:0.5). A light brown oil was obtained. Yield = 311 mg, 55%. ^{1}H NMR (400 MHz, $CDCl_3$, 298 K) δ = 7.85 (d, *J* = 8.0 Hz, 2H), 7.33 (t, *J* = 8.0 Hz, 2H), 6.83 (d, *J* = 8.0 Hz, 2H), 4.28 (t, *J* = 4.9 Hz, 4H), 3.99 (t, *J* = 4.9 Hz, 4H), 3.81–3.78 (m, 4H), 3.70–3.64 (m, 8H), 3.62–3.57 (m, 8H), 3.42 (d, *J* = 2.5 Hz, 4H), 2.84 (t, *J* = 5.2 Hz, 4H), 2.19 (t, *J* = 2.4 Hz, 2H). ^{13}C{^{1}H} NMR (100 MHz, $CDCl_3$, 298 K) δ = 154.4, 126.9, 125.2, 114.7, 105.7, 82.2, 71.5, 71.1, 70.8, 70.7, 70.5, 70.4, 70.0, 68.0, 48.1, 38.3. ESI-MS: 587.6 $[M+H]^+$.

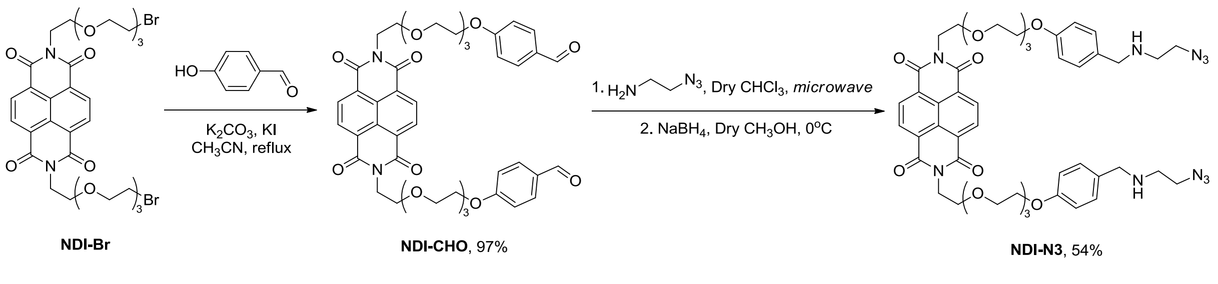

NDI-CHO. A mixture of 4-hydroxylbenzaldehyde (32.0 mg, 0.3 mmol), K_2CO_3 (73.0 mg, 0.5 mmol), KI (5.0 mg, 0.03 mmol) and **NDI-Br**[3] (94.0 mg, 0.1 mmol) in CH_3CN (20 mL) was heated to reflux overnight. The solvents were removed using a rotary evaporator and the residue partitioned between CH_2Cl_2 (15 mL) and water (15 mL). The aqueous layer was extracted with CH_2Cl_2 (2 × 15 mL). The organic

fractions were combined and washed with brine, dried over $MgSO_4$ and concentrated. A pale yellow solid was obtained. Yield = 101 mg, 97%. ^{1}H NMR (500 MHz, $CDCl_3$, 298 K) δ = 9.86 (s, 2H), 8.73 (s, 4H), 7.79 (d, *J* = 8.0 Hz, 4H), 6.99 (d, *J* = 8.0 Hz, 4H), 4.45 (t, *J* = 6.0 Hz, 4H) 4.18 (t, *J* = 5.9 Hz, 4H), 3.86–3.83 (m, 8H), 3.70 (t, *J* = 6.0 Hz, 4H), 3.66 (t, J = 6.1 Hz, 4H), 3.64–3.62 (m, 8H) ^{13}C{^{1}H} NMR (100 MHz, $CDCl_3$, 298 K) δ = 190.8, 163.8, 162.8, 131.9, 130.9, 130.0, 126.6, 126.5, 114.8, 70.8, 70.6, 70.6, 70.1, 69.4, 67.7, 53.5, 39.6 ESI-MS: 849.5 $[M+Na]^+$.

NDI-N3. A mixture of **NDI-CHO** (500 mg, 0.6 mmol) and 2-azidoethylamine[6] (520 mg, 6.0 mmol) in dry $CHCl_3$ (30 mL) was sonicated until a homogeneous solution was formed. Then the reaction mixture was heated under microwave irradiation for 5 min at 115 °C, and cooled to 0 °C in an ice bath. $NaBH_4$ (90.0 mg, 2.4 mmol) was added in portions, followed by the addition of dry CH_3OH (18 mL). The mixture was stirred at 0 °C for 2 h. The solvents were removed using a rotary evaporator and the residue partitioned between CH_2Cl_2 (20 mL) and 2 M aq. NaOH (15 mL). The aqueous layer was extracted with CH_2Cl_2 (2 × 15 mL) and the organic fractions were combined, washed with brine, dried over $MgSO_4$, concentrated and purified by column chromatography on silica ($CH_2Cl_2/CH_3OH/Et_3N$ 95:5:1). A dark yellow oil was obtained. Yield = 318 mg, 54%. ^{1}H NMR (500 MHz, $CDCl_3$, 298 K) δ = 8.73 (s, 4H), 7.20 (d, *J* = 8.0 Hz, 4H), 6.84 (d, *J* = 8.0 Hz, 4H), 4.45 (t, *J* = 6.0 Hz, 4H), 4.07 (t, *J* = 6.2 Hz, 4H), 3.85 (t, *J* = 5.8 Hz, 4H), 3.80 (t, *J* = 6.1 Hz, 4H), 3.73 (s, 4H), 3.70 (t, *J* = 5.3 Hz, 4H), 3.65–3.62 (m, 12H), 3.40 (t, *J* = 6.0 Hz, 4H), 2.81 (t, *J* = 6.4 Hz, 4H) ^{13}C{^{1}H} NMR (100 MHz, $CDCl_3$, 298 K) δ = 162.9, 157.9, 132.3, 131.0, 129.2, 126.7, 126.6, 114.6, 70.8, 70.6, 70.2, 69.7, 67.8, 67.4, 53.6, 53.0, 51.5, 47.9, 39.6 ESI-MS: 484.5 $[M+2H]^{2+}$.

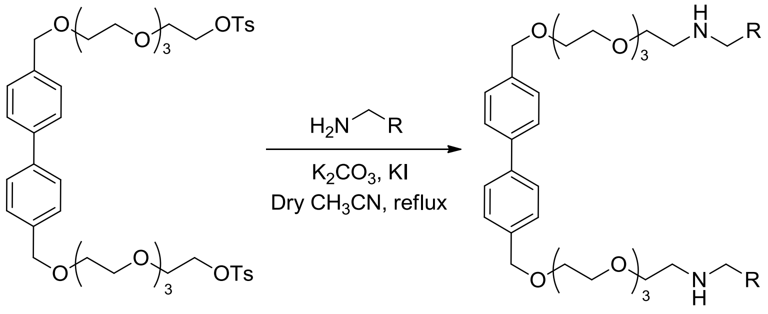

BP-OTs

BP-N3: R = CH_2N_3, 73%

BP-CC: R = C≡CH, 61%

BP-N3. A mixture of 2-azidoethylamine[6] (860 mg, 10.0 mmol), K_2CO_3 (830 mg, 6.0 mmol) and KI (83.0 mg, 0.5 mmol) in dry CH_3CN (5 mL) was heated to reflux. A solution of **BP-OTs**[4] (1.75 g, 2.0 mmol, 20 mL) in the same solvent was added over 2 h and heating was continued overnight. The solvents were removed using a rotary evaporator and the residue partitioned between CH_2Cl_2 (40 mL) and water (20 mL). The aqueous layer was extracted with CH_2Cl_2 (2 × 40 mL) and the organic fractions were combined, washed with brine, dried over $MgSO_4$, concentrated and purified by column chromatography on silica ($CH_2Cl_2/CH_3OH/Et_3N$ 100:3:0.5 to 100:5:0.5). A pale yellow oil was obtained. Yield = 1.02 g, 73%. ^{1}H NMR (100 MHz, $CDCl_3$, 298 K) δ = 7.56 (d, J = 8.0 Hz, 4H), 7.41 (d, J = 8.0 Hz, 1H), 4.60 (s, 4H), 3.69–3.63 (m, 24H), 3.59 (t, J = 5.1 Hz, 4H), 3.42 (t, J = 5.9 Hz, 4H), 2.84–2.78 (m, 8H). $^{13}C\{^1H\}$ NMR (100 MHz, $CDCl_3$, 298 K) δ = 140.4, 137.4, 128.4, 127.2, 73.1, 70.8, 70.8, 70.7, 70.7, 70.5, 70.4, 69.6, 51.3, 49.1, 48.5. ESI-MS: 703.8 $[M+H]^+$

BP-CC. A mixture of propagylamine (550 mg, 10.0 mmol), K_2CO_3 (830 mg, 6.0 mmol) and KI (83.0 mg, 0.5 mmol) in dry CH_3CN (3 mL) was heated to reflux. A solution of **BP-OTs**[4] (0.88 g, 1.0 mmol, 15 mL) in the same solvent was added over 2 h and heating was continued overnight. The solvents were removed using a rotary evaporator and the residue partitioned between CH_2Cl_2 (30 mL) and water (15 mL). The aqueous layer was extracted with CH_2Cl_2 (2 × 40 mL) and the organic fractions were combined, washed with brine, dried over $MgSO_4$, concentrated and purified by column chromatography on silica ($CH_2Cl_2/CH_3OH/Et_3N$ 100:3:0.5 to 100:5:0.5). A light yellow oil was obtained. Yield = 390 mg, 61%.^{1}H NMR (400 MHz, $CDCl_3$, 298 K) δ = 7.56 (d, J = 8.3 Hz, 4H), 7.41 (d, J = 8.3 Hz, 4H), 4.60 (s, 4H), 3.71–3.60 (m, 32H), 3.45 (d, J = 2.5 Hz, 4H), 2.90–2.85 (t, J = 5.2 Hz, 4H), 2.21 (t, J = 2.4 Hz, 2H).

$^{13}C\{^1H\}$ NMR (125 MHz, $CDCl_3$, 298 K) δ = 140.1, 137.2, 128.1, 127.0, 81.7, 72.8, 71.7, 70.6, 70.5, 70.5, 70.4, 70.2, 70.1, 69.4, 47.8, 38.0. ESI-MS: 641.8 $[M+H]^+$

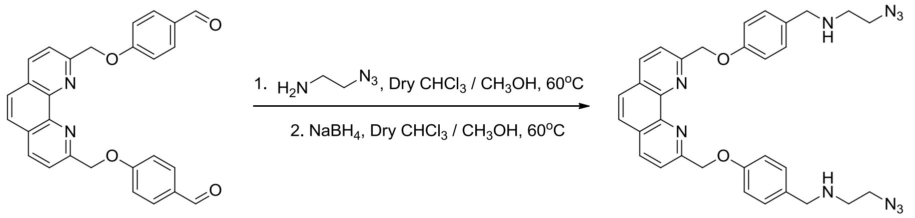

Phen-N3. A mixture of **Phen-CHO**[5] (800 mg, 1.8 mmol) and 2-azidoethylamine[6] (620 mg, 7.1 mmol) in dry $CHCl_3/CH_3OH$ (v/v 3:1, 28 mL) was heated at 60 °C overnight. The reaction mixture was cooled to room temperature and $NaBH_4$ (270 mg, 7.1 mmol) was added in portions and the mixture was heated to 60 °C overnight. The solvents were removed using a rotary evaporator and the residue partitioned between CH_2Cl_2 (20 mL) and water (10 mL). The aqueous layer was extracted with CH_2Cl_2 (2 × 20 mL) and the organic fractions were combined and washed with brine, dried over $MgSO_4$, concentrated and purified by column chromatography on silica ($CH_2Cl_2/CH_3OH/Et_3N$ 99:1:0.5). A yellow-orange solid was obtained. Yield = 650 mg, 62%. ^1H NMR (400 MHz, $CDCl_3$, 298 K) δ = 8.20 (d, *J* = 8.3 Hz, 2H), 7.87 (d, *J* = 8.3 Hz, 2H), 7.70 (s, 2H), 7.22 (d, *J* = 8.4 Hz, 4H), 7.00 (d, *J* = 8.4 Hz, 4H), 5.59 (s, 4H), 3.69 (s, 4H), 3.36 (t, *J* = 5.70 Hz, 4H), 2.74 (t, *J* = 5.0 Hz, 4H). $^{13}C\{^1H\}$ NMR (100 MHz, $CDCl_3$, 298 K) δ = 158.2, 157.5, 145.0, 137.0, 132.5, 129.3, 128.1, 126.2, 120.8, 114.8, 71.3, 52.8, 51.3, 47.7. ESI-MS: 589.6 $[M+H]^+$.

B. [3]Catenane synthesis

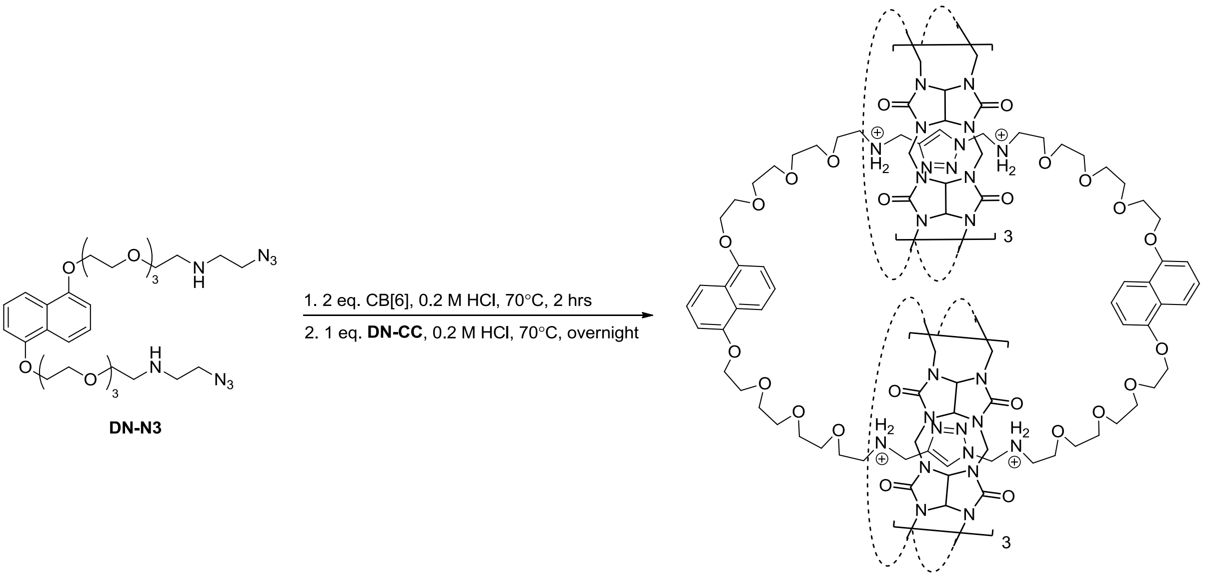

Cat-1, 85%

Cat-1. A mixture of **DN-N3**[2] (33.0 mg, 0.05 mmol) and CB[6] (100 mg, 0.1 mmol) in 0.2 M HCl (40 mL) was heated at 70 °C for 2 h until a clear solution was obtained. A solution of **DN-CC** (30.0 mg, 0.05 mmol, 40 mL) in the same solvent was added over 2 h and the reaction mixture was heated to 70 °C overnight. HPLC analysis showed that ca. 85% of the materials in the product mixture corresponded to **Cat-1**. Saturated aq. NH_4PF_6 (5 mL) was added and the white precipitate was collected by filtration, washed with water (3 × 5 mL) and dried in vacuum. Isolated yield = 131 mg, 69%. ^{1}H NMR (400 MHz, $(CD_3)_2SO$, 298 K) δ = 7.73 (m, 4H), 7.71 (s, 4H), 7.37 (m, 4H), 7.15 (s, 4H), 6.98 (m, 4H), 6.40 (s, 2H), 5.56-5.36 (m, 48H), 4.28-4.25 (m, 40H), 3.93 (m, 4H), 3.90 (m, 16H), 3.69–3.60 (m, 32H), 3.42 (m, 4H). ^{13}C{^{1}H} NMR (100 MHz, $(CD_3)_2SO$, 298 K) δ = 155.4, 155.4, 155.0, 153.9, 137.8, 125.9, 125.4, 120.6, 113.8, 106.0, 70.1, 69.9, 69.8, 69.0, 67.7, 65.5, 64.8, 50.6, 50.4, 47.8, 46.8, 46.1, 46.0, 41.5. ESI-MS: 808.5 $[M+4H]^{4+}$ 1077.3 $[M+3H]^{3+}$.

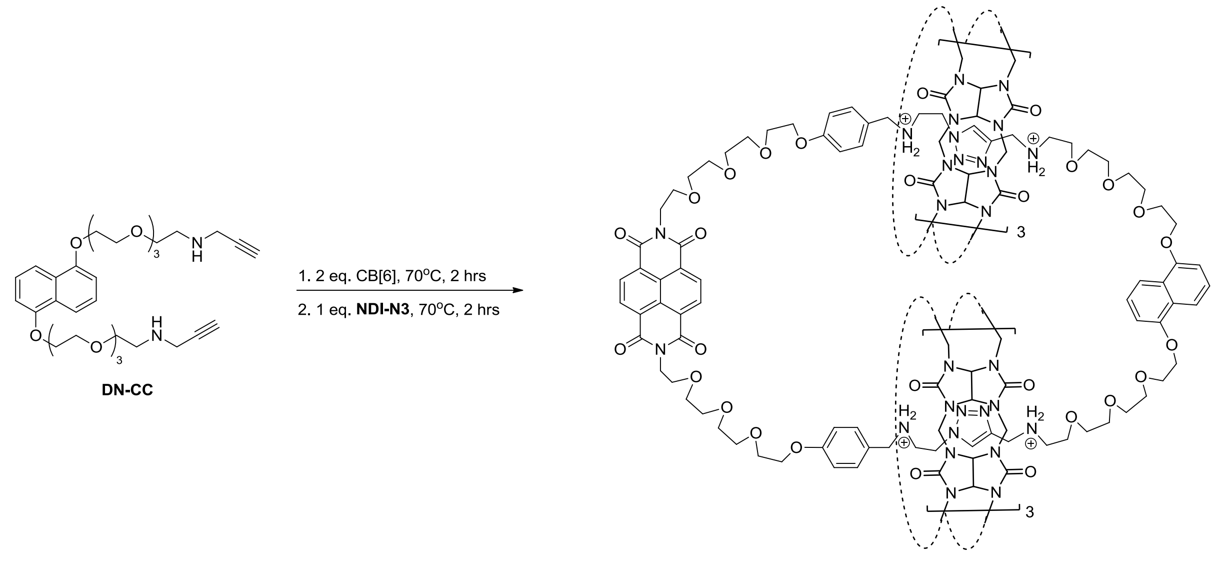

Cat-2, 92%

Cat-2. A mixture of **DN-CC** (25.0 mg, 0.04 mmol) and CB[6] (80.0 mg, 0.08 mmol) in 0.2 M HCl (22 mL) was heated to 70 °C for 2 h until a clear solution was obtained. A solution of **NDI-N3** (41.0 mg, 0.04 mmol, 22 mL) in the same solvent was added over 2 h and the reaction mixture was heated to 70 °C overnight. HPLC analysis showed that ca. 92% of the materials in the product mixture corresponded to **Cat-2**. **Cat-2** was isolated from the crude mixture by preparative HPLC (see details below). The mixture was concentrated to 2 mL and 0.2 mL of this solution was injected onto the preparative column. Isolated yield = 10 mg, 65% ^{1}H NMR (500 MHz, D_2O, 298 K) δ = 8.57 (s, 4H), 7.75 (d, *J* = 5.0 Hz, 2H), 7.45 (d, *J* = 6.8 Hz, 4H), 7.41, (t, *J* = 5.0 Hz, 2H), 7.00 (d, *J* = 5.0 Hz, 2H), 6.71 (d, *J* = 6.8 Hz, 4H), 6.4 (s, 2H), 5.68 (d, *J* = 15.6 Hz, 12H), 5.55 (d, *J* = 15.5 Hz, 12H), 5.34 (s, 24H), 4.35–4.31 (m, 12H), 4.20–4.15 (m, 16H), 4.09–4.06 (m, 24H), 3.88–3.84 (m, 20H), 3.80–3.79 (m, 4H), 3.73–3.72 (m, 4H), 3.65–3.60 (m, 20H), 3.54–3.53 (m, 4H). ^{13}C{^{1}H} NMR (125 MHz, D_2O, 298 K) δ = 169.5, 164.2, 158.6, 156.3, 156.1, 153.9, 138.9, 131.5, 131.2, 126.2, 124.4, 119.9, 114.9, 114.4, 106.9, 70.4, 70.1, 70.0, 69.8, 69.4, 68.9, 68.2, 67.5, 67.2, 65.3, 51.4, 51.3, 50.6, 48.4, 45.7, 45.0, 42.4, 39.7. ESI-MS: 887.6 $[M+4H]^{4+}$ 1183.1 $[M+3H]^{3+}$.

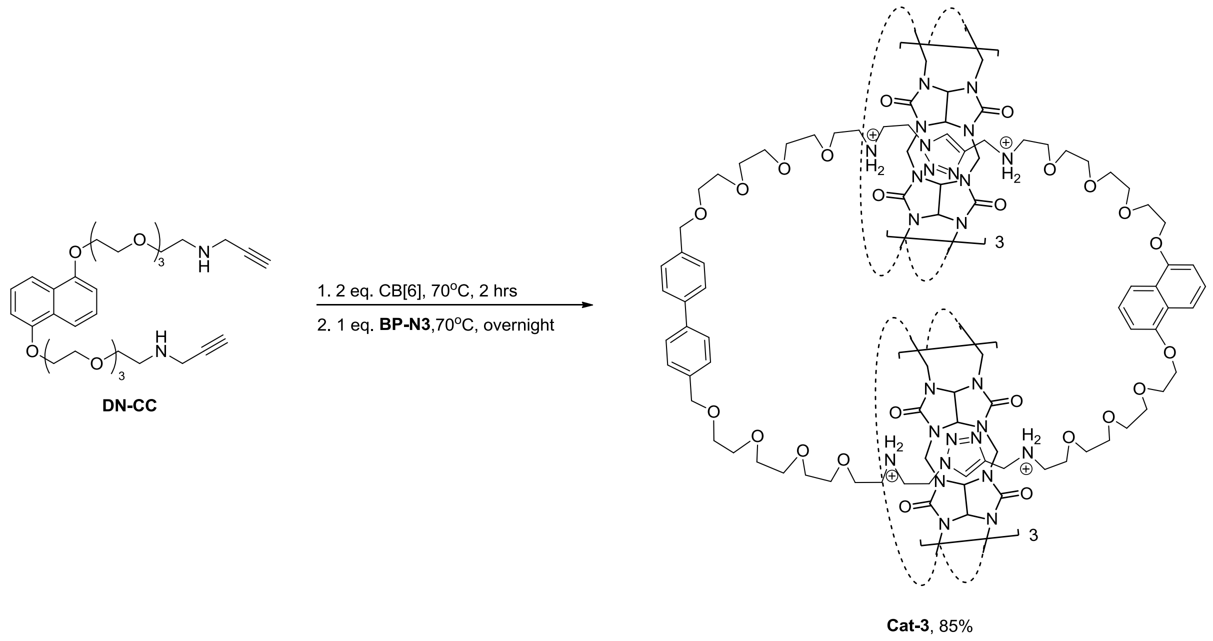

Cat-3. A mixture of **DN-CC** (25.0 mg, 0.04 mmol) and CB[6] (80.0 mg, 0.08 mmol) in 0.2 M HCl (22 mL) was heated to 70 °C for 2 h until a clear solution was obtained. A solution of **BP-N3** (30.0 mg, 0.04 mmol, 22 mL) in the same solvent was added over 2 h and the reaction mixture was heated to 70 °C overnight. HPLC analysis showed that ca. 85% of the materials in the product mixture corresponded to **Cat-3**. **Cat-3** was isolated from the crude mixture by preparative HPLC. The reaction mixture was concentrated to 2 mL and 0.2 mL of this solution was injected onto the preparative column. Isolated yield = 11mg, 75%. ^{1}H NMR (500 MHz, D_2O, 298 K) δ = 7.90 (d, *J* = 5.0 Hz, 2H), 7.75 (d, *J* = 7.4 Hz, 4H), 7.54 (d, *J* = 7.4 Hz, 4H), 7.47 (t, *J* = 5.0 Hz, 2H), 7.08 (d, *J* = 5.0 Hz, 2H), 6.38 (s, 2H), 5.65 (d, *J* = 15.6 Hz, 12H), 5.55 (d, *J* = 15.5 Hz, 12H), 5.24 (s, 24H), 4.64 (s, 4H), 4.37–4.36 (m, 4H), 4.11–4.01 (m, 40H), 3.85–3.75 (m, 28H), 3.73–3.65 (m, 16H), 3.63–3.61 (m, 4H), 3.59–3.58 (m, 4H), 3.56–3.55 (m, 4H). ^{13}C{^{1}H} NMR (125 MHz, D_2O, 298 K) δ = 156.1, 155.9, 154.0, 138.8, 138.7, 137.3, 129.3, 126.5, 126.2, 126.1, 119.8, 114.4, 106.8, 72.5, 70.0, 69.9, 69.8, 69.7, 69.6, 69.5, 69.4, 69.4, 69.2, 68.2, 65.8, 65.2, 51.3, 51.1, 48.3, 47.3, 45.7, 45.1, 42.2. ESI-MS: 821.4 $[M+4H]^{4+}$ 1095.0 $[M+3H]^{3+}$

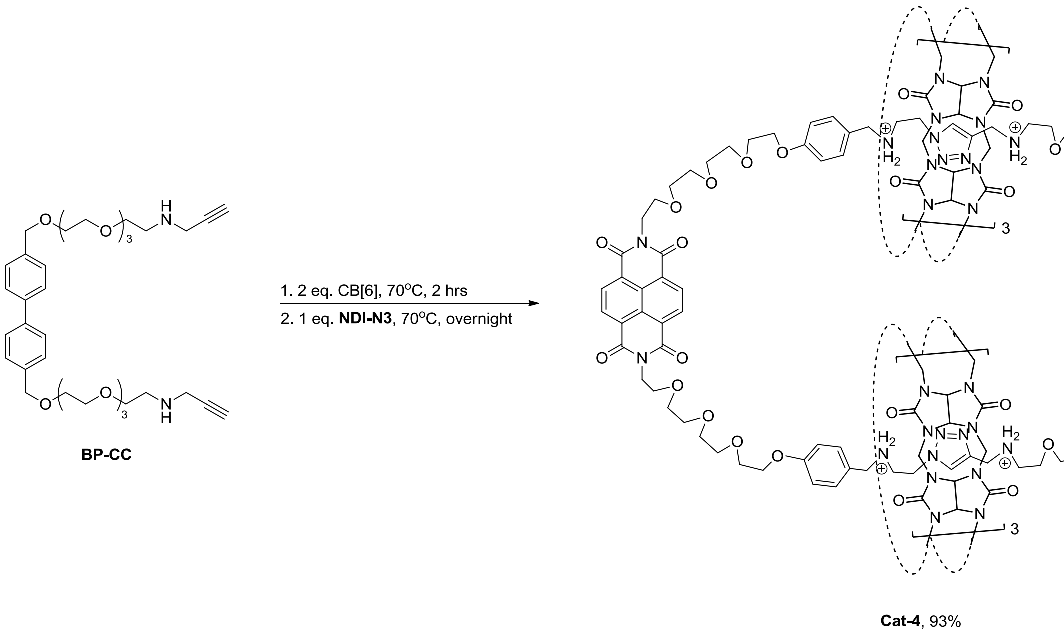

Cat-4. A mixture of **BP-CC** (28.0 mg, 0.04 mmol) and CB[6] (80.0 mg, 0.08 mmol) in 0.2 M HCl (22 mL) was heated to 70 °C for 2 h until a clear solution was obtained. A solution of **NDI-N3** (41.0 mg, 0.04 mmol, 22 mL) in the same solvent was added over 2 h and the reaction mixture was heated to 70 °C overnight. HPLC analysis showed that ca. 93% of the materials in the product mixture corresponded to **Cat-4**. **Cat-4** was isolated from the crude mixture by preparative HPLC. The reaction mixture was concentrated to 2 mL and 0.1 mL of this solution was injected onto the preparative column. Isolated yield = 6 mg, 74%. ^{1}H NMR (500 MHz, D_2O, 298 K) δ = 8.60 (s, 4H), 7.74 (d, *J* = 7.5 Hz, 4H), 7.52 (d, J = 7.5 Hz, 4H), 7.46 (d, J = 10.0 Hz, 4H), 6.72 (d, J = 10.0 Hz, 4H), 6.44 (s, 2H), 5.69 (d, *J* = 15.6 Hz, 12H), 5.62 (d, *J* = 15.5 Hz, 12H), 5.39 (s, 24H), 4.63 (s, 4H), 4.35–4.34 (m, 4H), 4.21–4.17 (m, 28H), 4.13–4.11 (m, 8H), 3.73–3.71 (m, 20H), 3.67–3.63 (m, 40H), 3.62–3.61 (m, 4H), 3.31–3.30 (m, 4H). ^{13}C{^{1}H} NMR (125 MHz, D_2O, 298 K) δ = 156.7, 156.2, 156.0, 155.9, 155.5, 154.4, 131.9, 131.6, 131.1, 128.9, 128.3, 127.1, 126.5, 126.2, 111.1, 73.1, 72.6, 70.7, 70.4, 70.4, 69.7, 69.6, 69.3, 67.6, 67.3, 67.1, 65.7, 51.8, 51.5, 51.4, 51.1, 46.2, 46.0, 45.7, 39.9. ESI-MS: 901.4 $[M+4H]^{4+}$ 1201.5 $[M+3H]^{3+}$

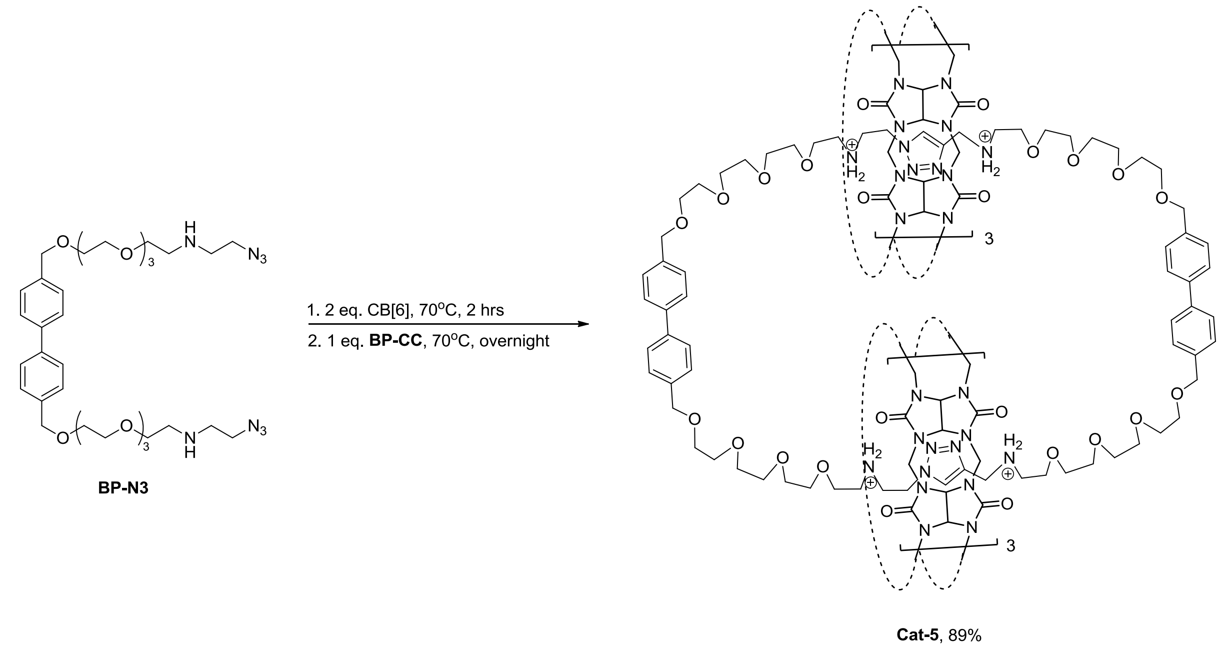

Cat-5. A mixture of **BP-N3** (21.0 mg, 0.03 mmol) and CB[6] (60.0 mg, 0.06 mmol) in 0.2 M HCl (25 mL) was heated to 70 °C for 2 h until a clear solution was obtained. A solution of **BP-CC** (20.0 mg, 0.03 mmol, 25 mL) in the same solvent was added over 2 h and the reaction mixture was heated to 70 °C overnight. HPLC analysis showed that *ca*. 89% of the materials in the product mixture corresponds to **Cat-5**. **Cat-5** was isolated from the crude mixture by preparative HPLC. The reaction mixture was concentrated to 5 mL and 1 mL of this solution was injected onto the preparative column. Isolated yield = 12 mg, 62%. ^{1}H NMR (500 MHz, D_2O, 298 K) δ = 7.74 (d, *J* = 8.0 Hz, 4H), 7.71 (d, *J* = 8.0 Hz, 4H), 7.53 (d, *J* = 8.0 Hz, 8H), 6.40 (s, 2H), 5.63 (d, *J* = 15.6 Hz, 12H), 5.59 (d, *J* = 15.5 Hz, 12H), 5.29 (s, 24H), 4.64 (s, 4H), 4.63 (s, 4H), 4.13 (d, *J* = 7.9 Hz, 12H), 4.10–4.06 (m, 24H), 4.00 (t, *J* = 5.0 Hz, 4H), 3.87–3.84 (m, 4H), 3.83 (d, *J* = 5.4 Hz, 4H), 3.81–3.77 (m, 16H), 3.73 (t, *J* = 8.3 Hz, 28H), 3.65 (d, *J* = 6.3 Hz, 4H), 3.58 (t, *J* = 5.1 Hz, 4H), 3.52 (t, *J* = 5.1 Hz, 4H). ^{13}C{^{1}H} NMR (125 MHz, D_2O, 298 K) δ = 156.2, 155.9, 138.9, 138.8, 137.4, 137.4, 129.3, 129.2, 126.6, 126.5, 119.9, 72.5, 72.5, 70.1, 70.0, 69.9, 69.8, 69.8, 69.7, 69.7, 69.6, 69.6, 69.5, 69.4, 65.8, 65.3, 61.0, 51.3, 51.1, 48.3, 47.3, 45.7, 45.1, 42.2. ESI-MS: 835.2 $[M+4H]^{4+}$ 1113.4 $[M+3H]^{3+}$

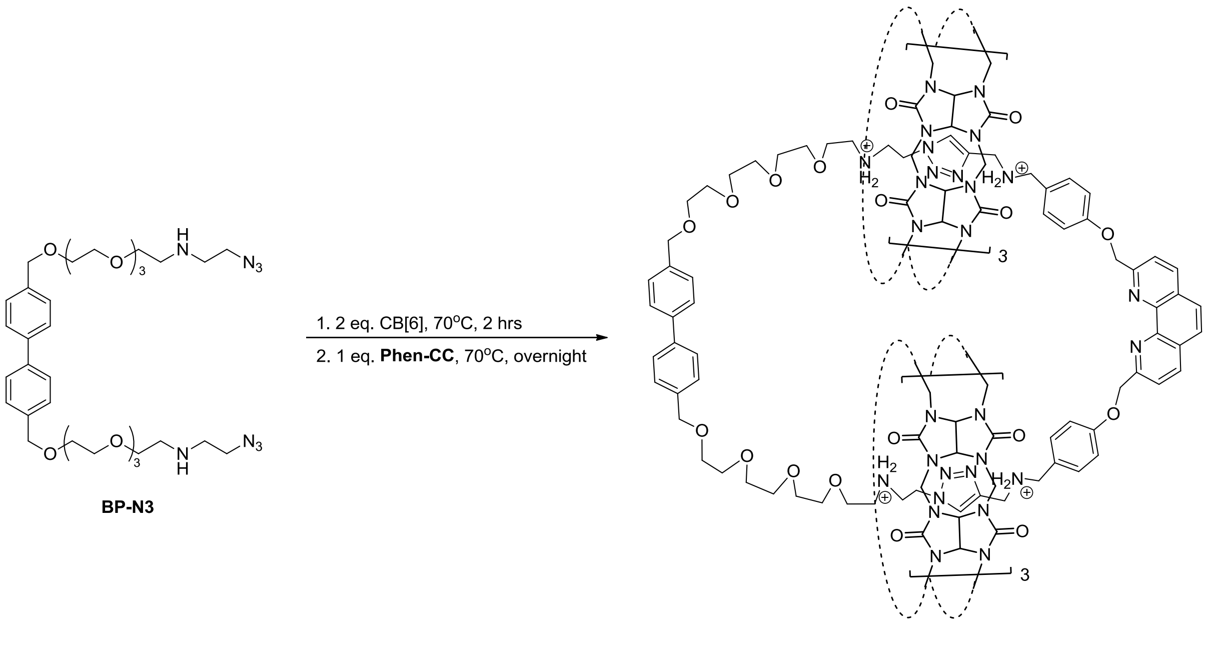

Cat-6. A mixture of **BP-N3** (21.0 mg, 0.03 mmol) and CB[6] (60.0 mg, 0.06 mmol) in 0.2 M HCl (25 mL) was heated to 70 °C for 2 h until a clear solution was obtained. A solution of **Phen-CC**[5] (16.0 mg, 0.03 mmol, 25 mL) in the same solvent was added over 2 h and the reaction mixture was heated to 70 °C overnight. HPLC analysis showed that ca. 88% of the materials in the product mixture corresponded to **Cat-6**. **Cat-6** was isolated from the crude mixture by preparative HPLC. The reaction mixture was concentrated to 5 mL and 0.6 mL of this solution was injected onto the preparative column. Isolated yield = 7 mg, 60%. ^{1}H NMR (500 MHz, D_2O, 298 K) δ 8.68 (s, 1H), 8.14 (d, *J* = 8.4 Hz, 2H), 8.07 (s, 2H), 7.74 (d, *J* = 8.1 Hz, 4H), 7.71 (d, *J* = 8.5 Hz, 4H), 7.47 (d, *J* = 8.0 Hz, 4H), 7.29 (d, *J* = 8.4 Hz, 4H), 6.38 (s, 2H), 5.66 (d, *J* = 15.5 Hz, 12H), 5.48 (d, *J* = 15.5 Hz, 12H), 5.32 (s, 24H), 4.58 (s, 4H), 4.37 (s, 4H), 4.17–4.11 (m, 24H), 4.04 (d, *J* = 15.5 Hz, 6H), 4.00 (d, *J* = 4.8 Hz, 2H), 3.81 (d, *J* = 5.5 Hz, 2H), 3.78–3.76 (m, 4H), 3.71 (dd, *J* = 5.9, 2.7 Hz, 8H), 3.68 (d, *J* = 5.5 Hz, 8H), 3.57 (t, *J* = 5.1 Hz, 4H).^{13}C{^{1}H} NMR (125 MHz, D_2O, 298 K) δ = 163.0, 158.1, 156.1, 155.9, 140.5, 138.7, 137.2, 136.1, 132.5, 130.8, 129.3, 128.7, 126.6, 124.2, 120.0, 115.6, 72.5, 70.0, 69.8, 69.6, 69.6, 69.5, 69.4, 65.8, 51.9, 51.3, 51.0, 47.4, 46.5, 45.8, 45.2, 44.1, 42.0. ESI-MS: 806.5 $[M+4H]^{4+}$ 1075.0 $[M+3H]^{3+}$

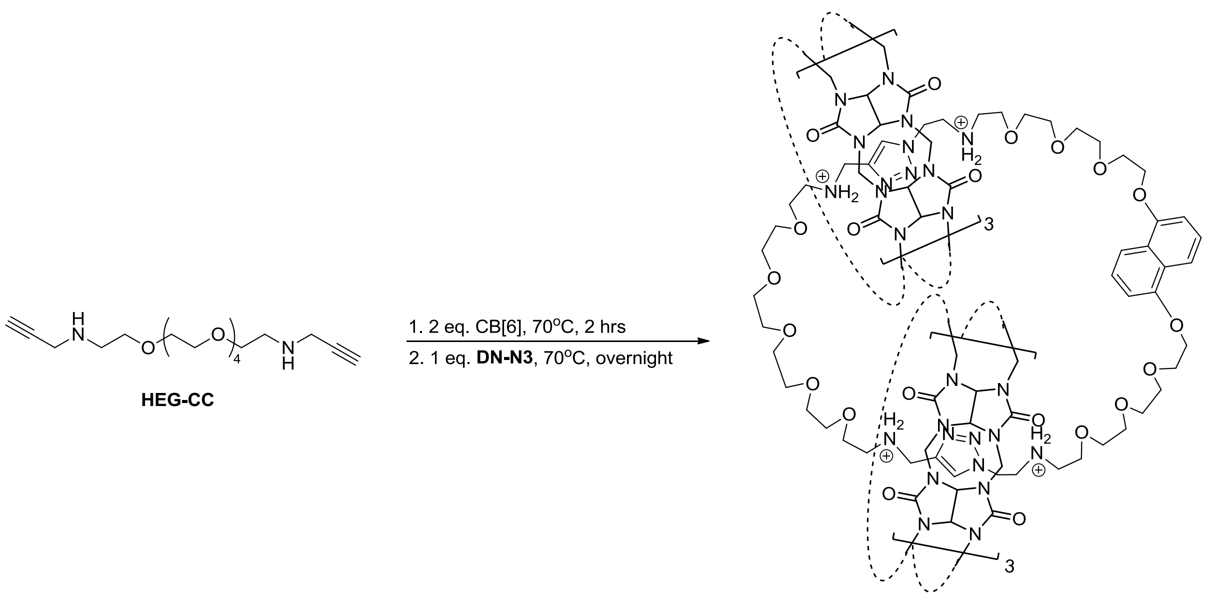

Cat-7, 89%

Cat-7. A mixture of **HEG-CC** (11.0 mg, 0.03 mmol) and CB[6] (62.0 mg, 0.06 mmol) in 0.2 M HCl (16 mL) was heated to 70 °C for 2 h until a clear solution was obtained. A solution of **DN-N3**[2] (20.0 mg, 0.03 mmol, 16 mL) in the same solvent was added over 2 h and the reaction mixture was heated to 70 °C overnight. HPLC analysis showed that ca. 89% of the materials in the product mixture corresponded to **Cat-7**. Saturated aq. NH_4PF_6 (5 mL) was added and the pale yellow precipitate was collected by filtration, washed with water (3 × 5 mL) and dried in vacuum. Isolated yield = 60 mg, 65%. ^{1}H NMR (500 MHz, CD_3CN/D_2O v/v 10:1, 298 K) δ = 7.78 (m, 2H), 7.38 (m, 2H), 6.93 (m , 2H), 6.33 (s, 2H), 5.59-5.56 (m, 24H), 5.26-5.25 (m, 24H), 4.25 (m, 4H), 4.00-3.90 (m, 32H), 3.73-3.72 (m, 8H), 3.70-3.69 (m, 8H), 3.66-3.59 (m, 16H), 3.57-3.43 (m, 20H), 3.39 (m, 4H) ^{13}C{^{1}H} NMR (100 MHz, CD_3CN/D_2O v/v 10:1, 298 K) δ = 155.8, 154.4, 138.9, 125.9, 120.1, 114.3, 106.4, 70.4, 70.0, 70.0, 69.9, 69.8, 69.4, 68.0, 65.9, 65.4, 51.4, 51.2, 48.4, 47.4, 46.0, 42.4, 25.3, 20.5, 13.6. ESI-MS: 751.1 $[M+4H]^{4+}$ 1001.1 $[M+3H]^{3+}$.

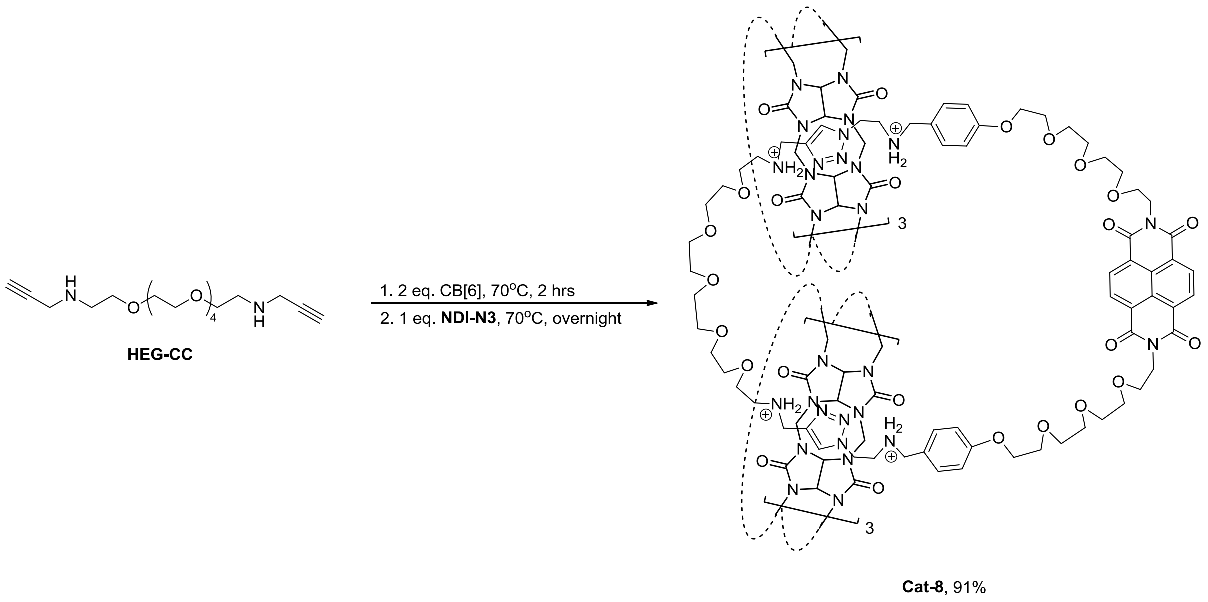

Cat-8. A mixture of **HEG-CC** (11.0 mg, 0.03 mmol) and CB[6] (62.0 mg, 0.06 mmol) in 0.2 M HCl (16 mL) was heated to 70 °C for 2 h until a clear solution was obtained. A solution of **NDI-N3** (29.0 mg, 0.03 mmol, 16 mL) in the same solvent was added over 2 h and the reaction mixture was heated to 70 °C overnight. HPLC analysis showed that ca. 91% of the materials in the product mixture corresponded to **Cat-8**. Saturated aq. NH_4PF_6 (5 mL) was added and the pale yellow precipitate was collected by filtration, washed with water (3 × 5 mL) and dried in vacuum. Isolated yield = 66 mg, 64%. ^{1}H NMR (500 MHz, CD_3CN/D_2O v/v 10:1, 298 K) δ = 8.63 (s, 4H), 7.56-7.53 (m, 4H), 6.87-6.85 (m , 4H), 6.39 (s, 2H), 5.66-5.59 (m, 24H), 5.33 (s, 24H), 4.40-4.27 (m, 4H), 4.28-4.27 (m, 4H), 4.09-4.02 (m, 40H), 3.77-3.72 (m, 8H), 3.68-3.52 (m, 24H), 3.49-3.45 (m, 16H) $^{13}C\{^{1}H\}$ NMR (125 MHz, CD_3CN/D_2O v/v 10:1, 298 K) δ = 163.5, 159.0, 156.1, 155.8, 138.9, 131.6, 130.9, 126.5, 124.6, 114.8, 78.2, 70.0, 69.8, 69.8, 69.7, 69.6, 69.0, 67.2, 65.3, 65.1, 51.2, 51.1, 50.6, 48.2, 48.1, 45.6, 45.2, 42.3, 39.5, 36.1. ESI-MS: 830.3 $[M+4H]^{4+}$ 1106.8 $[M+3H]^{3+}$.

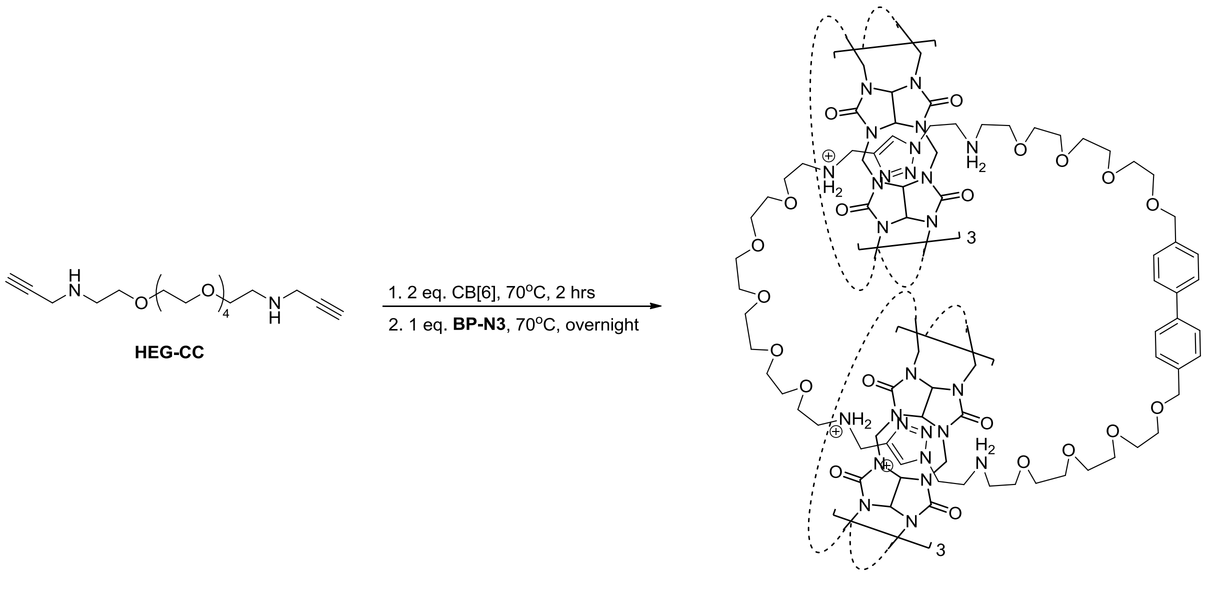

Cat-9, 92%

Cat-9. A mixture of **HEG-CC** (11.0 mg, 0.03 mmol) and CB[6] (62.0 mg, 0.06 mmol) in 0.2 M HCl (16 mL) was heated to 70 °C for 2 h until a clear solution was obtained. A solution of **BP-N3** (21.0 mg, 0.03 mmol, 16 mL) in the same solvent was added over 2 h and the reaction mixture was heated to 70 °C overnight. HPLC analysis showed that ca. 92% of the materials in the product mixture corresponded to **Cat-9**. Saturated aq. NH_4PF_6 (5 mL) was added and the pale yellow precipitate was collected by filtration, washed with water (3 × 5 mL) and dried in vacuum. Isolated yield = 61 mg, 67%. ^1H NMR (500 MHz, CD_3CN/D_2O v/v 10:1, 298 K) δ = 7.63 (m, 4H), 7.41 (m, 4H), 6.37 (s, 2H), 5.63-5.59 (m, 24H), 5.30 (s, 24H), 4.54 (s, 4H), 4.10-4.00 (32H), 3.94-3.92 (m, 4H), 3.76-3.75 (m, 4H), 3.71-3.70 (m, 4H), 3.67-3.66 (m, 4H), 3.61-3.58 (m, 32H), 3.53 (m, 4H), 3.43 (m, 4H) $^{13}C\{^1H\}$ NMR (125 MHz, CD_3CN/D_2O v/v 10:1, 298 K) δ = 164.1, 156.1, 128.6, 126.8, 120.0, 72.2, 72.2, 69.9, 69.3, 69.2, 69.1, 65.7, 65.3, 65.1, 51.1, 48.3, 47.3, 47.1, 46.7, 46.0, 45.8, 45.2, 42.2, 36.4, 31.0. ESI-MS: 764.5 $[M+4H]^{4+}$ 1018.7 $[M+3H]^{3+}$.

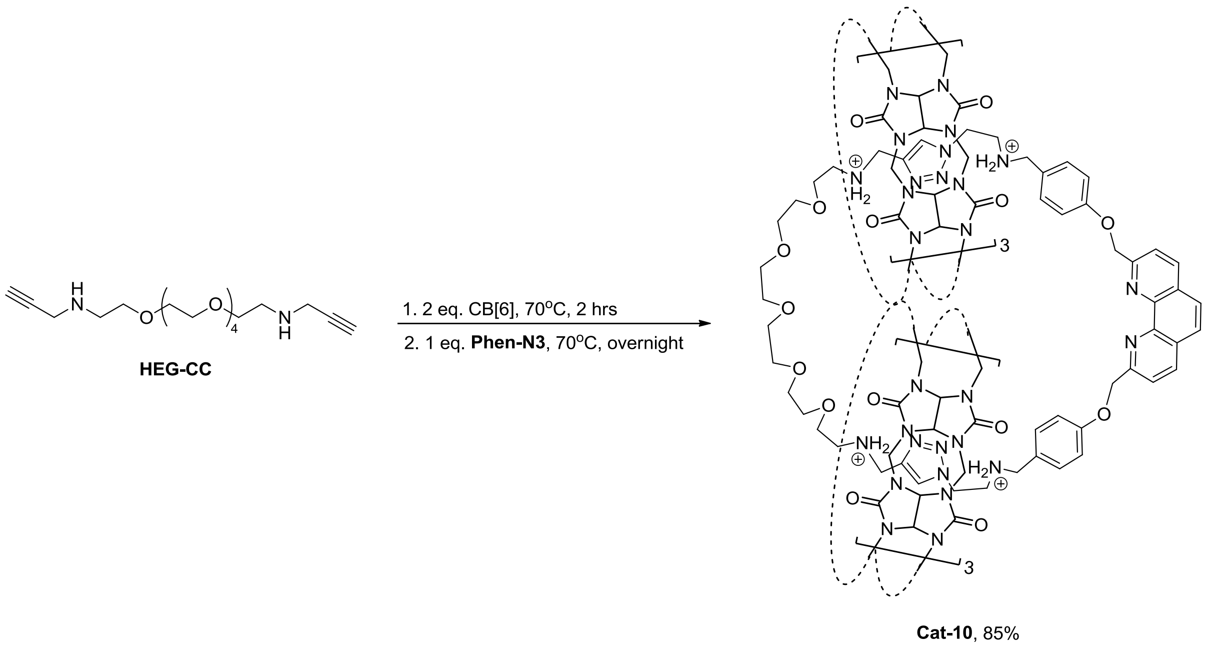

Cat-10. A mixture of **HEG-CC** (11.0 mg, 0.03 mmol) and CB[6] (62.0 mg, 0.06 mmol) in 0.2 M HCl (16 mL) was heated to 70 °C for 2 h until a clear solution was obtained. A solution of **Phen-N3** (16.0 mg, 0.03 mmol, 16 mL) in the same solvent was added over 2 h and the reaction mixture was heated to 70 °C overnight. HPLC analysis showed that ca. 85% of the materials in the product mixture corresponded to **Cat-10**. Saturated aq. NH_4PF_6 (5 mL) was added and the white precipitate was collected by filtration, washed with water (3 × 5 mL) and dried in vacuum. Isolated yield = 54 mg, 59%. ^{1}H NMR (500 MHz, CD_3CN/D_2O v/v 10:1, 298 K) δ = 8.49 (m, 2H), 7.94 (m, 4H), 7.64 (m, 4H), 7.17 (m, 4H), 6.39 (s, 2H), 5.63-5.58 (m, 24H), 5.32 (s, 24H), 4.45 (m, 4H), 4.12-4.07 (m, 24H), 3.78 (m, 4H), 3.68-3.60 (m, 24H), 3.45-3.43 (m, 4H) ^{13}C{^{1}H} NMR (125 MHz, CD_3CN/D_2O v/v 10:1, 298 K) δ = 158.7, 156.1, 155.8, 155.8, 155.7, 138.9, 138.7, 131.8, 128.6, 126.8, 125.3, 115.3, 78.0, 69.9, 69.7, 65.3, 65.1, 51.2, 51.1, 50.6, 46.1, 45.6, 45.2, 42.3, 36.1. ESI-MS: 735.5 $[M+4H]^{4+}$ 980.3 $[M+3H]^{3+}$.

C. [4]Catenane synthesis

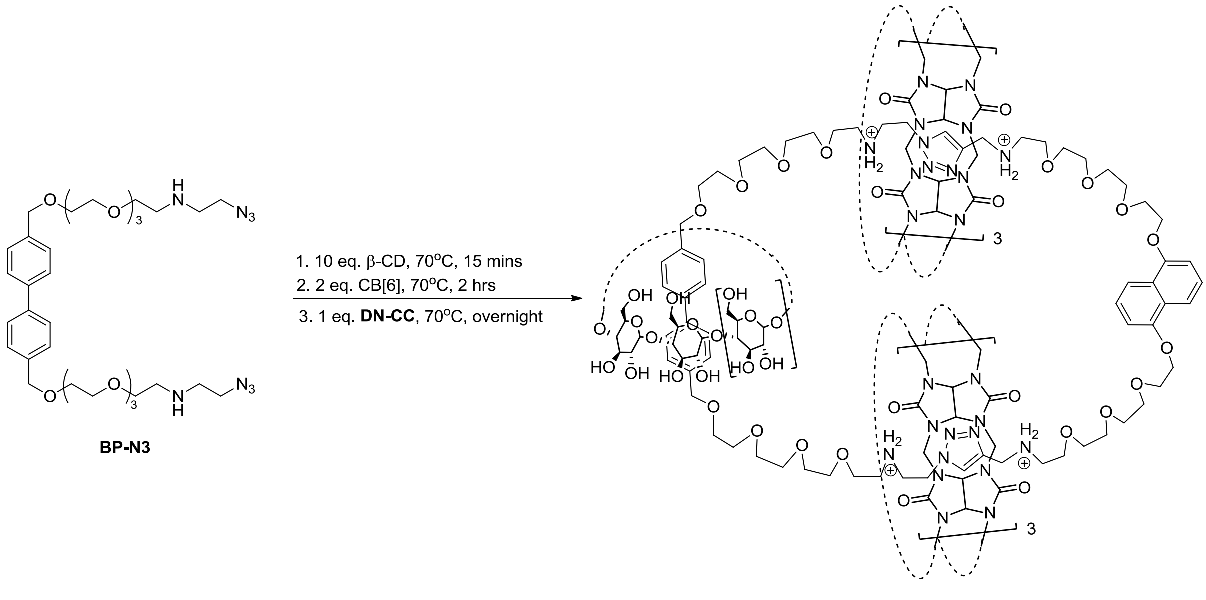

Cat-11, 50%

Cat-11. A mixture of **BP-N3** (21.0 mg, 0.03 mmol) and β-CD (340 mg, 0.3 mmol) in 0.2 M HCl (25 mL) was heated to 70 °C for 15 min. CB[6] (65.0 mg, 0.06 mmol) was then added and the reaction mixture was heated to 70 °C for 2 h. A solution of **DN-CC** (19.0 mg, 0.03 mmol, 25 mL) in the same solvent was added over 2 h and heating was continued overnight. HPLC analysis showed that ca. 30% and 60% of the materials in the product mixture corresponded to **Cat-3** and **Cat-11**, respectively. **Cat-11** was isolated from the crude mixture by preparative HPLC. The reaction mixture was concentrated to 4 mL and 0.4 mL of this solution was injected onto the preparative column. Isolated yield = 6 mg, 45%. ^{1}H NMR (500 MHz, D_2O, 298 K) δ 7.92 (d, J = 8.5 Hz, 2H), 7.61 (s, 4H), 7.53 (d, J = 8.0 Hz, 2H), 7.48 (s, 4H), 7.12 (d, J = 7.7 Hz, 2H), 6.44 (s, 2H), 5.71 (dd, J = 15.6, 4.8 Hz, 12H), 5.65-5.56 (m, 12H), 5.38 (d, J = 7.0 Hz, 24H), 5.02 (s, 7H), 4.68 (s, 4H), 4.39 (s, 4H), 4.24-4.20 (m, 8H), 4.19-4.11 (m, 24H), 4.06-4.02 (m, 8H), 3.88-3.84 (m, 14H), 3.79-3.69 (m, 52H), 3.64-3.56 (m, 24H), 3.52 (s, 8H). ^{13}C{^{1}H} NMR (100 MHz, D_2O, 298 K) δ = 156.2, 156.1, 153.9, 139.8, 138.8, 137.2, 128.6, 126.4, 126.2, 119.8, 110.15, 102.0, 80.7, 73.3, 72.1, 71.9, 71.9, 70.1, 69.8, 69.7, 69.6, 69.5, 69.2, 68.4, 68.1, 65.7, 65.20, 59.6, 53.1, 51.4, 51.2, 48.2, 47.2, 45.7, 45.1, 42.3. ESI-MS: 1105.5 $[M+4H]^{4+}$ 1473.5 $[M+3H]^{3+}$

2. HPLC analysis

HPLC analyses were carried out using a Waters-Alliance e2695 system coupled to a 2489 UV–vis detector. HPLC grade H_2O (Scharlau), MeCN (Arkonic Scientific) and formic acid (Merck) were used as received. C18 SunFire preparative columns (5 μm, 10 × 250 mm or 10 μm, 4.6 × 250 mm) were used with gradient elution described below. UV–vis absorbance was monitored at 380 nm for **Cat-2**, **Cat-4** and **Cat-8**, 260 nm for **Cat-3**, **Cat-5, Cat-9** and **Cat-11**, 310 nm **for Cat-1** and **Cat-7** and 280 nm for **Cat-6** and **Cat-10**, respectively.

Method 1 (flow rate = 0.6 mL/min)

time/min	H_2O (with 0.05% formic acid)	MeCN (with 0.05% formic acid)
0	95%	5%
5	80%	20%
15	70%	30%
17	50%	50%
18	0%	100%
20	0%	100%

Method 2 (flow rate = 3 mL/min)

time/min	H_2O (with 0.05% formic acid)	MeCN (with 0.05% formic acid)
0	85%	15%
3	85%	15%
10	76%	24%
13	74%	26%
18	65%	35%
22	35%	65%
25	0%	100%
30	0%	100%

Method 3 (flow rate = 3 mL/min)

Time/min	H_2O (with 0.05% formic acid)	MeCN (with 0.05% formic acid)
0	85%	15%
3	85%	15%
10	76%	24%
13	74%	26%
18	65%	35%
19	0%	100%
20	0%	100%

Method 4 (flow rate = 3 mL/min)

Time/min	H_2O (with 0.05% formic acid)	MeCN (with 0.05% formic acid)
0	87%	13%
2	87%	13%
12	65%	35%
15	20%	80%
20	20%	80%

Method 5 (flow rate = 3 mL/min)

Time/min	H_2O (with 0.05% formic acid)	MeCN (with 0.05% formic acid)
0	90%	10%
1	90%	10%
13	70%	30%
15	65%	35%
16	0%	100%
20	0%	100%

Method 6 (flow rate = 3 mL/min)

Time/min	H_2O (with 0.05% formic acid)	MeCN (with 0.05% formic acid)
0	95%	5%
1	95%	5%
8	80%	20%
9	0%	100%
10	0%	100%

Method 7 (flow rate = 3 mL/min)

Time/min	H_2O (with 0.05% formic acid)	MeCN (with 0.05% formic acid)
0	95%	5%
3	95%	5%
13	70%	30%
15	65%	35%
16	0%	100%
20	0%	100%

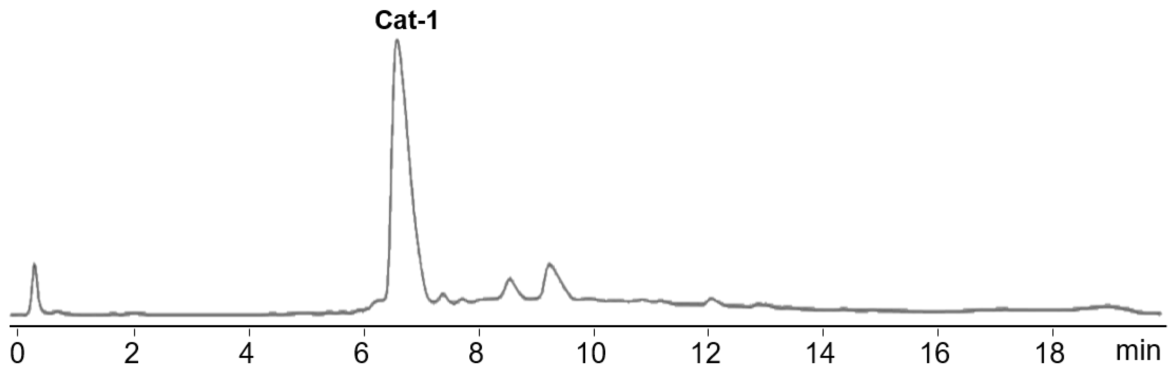

Figure S1. HPLC chromatogram of the crude product mixture of **Cat-1**. Separation was achieved using *Method 1*.

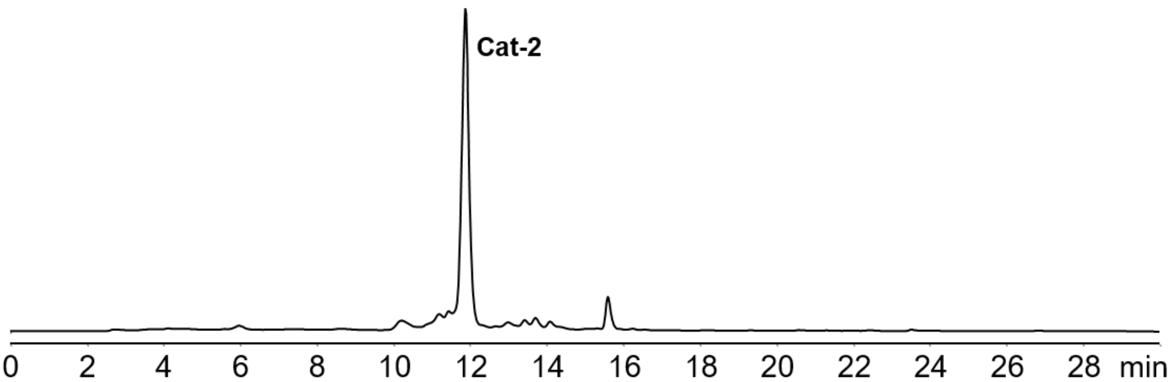

Figure S2. HPLC chromatogram of the crude product mixture of **Cat-2**. Separation was achieved using *Method 2*.

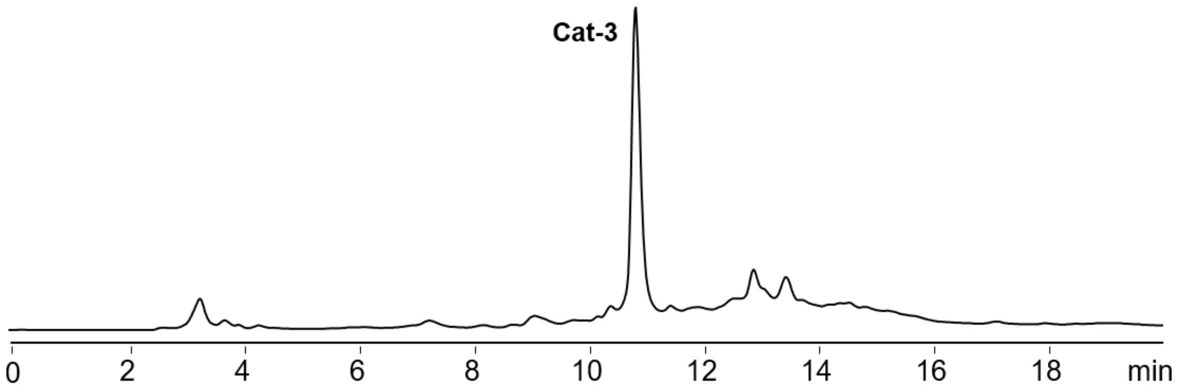

Figure S3. HPLC chromatogram of the crude product mixture of **Cat-3**. Separation was achieved using *Method 3*.

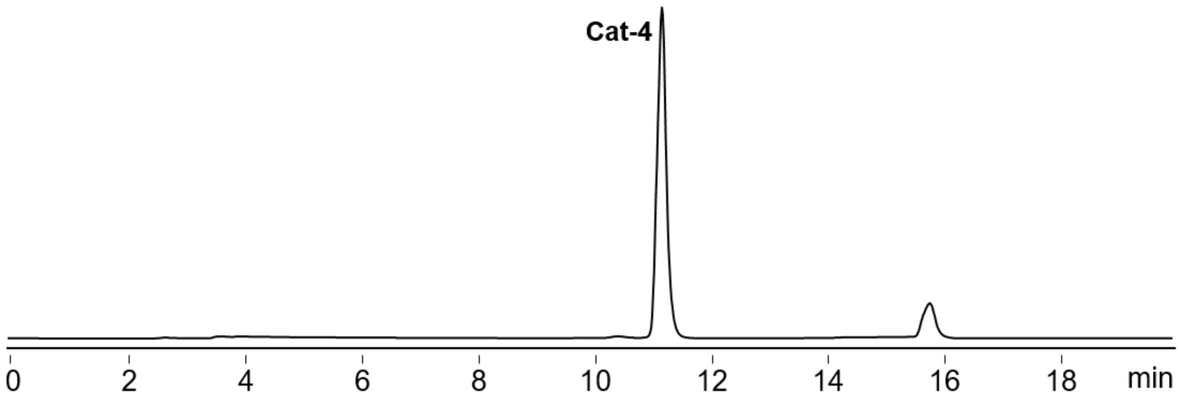

Figure S4. HPLC chromatogram of the crude product mixture of **Cat-4**. Separation was achieved using *Method 3*.

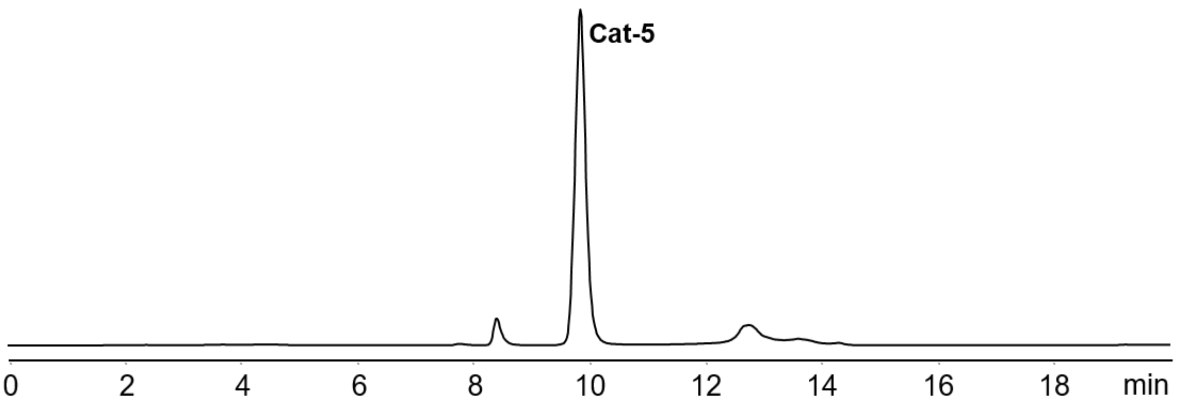

Figure S5. HPLC chromatogram of the crude product mixture of **Cat-5**. Separation was achieved using *Method 4*.

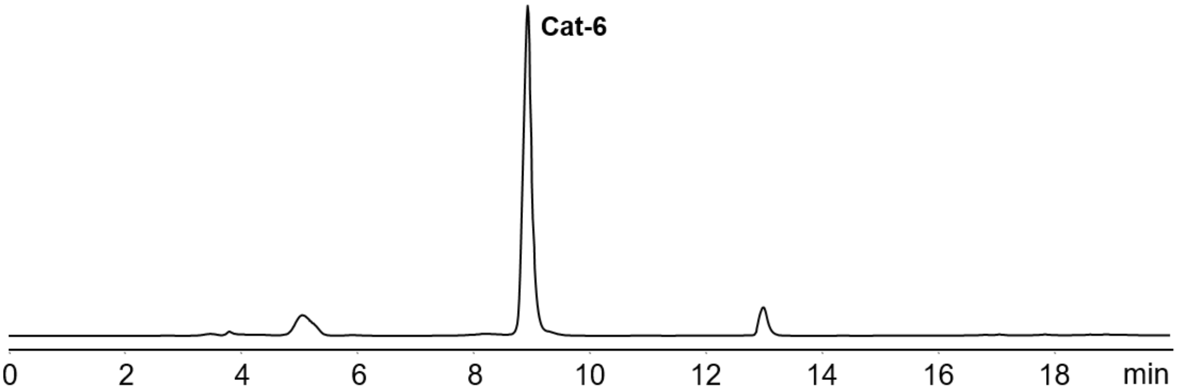

Figure S6. HPLC chromatogram of the crude product mixture of **Cat-6**. Separation was achieved using *Method 5*.

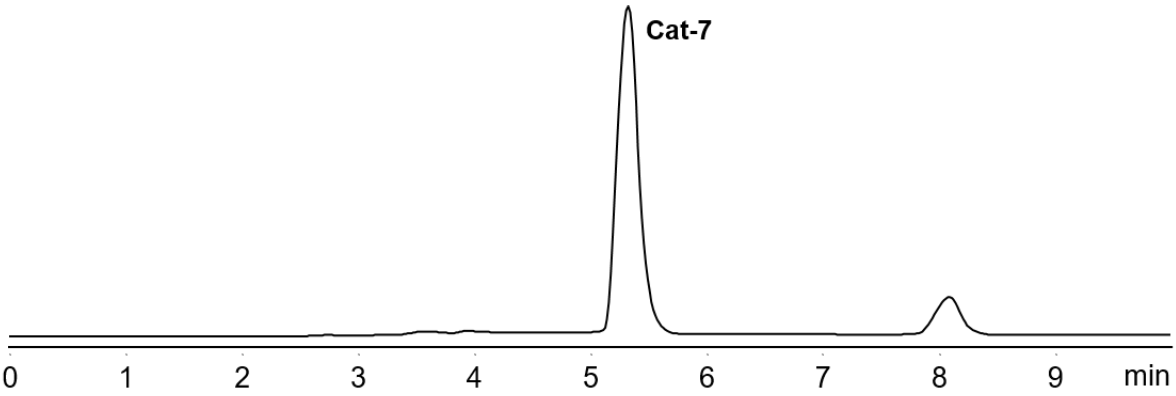

Figure S7. HPLC chromatogram of the crude product mixture of **Cat-7.** Separation was achieved using *Method 6*.

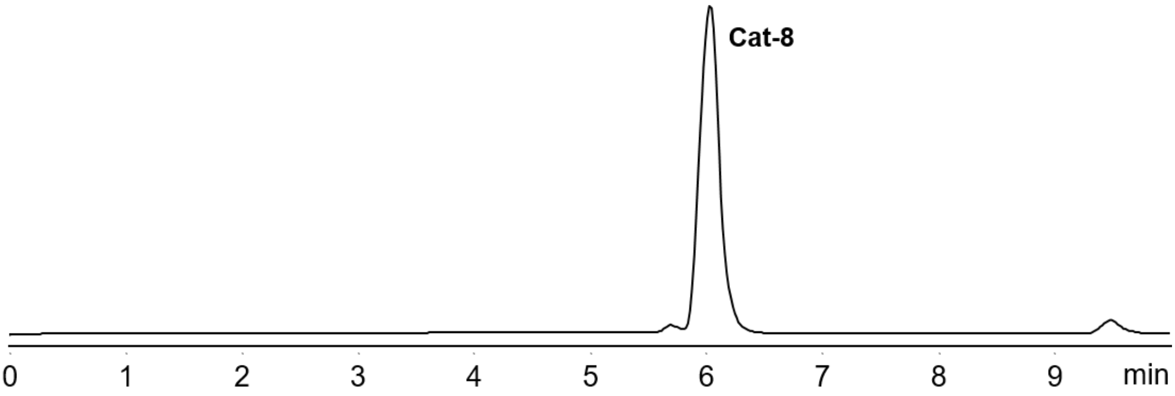

Figure S8. HPLC chromatogram of the crude product mixture of **Cat-8.** Separation was achieved using *Method 6*.

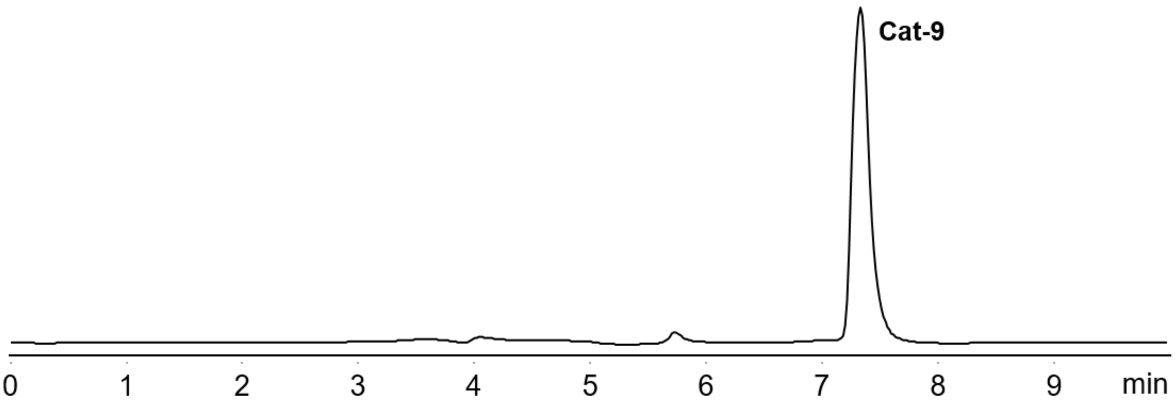

Figure S9. HPLC chromatogram of the crude product mixture of **Cat-9**. Separation was achieved using *Method 6*.

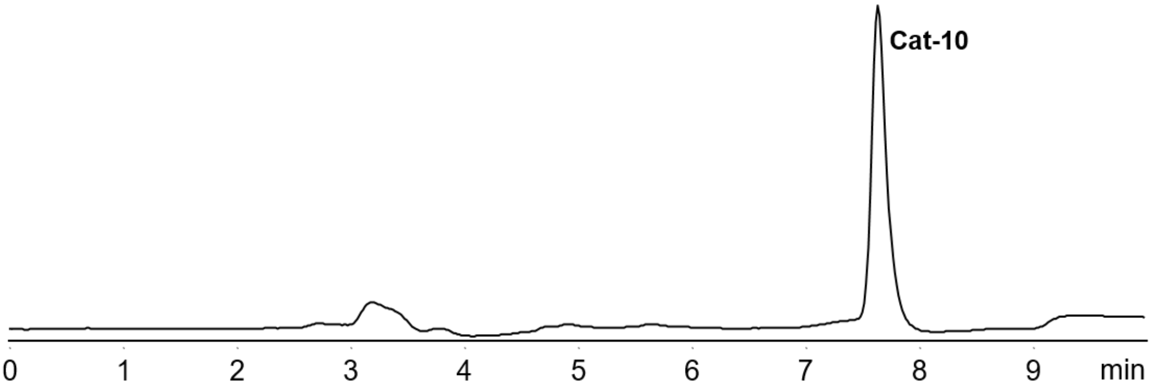

Figure S10. HPLC chromatogram of the crude product mixture of **Cat-10**. Separation was achieved using *Method 6*.

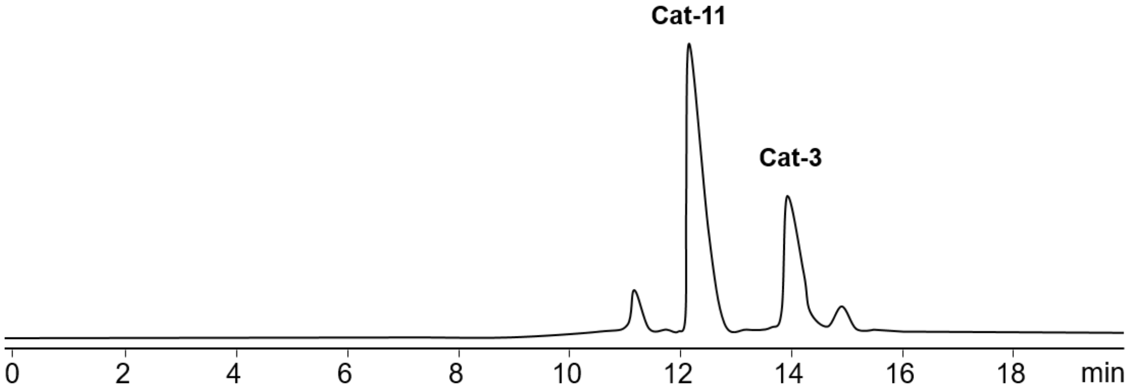

Figure S11. HPLC chromatogram of the crude product mixture of **Cat-11**. Separation was achieved using *Method 7*.

3. NMR

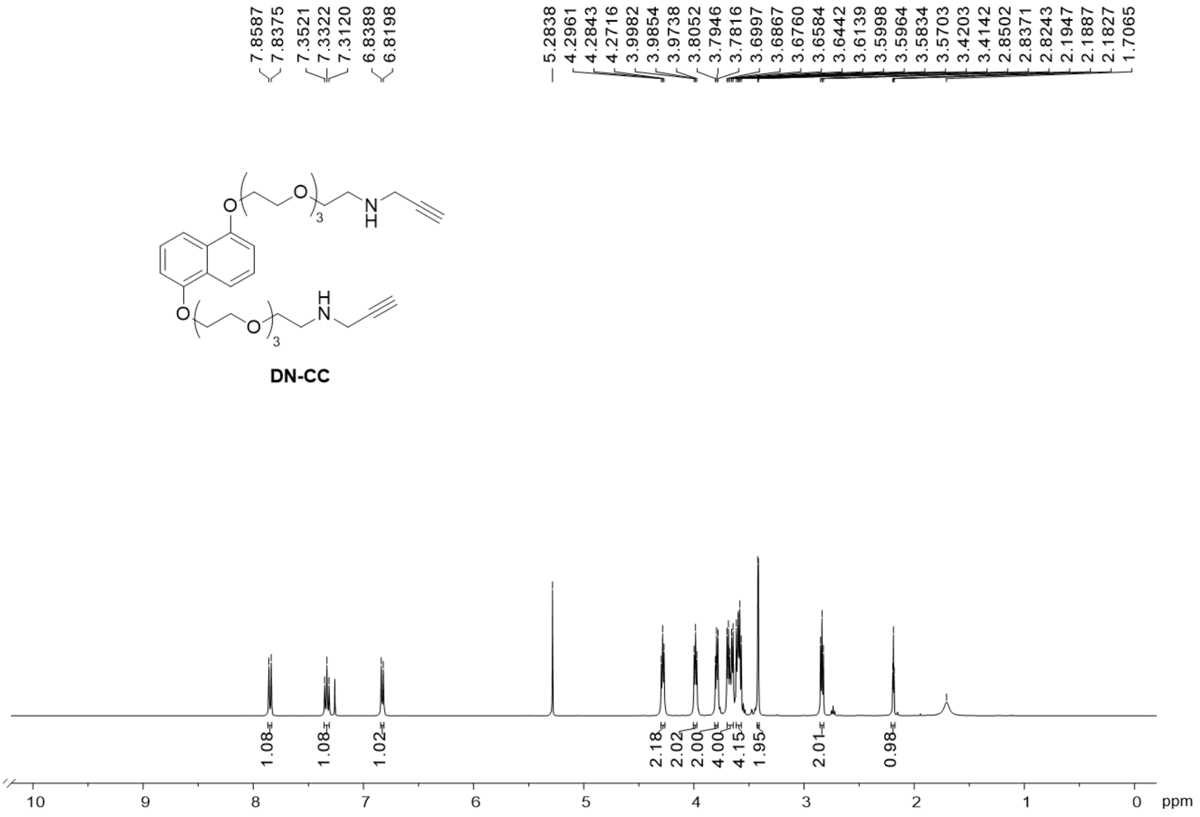

Figure S12. ^{1}H NMR (400 MHz, $CDCl_3$, 298 K) of **DN-CC**.

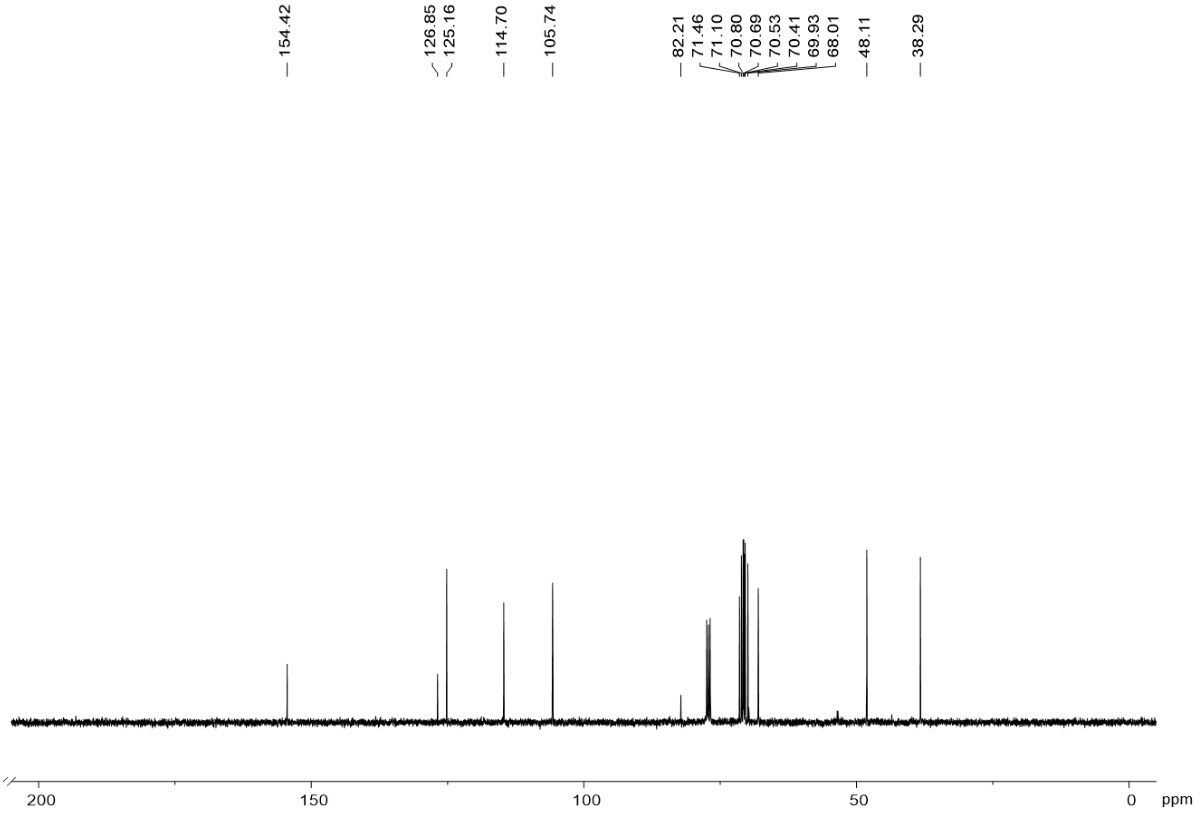

Figure S13. $^{13}C\{^{1}H\}$ NMR (100 MHz, $CDCl_3$, 298 K) of **DN-CC**.

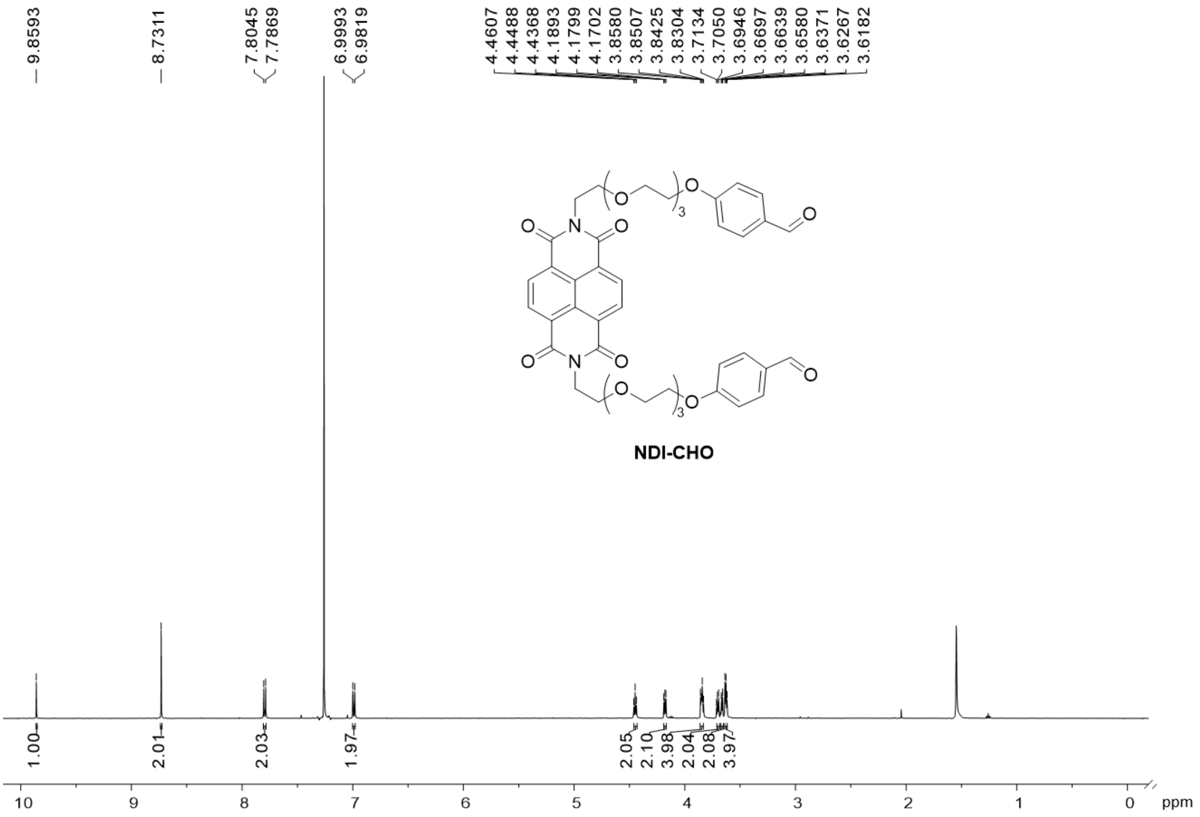

Figure S14. ^{1}H NMR (500 MHz, $CDCl_3$, 298 K) of **NDI-CHO**.

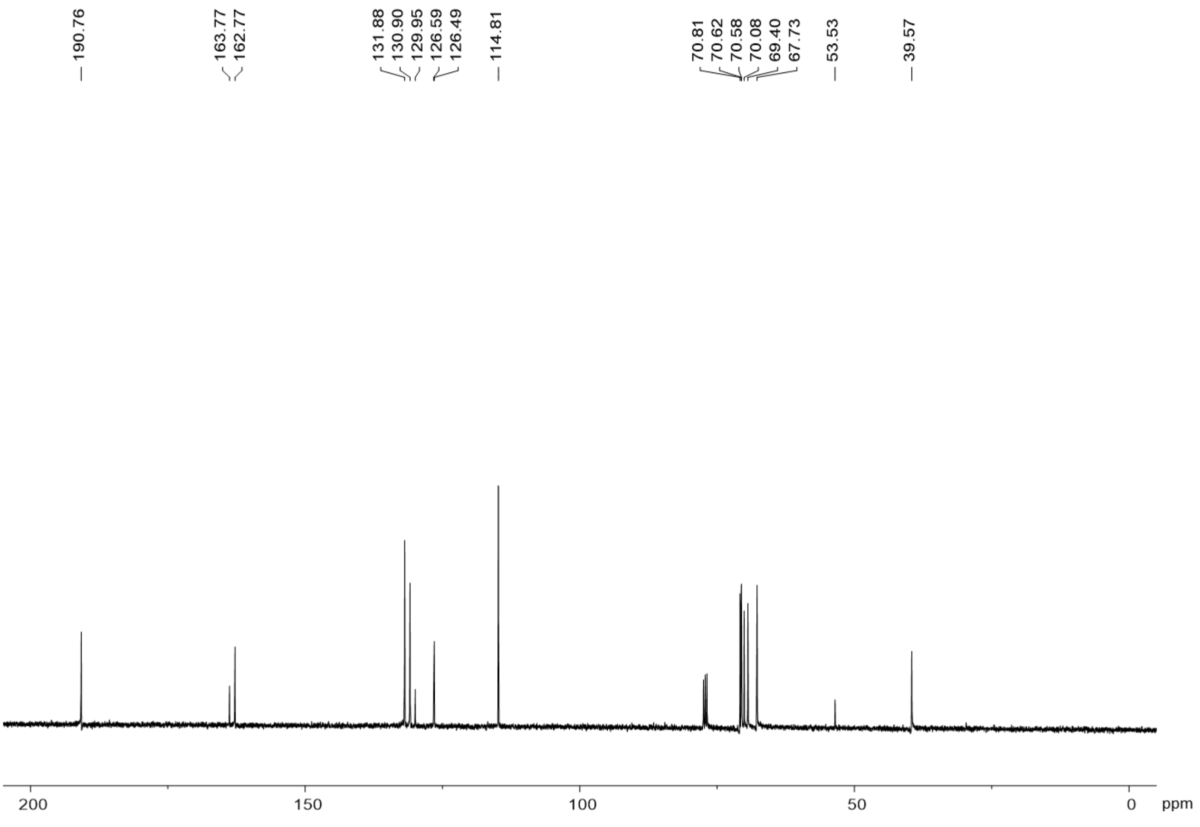

Figure S15. ^{13}C NMR (100 MHz, $CDCl_3$, 298 K) of **NDI-CHO**.

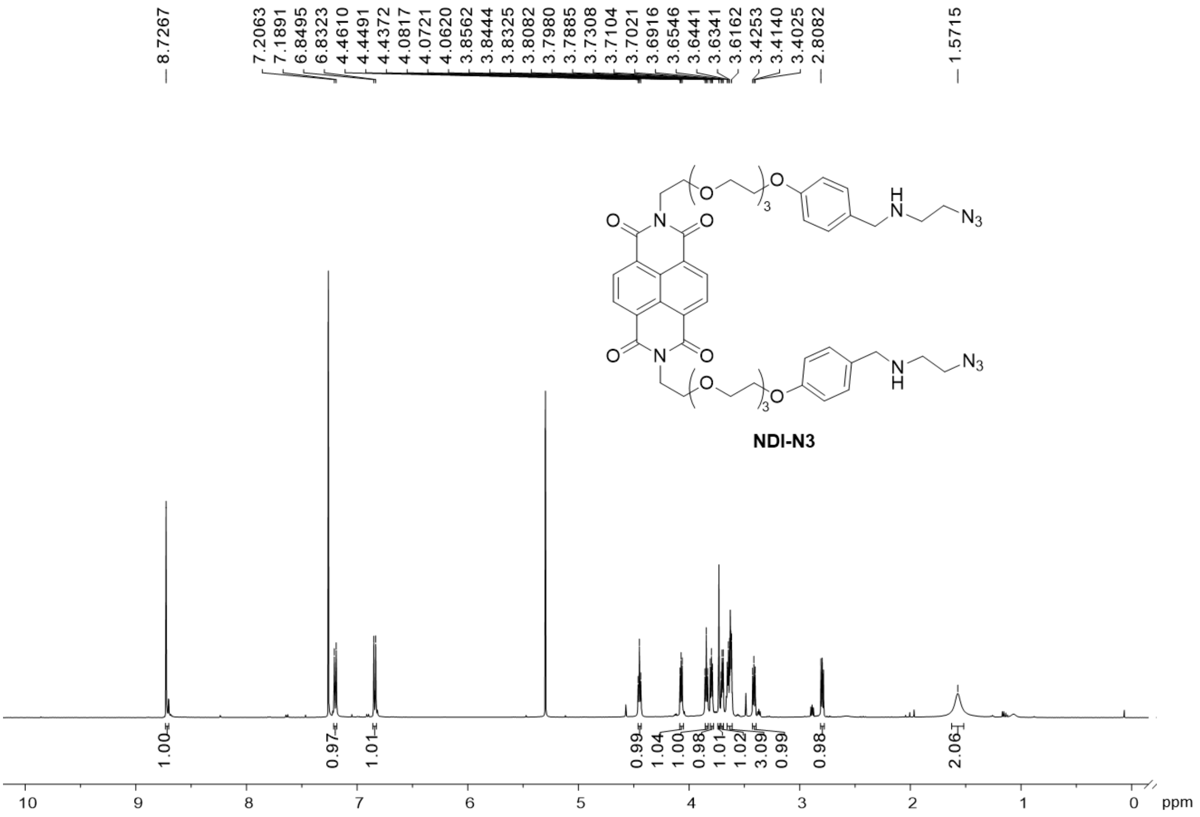

Figure S16. ^{1}H NMR (500 MHz, $CDCl_3$, 298 K) of **NDI-N3**.

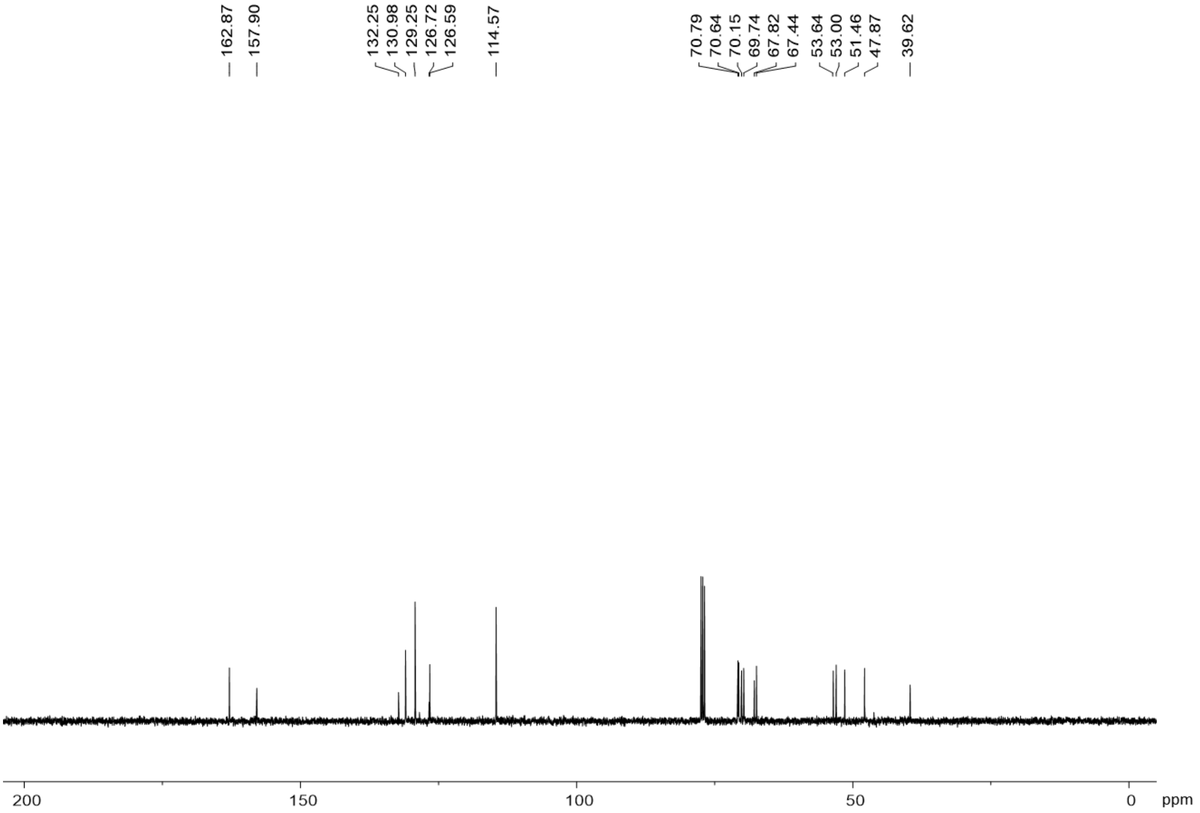

Figure S17. ^{13}C NMR (125 MHz, $CDCl_3$, 298 K) of **NDI-N3**.

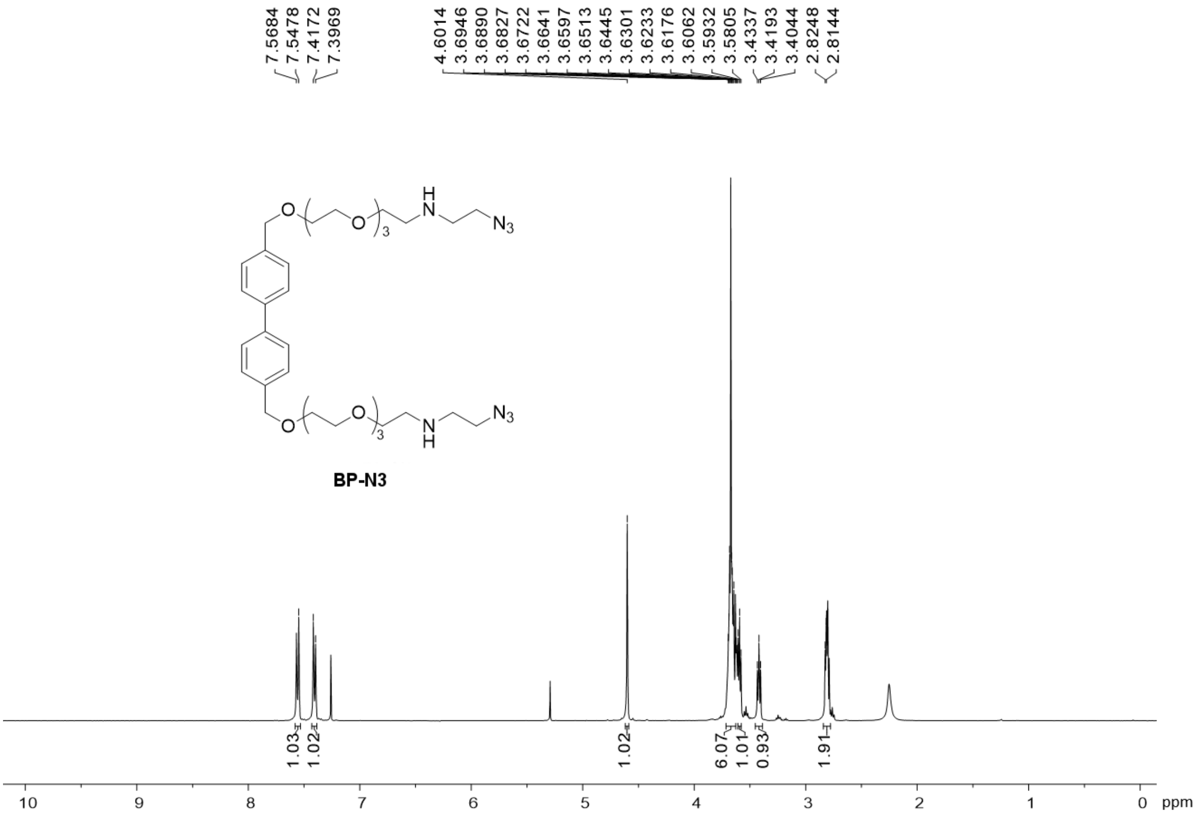

Figure S18. ^{1}H NMR (500 MHz, $CDCl_3$, 298 K) of **BP-N3**.

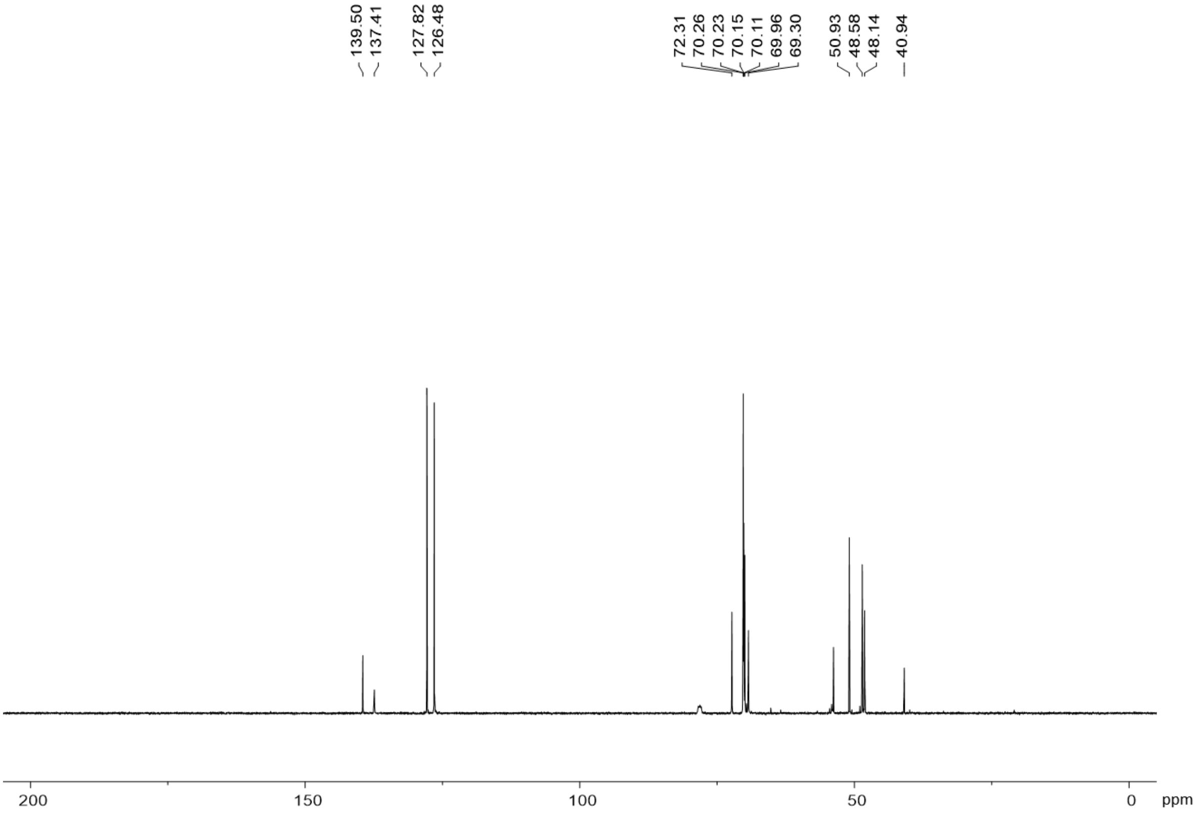

Figure S19. $^{13}C\{^{1}H\}$ NMR (125 MHz, $CDCl_3$, 298 K) of **BP-N3**.

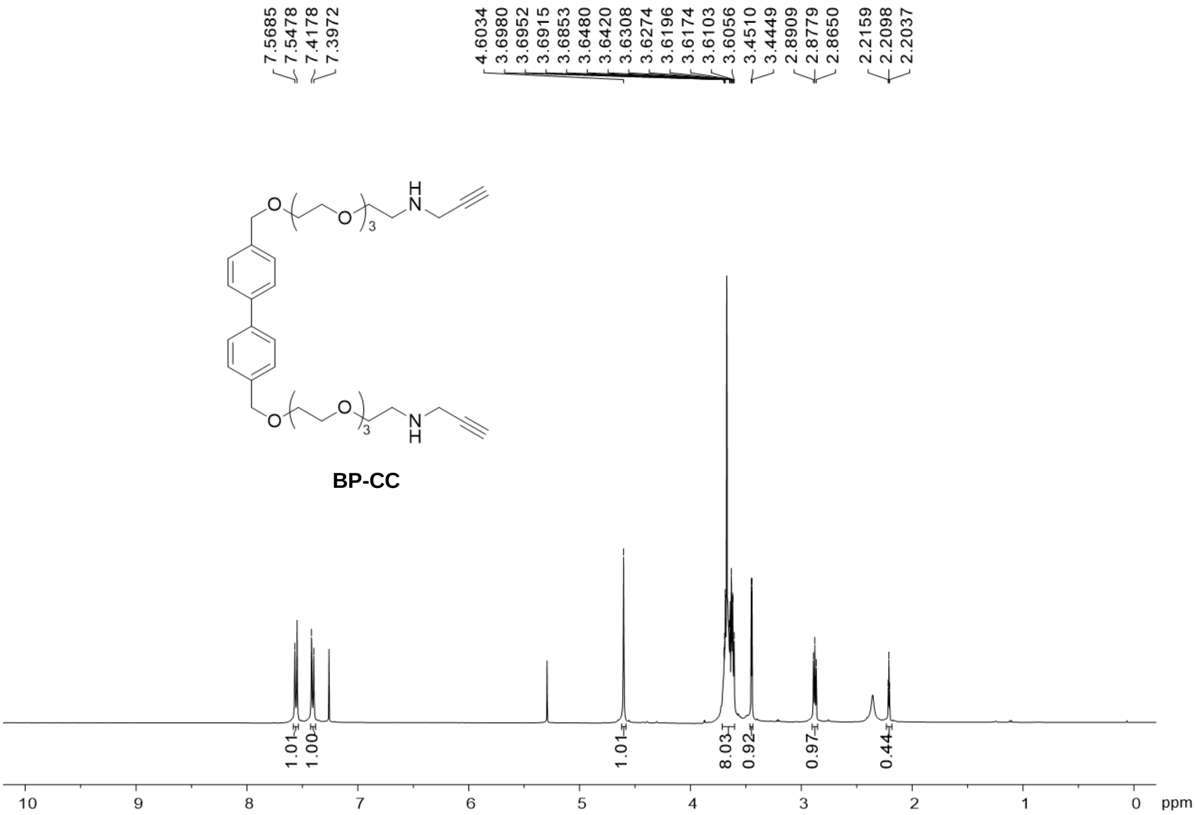

Figure S20. ^{1}H NMR (500 MHz, $CDCl_3$, 298 K) of **BP-CC**.

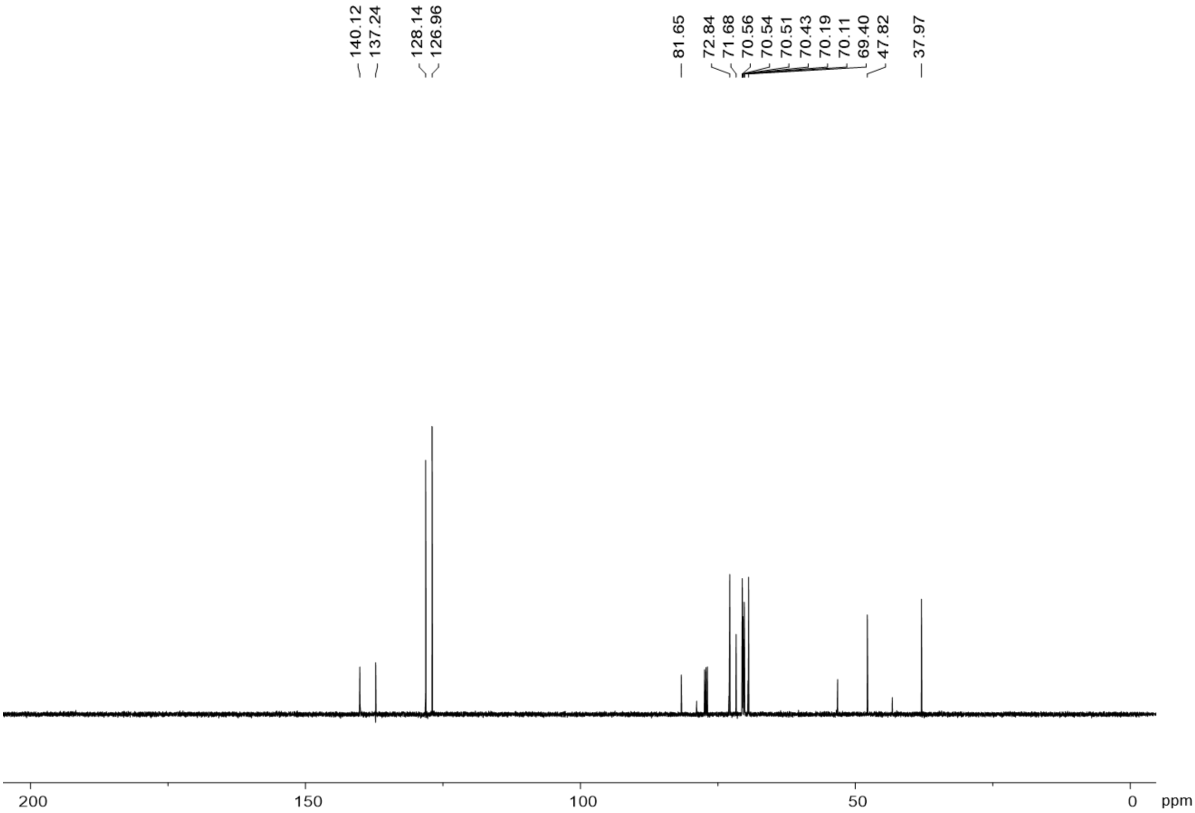

Figure S21. $^{13}C\{^{1}H\}$ NMR (125 MHz, $CDCl_3$, 298 K) of **BP-CC**.

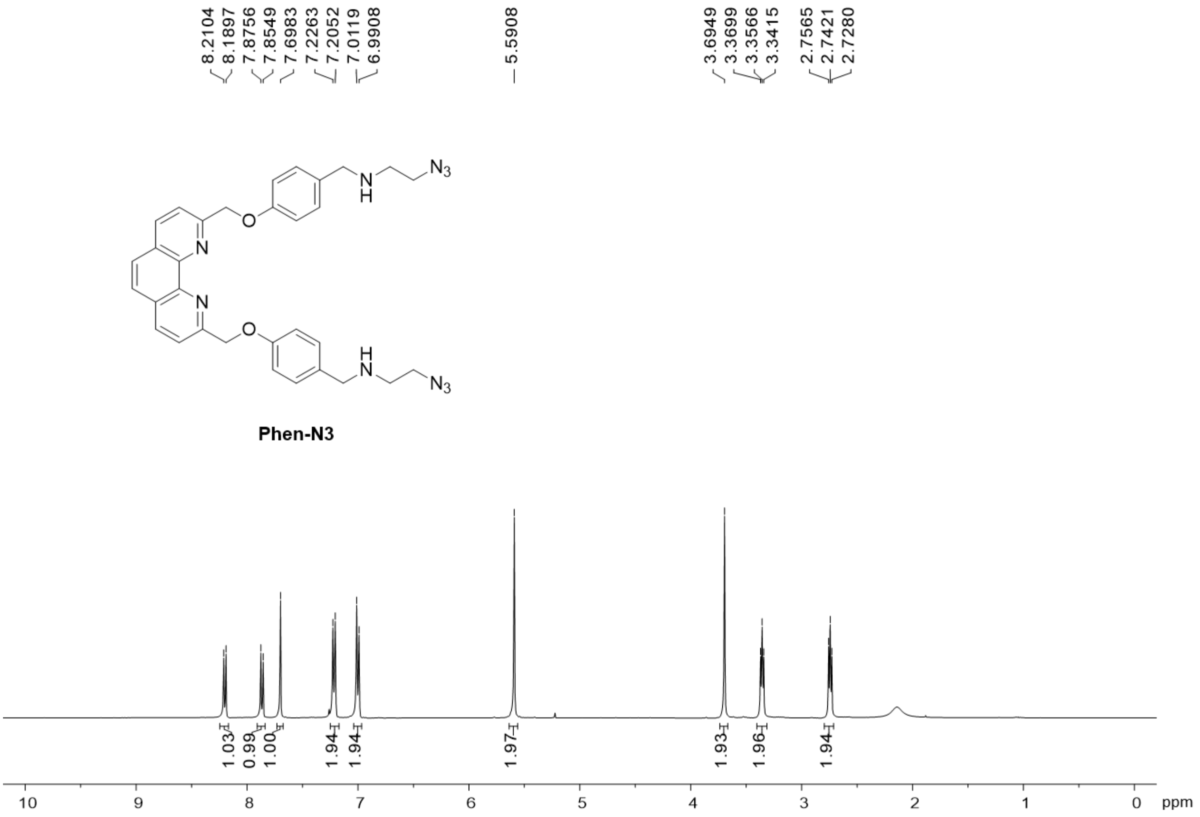

Figure S22. ^{1}H NMR (400 MHz, $CDCl_3$, 298 K) of **Phen-N3**.

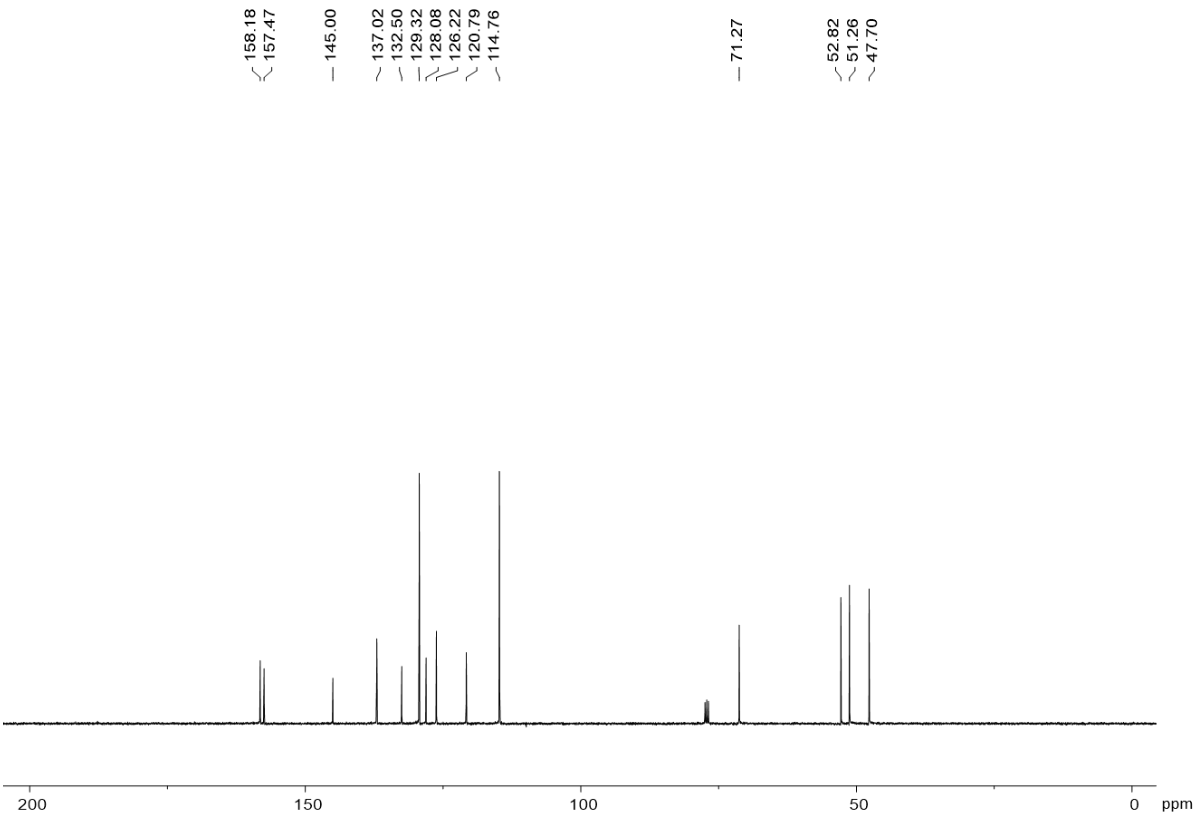

Figure S23. $^{13}C\{^{1}H\}$ NMR (100 MHz, $CDCl_3$, 298 K) of **Phen-N3**.

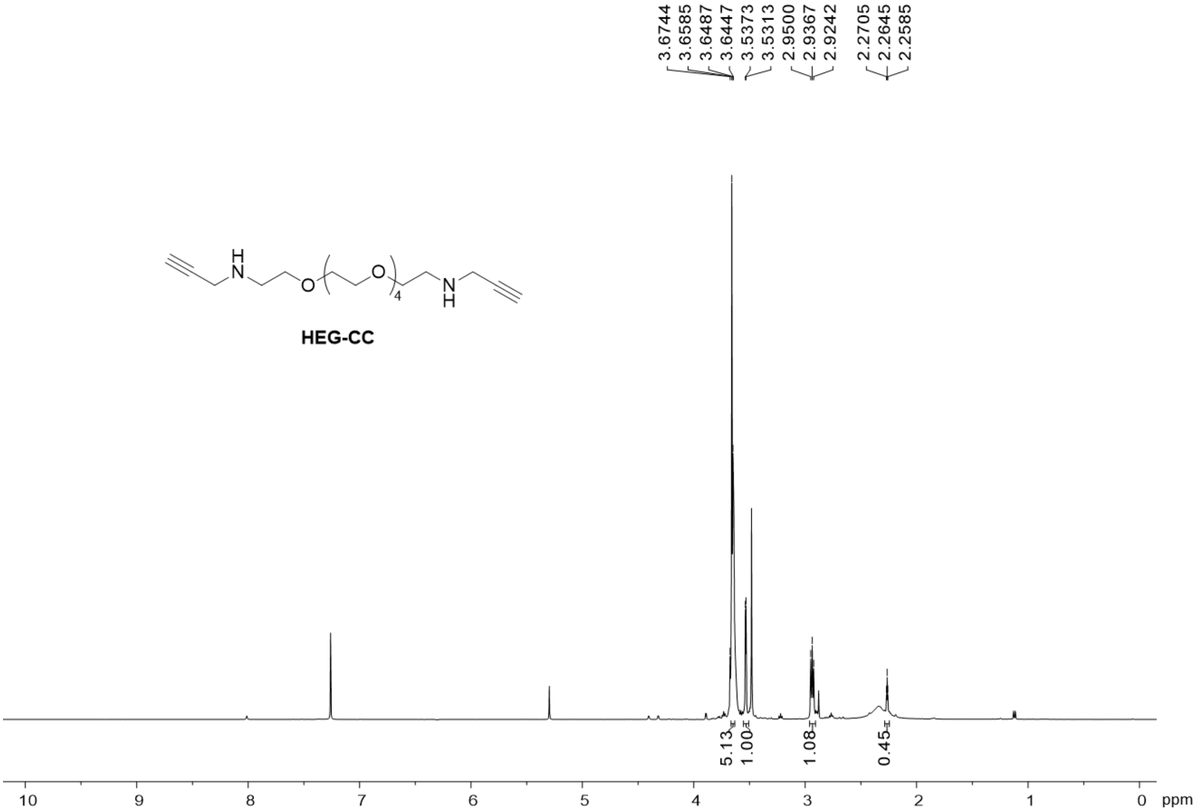

Figure S24. ^{1}H NMR (500 MHz, $CDCl_3$, 298 K) of **HEG-CC**.

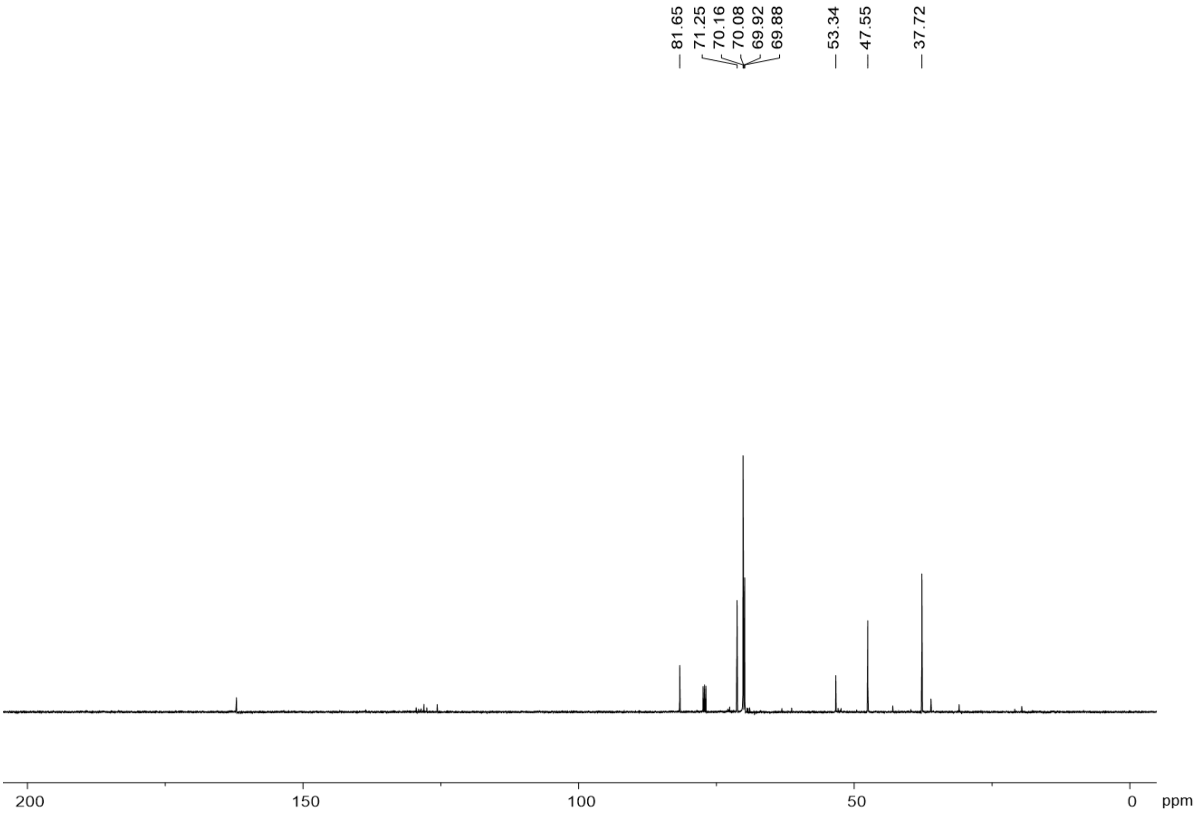

Figure S25. $^{13}C\{^{1}H\}$ NMR (125 MHz, $CDCl_3$, 298 K) of **HEG-CC**.

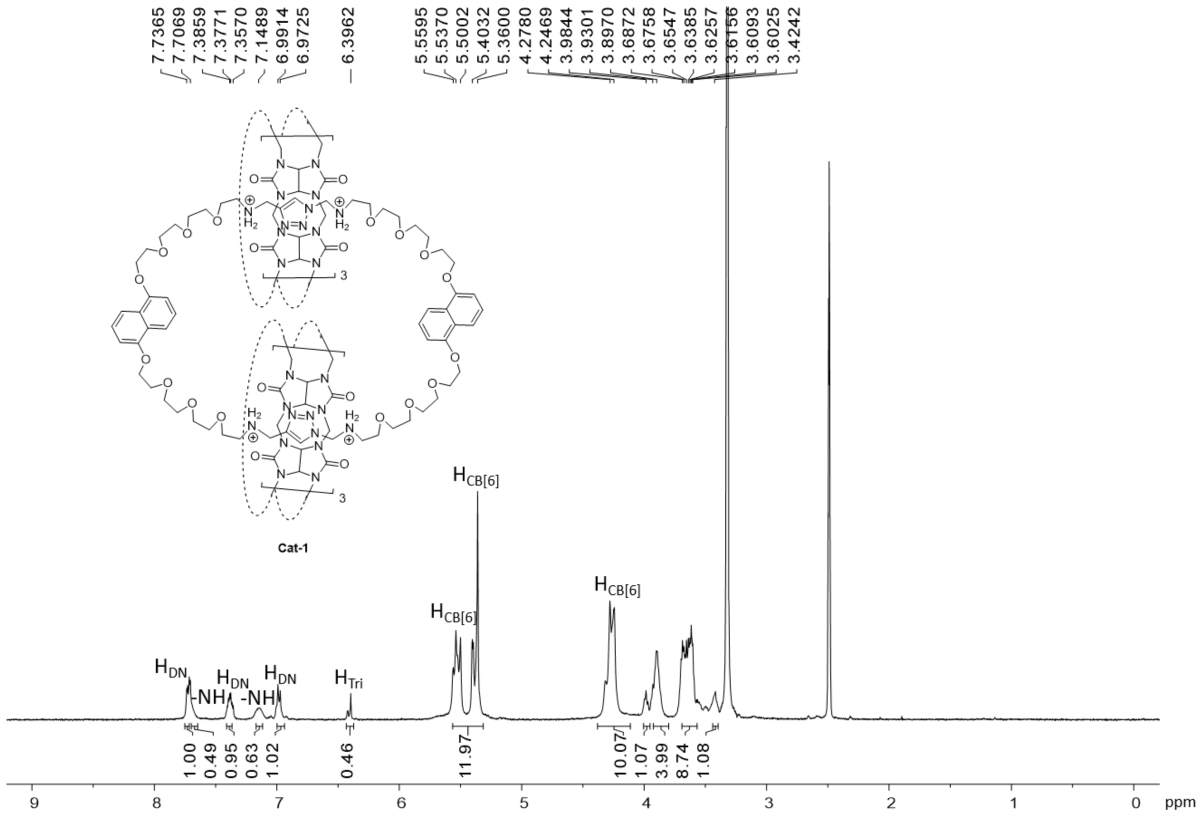

Figure S26. ^{1}H NMR (400 MHz, $(CD_3)_2SO$, 298 K) of **Cat-1**.

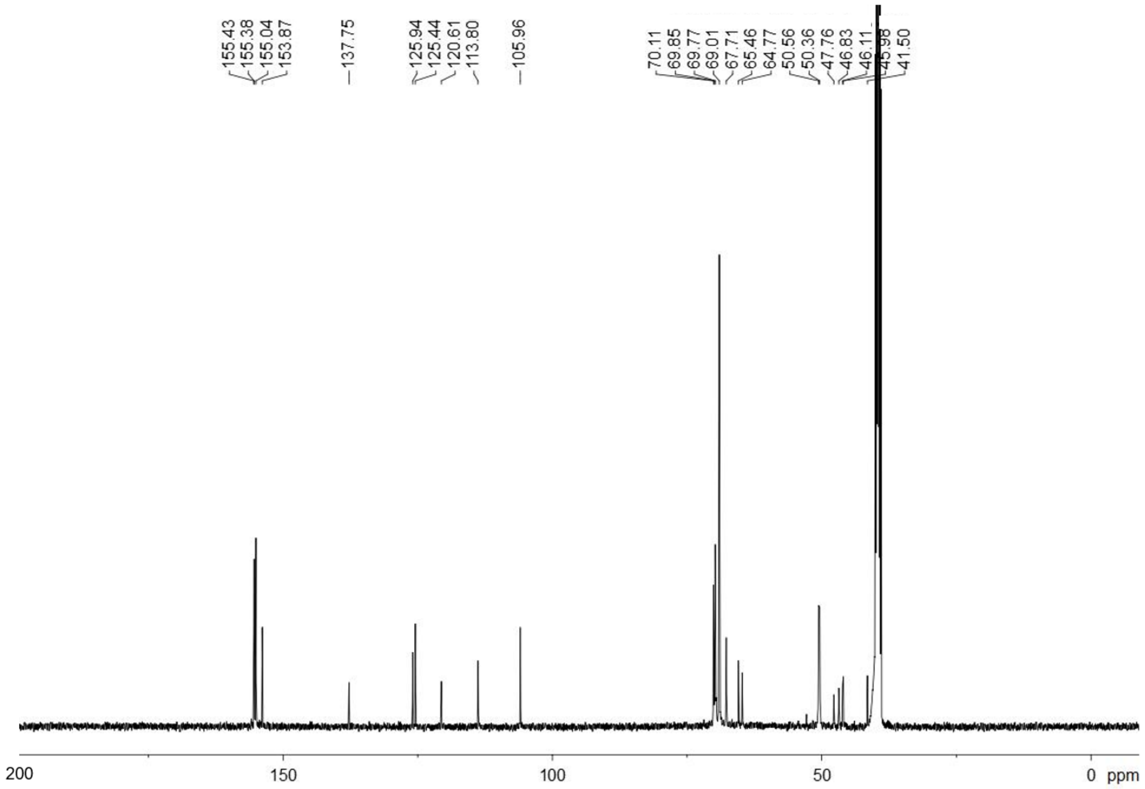

Figure S27. $^{13}C\{^{1}H\}$ NMR (100 MHz, $(CD_3)_2SO$, 298 K) of **Cat-1**.

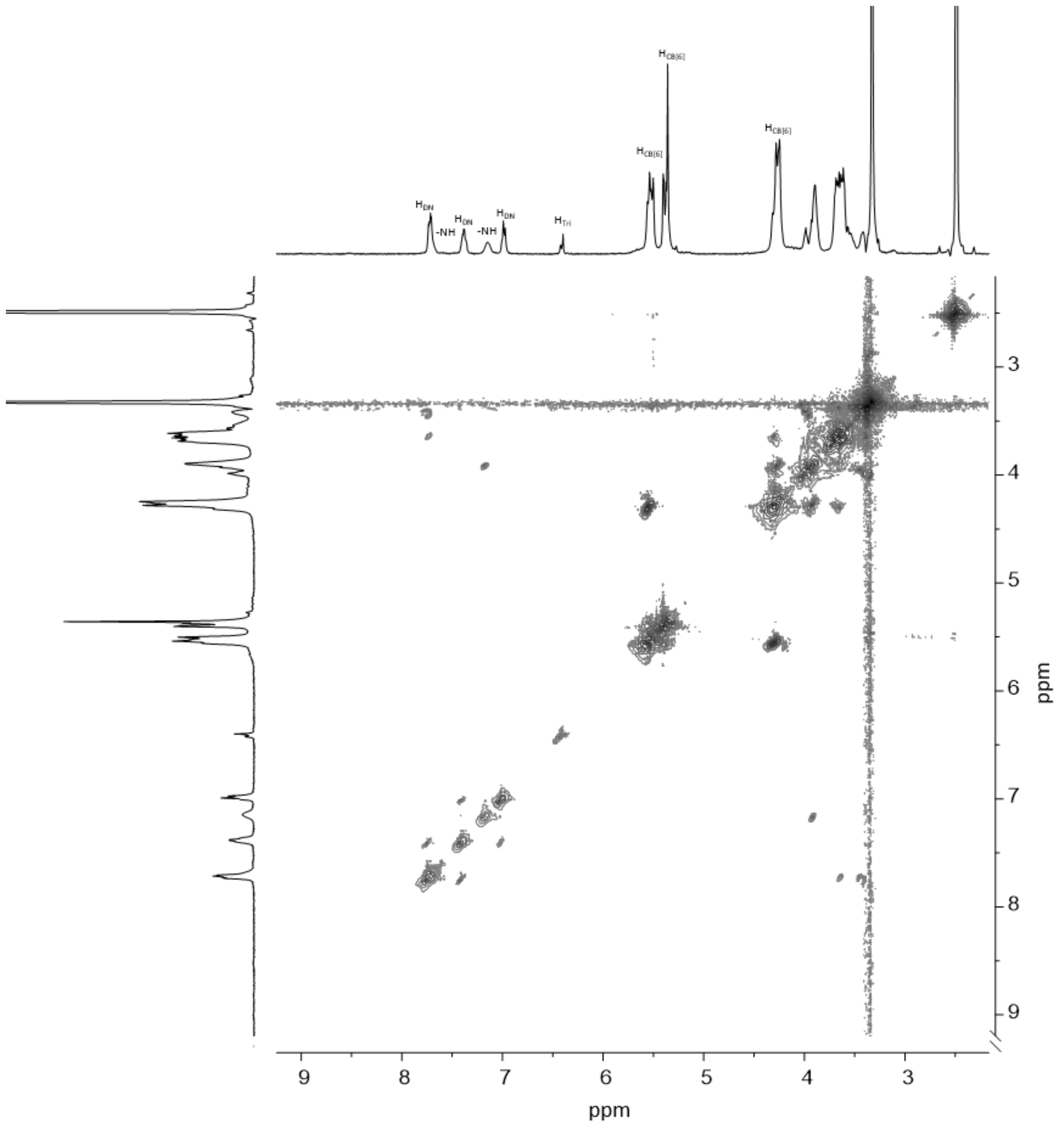

Figure S28. ^{1}H-^{1}H COSY (400 MHz, $(CD_3)_2SO$, 298 K) of **Cat-1**.

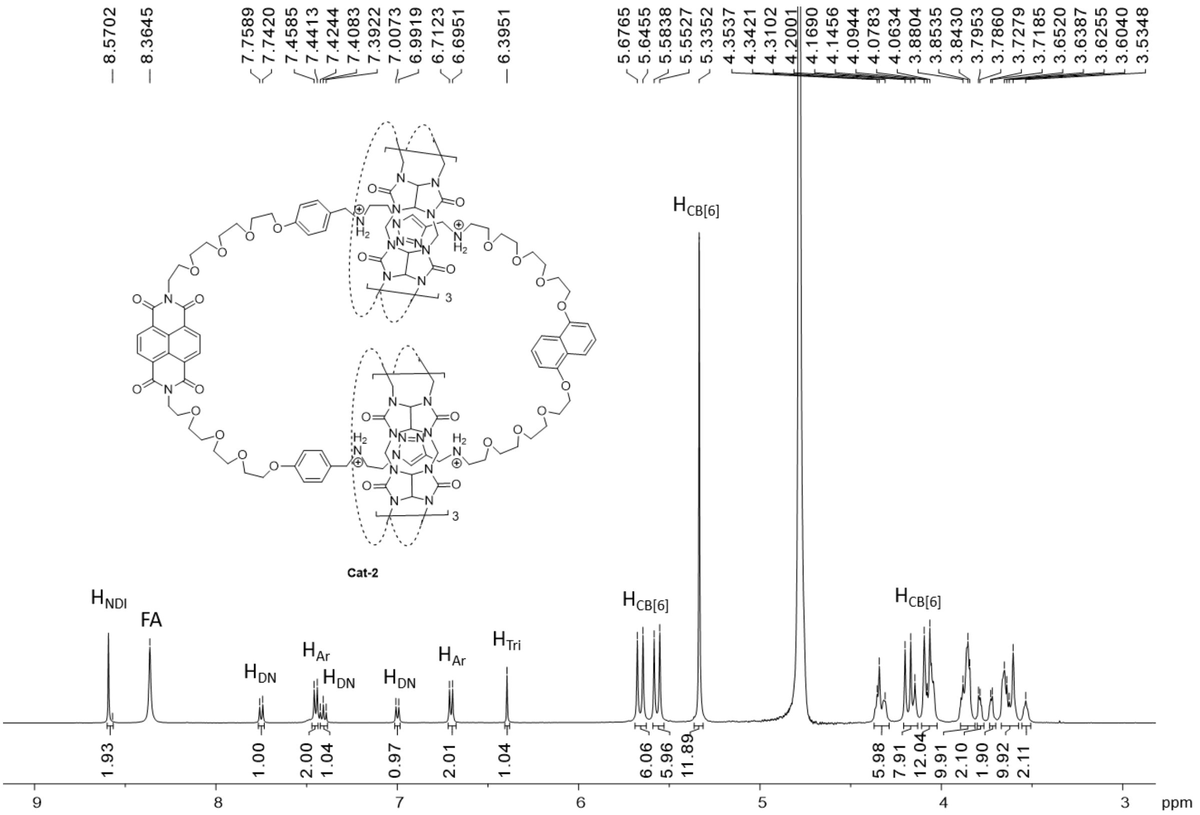

Figure S29. ^{1}H NMR (500 MHz, D_2O, 298 K) of **Cat-2**. Residual formate (FA) is from preparative HPLC.

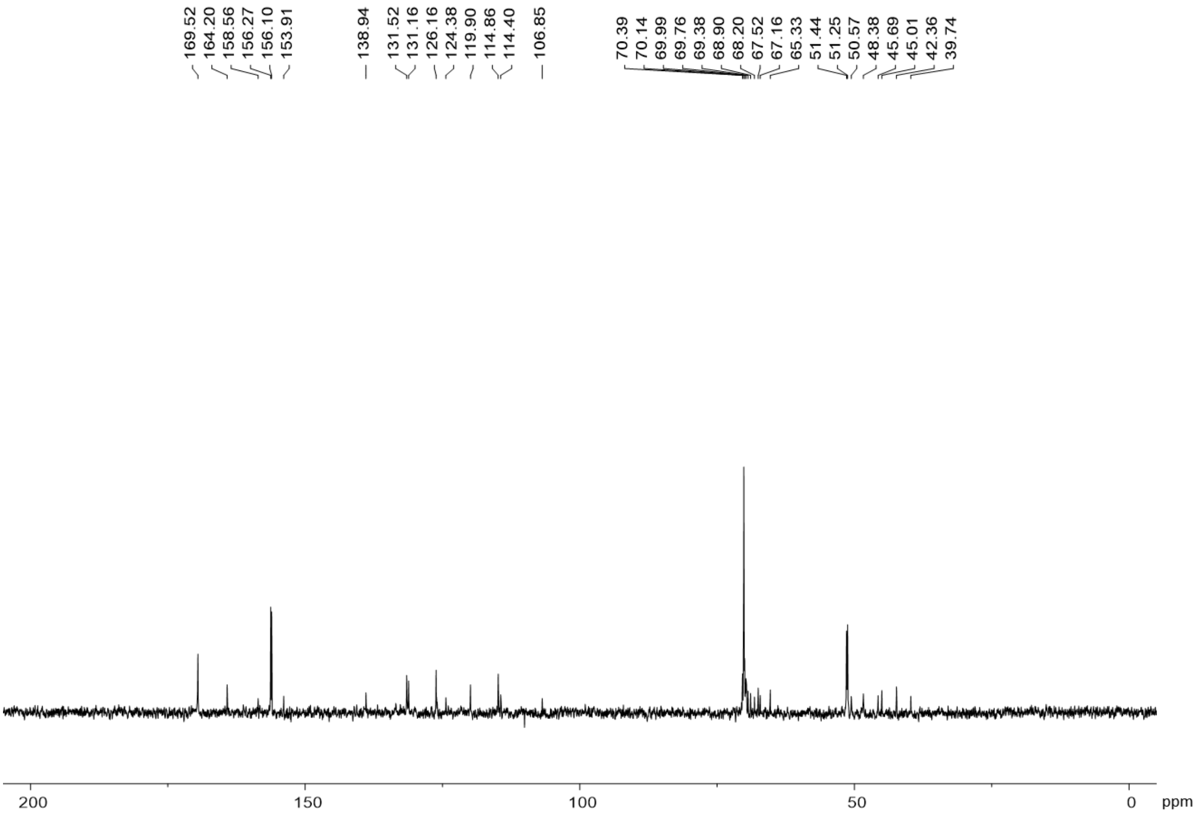

Figure S30. ^{13}C NMR (125 MHz, D_2O, 298 K) of **Cat-2**.

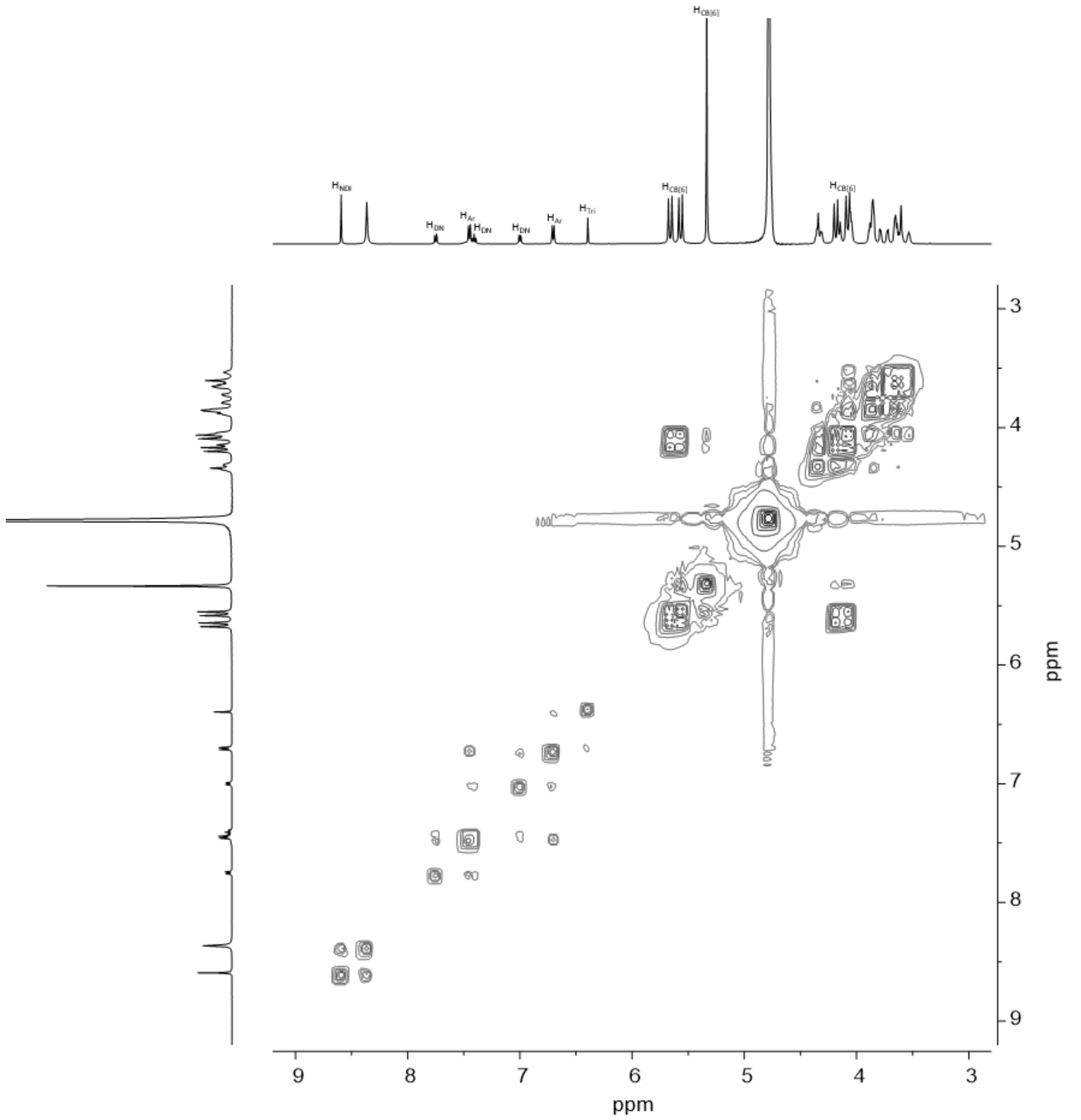

Figure S31. ^{1}H-^{1}H COSY (500 MHz, D_2O, 298 K) of **Cat-2**.

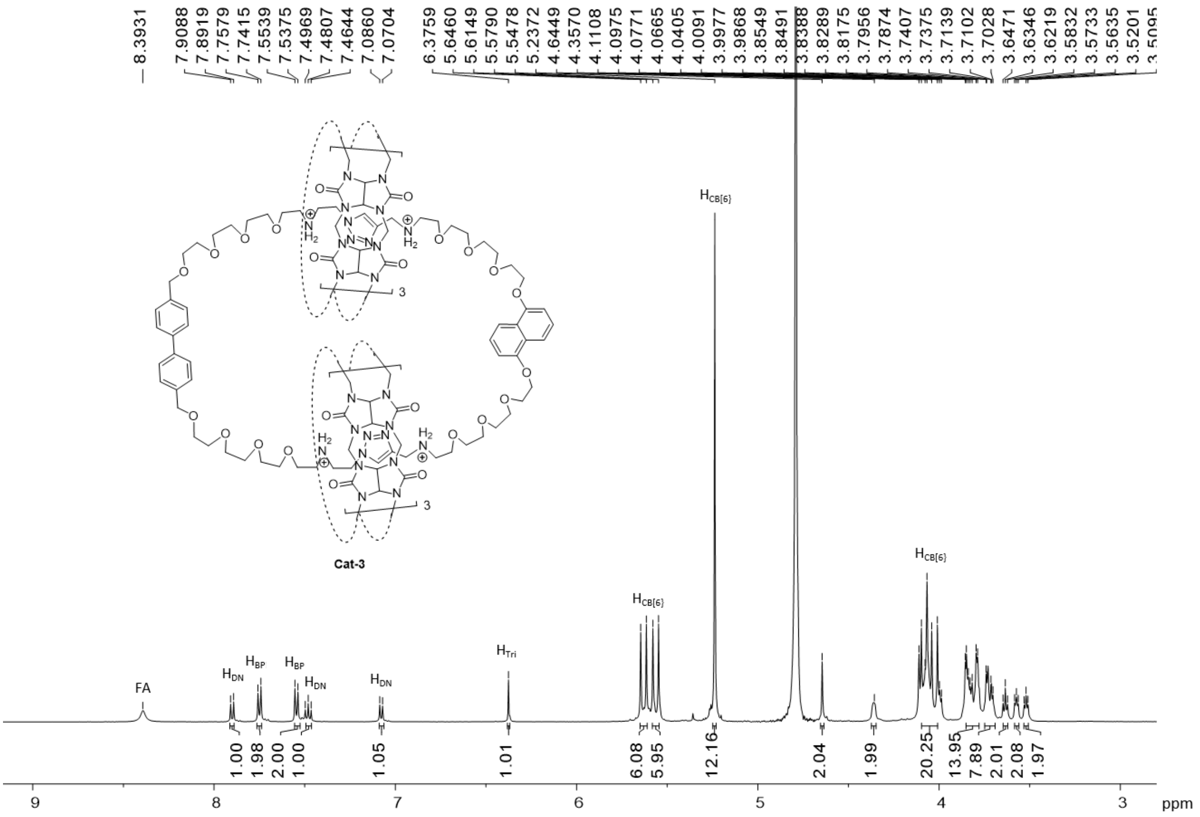

Figure S32. ^{1}H NMR (500 MHz, D_2O, 298 K) of **Cat-3**. Residual formate (FA) is from preparative HPLC.

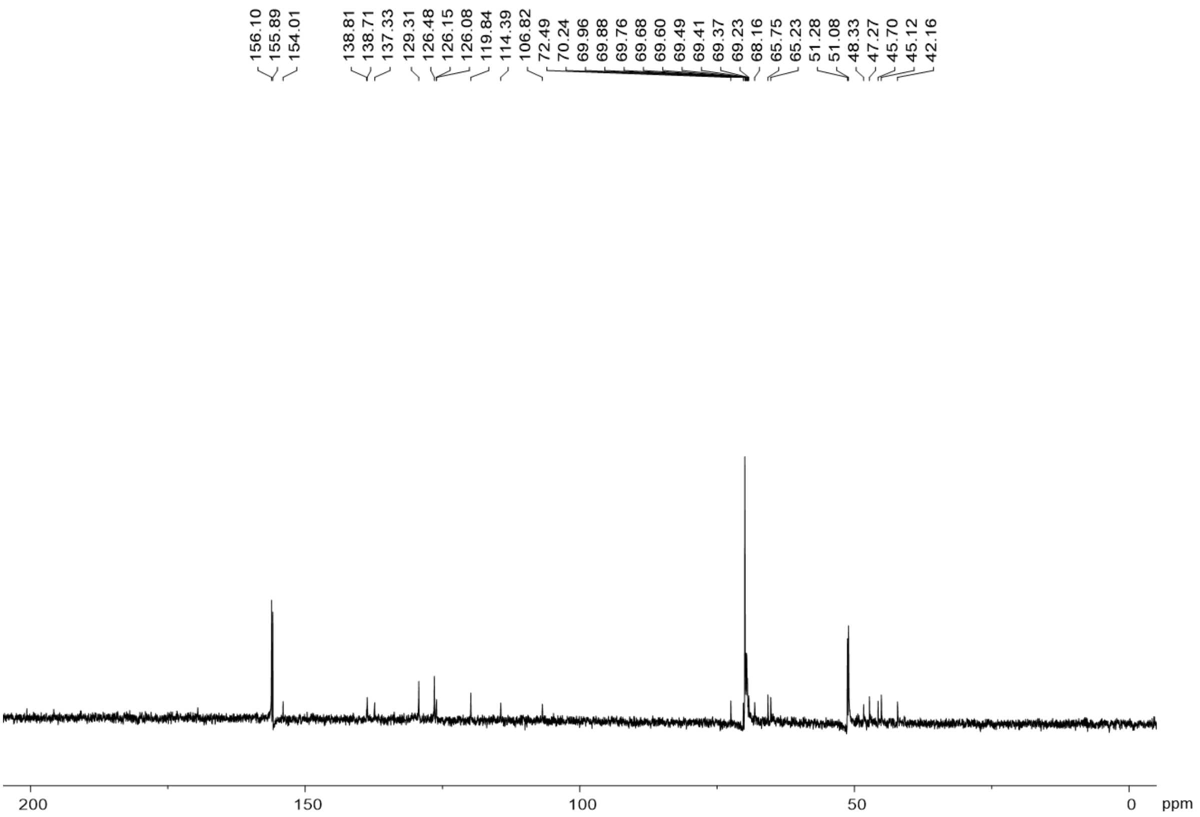

Figure S33. ^{13}C NMR (125 MHz, D_2O, 298 K) of **Cat-3**.

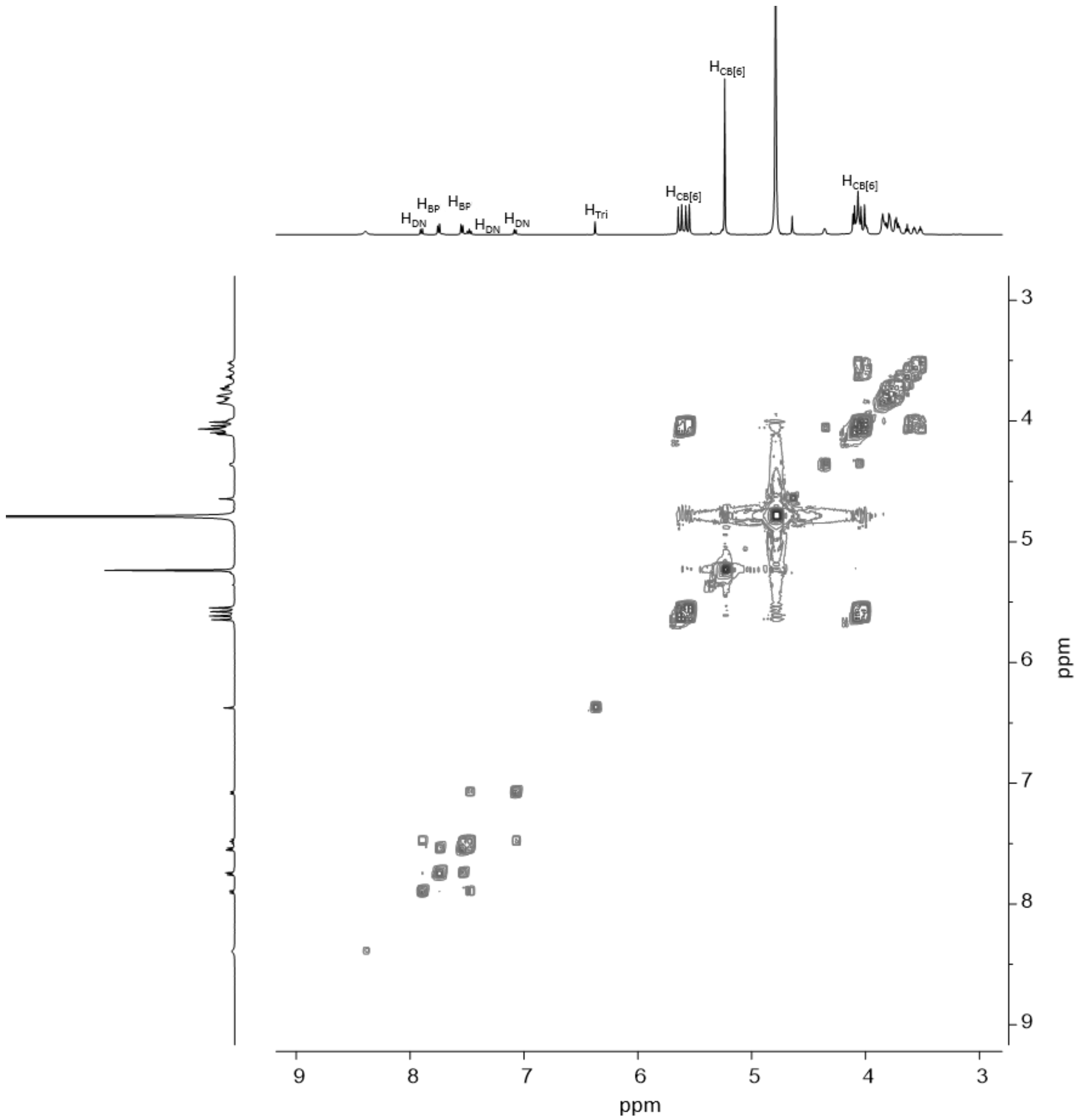

Figure S34. ^1H-^1H COSY (500 MHz, D_2O, 298 K) of **Cat-3**.

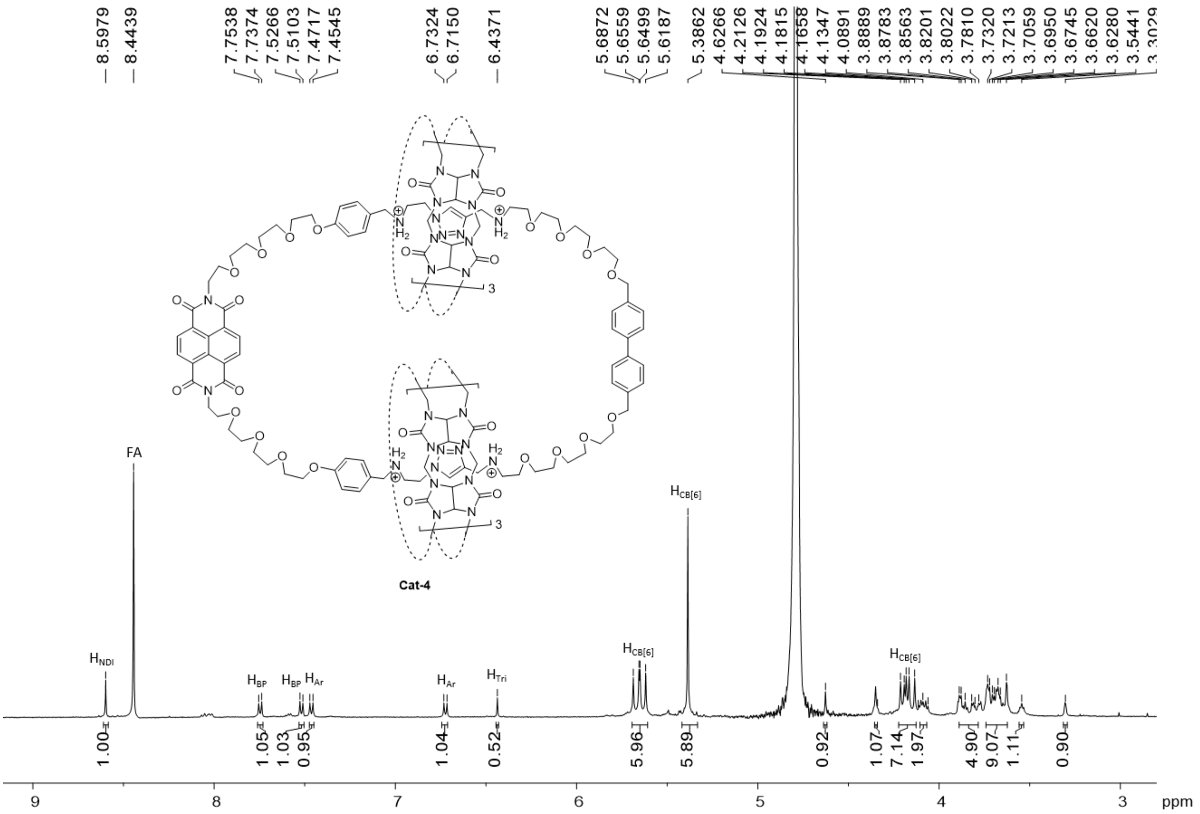

Figure S35. ^{1}H NMR (500 MHz, D_2O, 298 K) of **Cat-4**. Residual formate (FA) is from preparative HPLC.

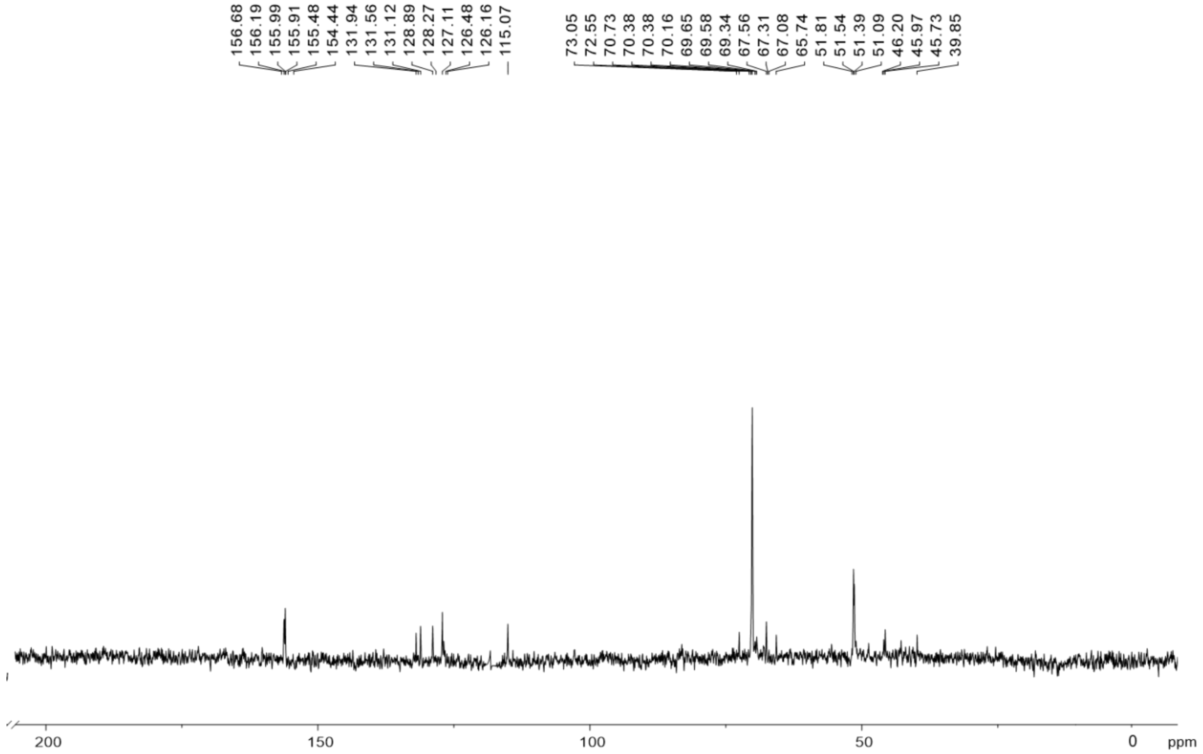

Figure S36. ^{13}C NMR (125 MHz, D_2O, 298 K) of **Cat-4**.

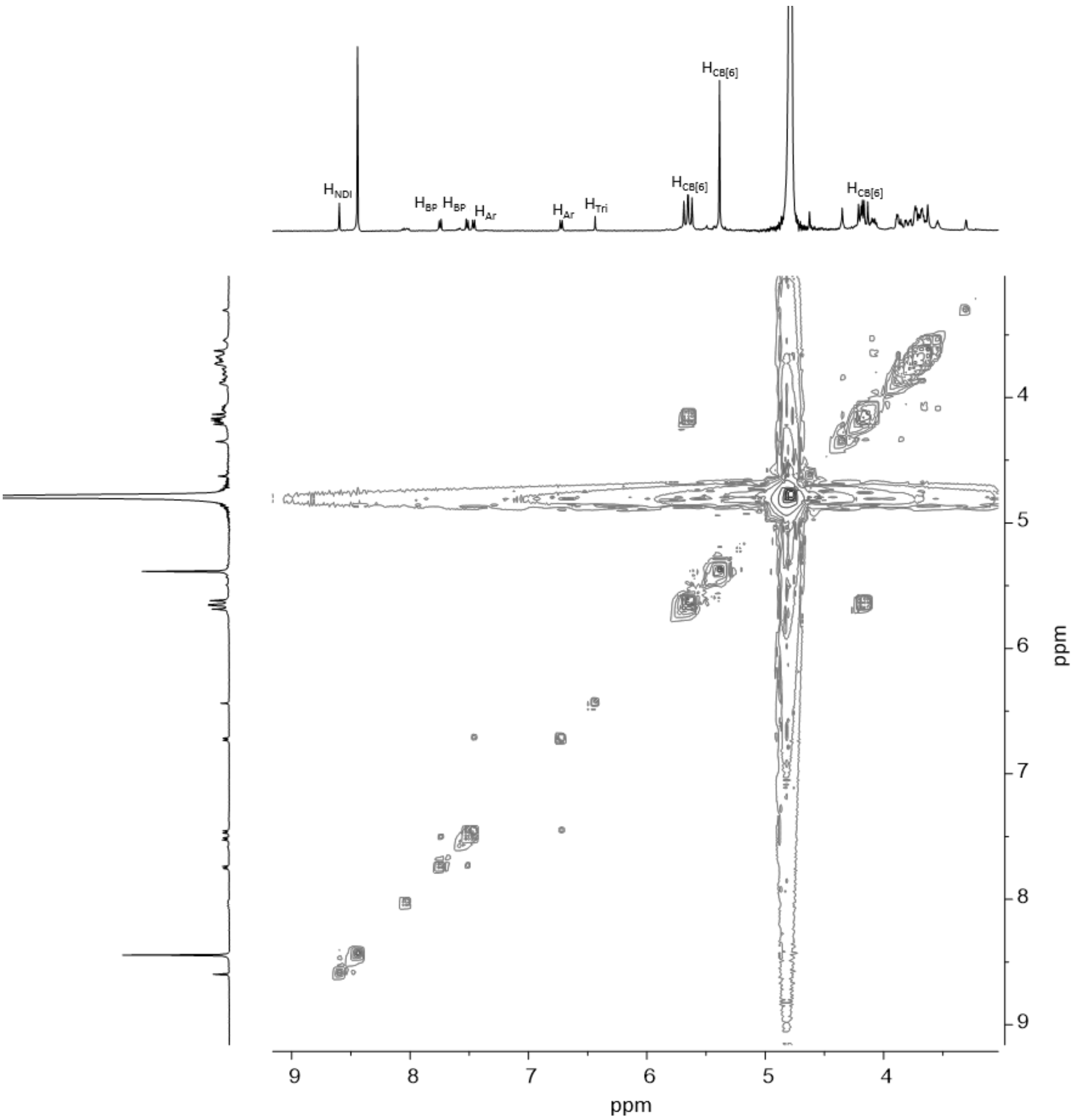

Figure S37. ^{1}H-^{1}H COSY (500 MHz, D_2O, 298 K) of **Cat-4**.

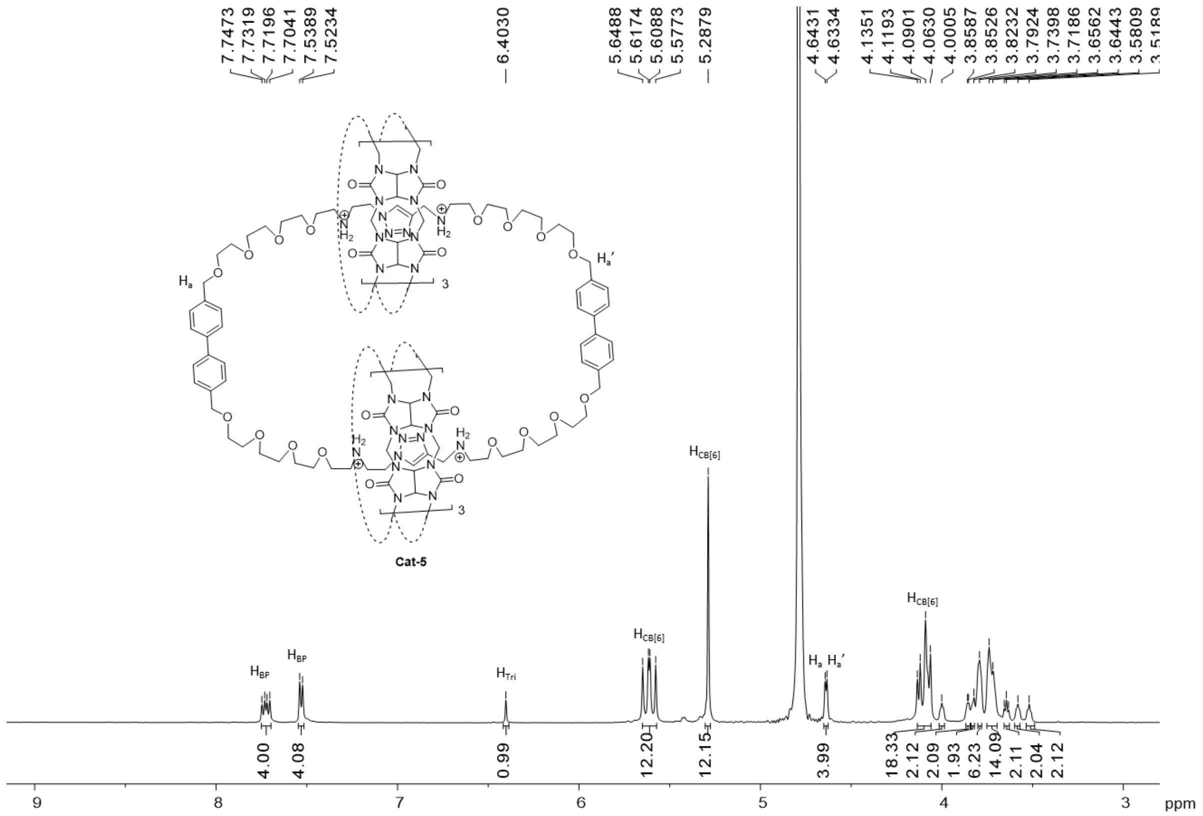

Figure S38. ^{1}H NMR (500 MHz, D_2O, 298 K) of **Cat-5.**

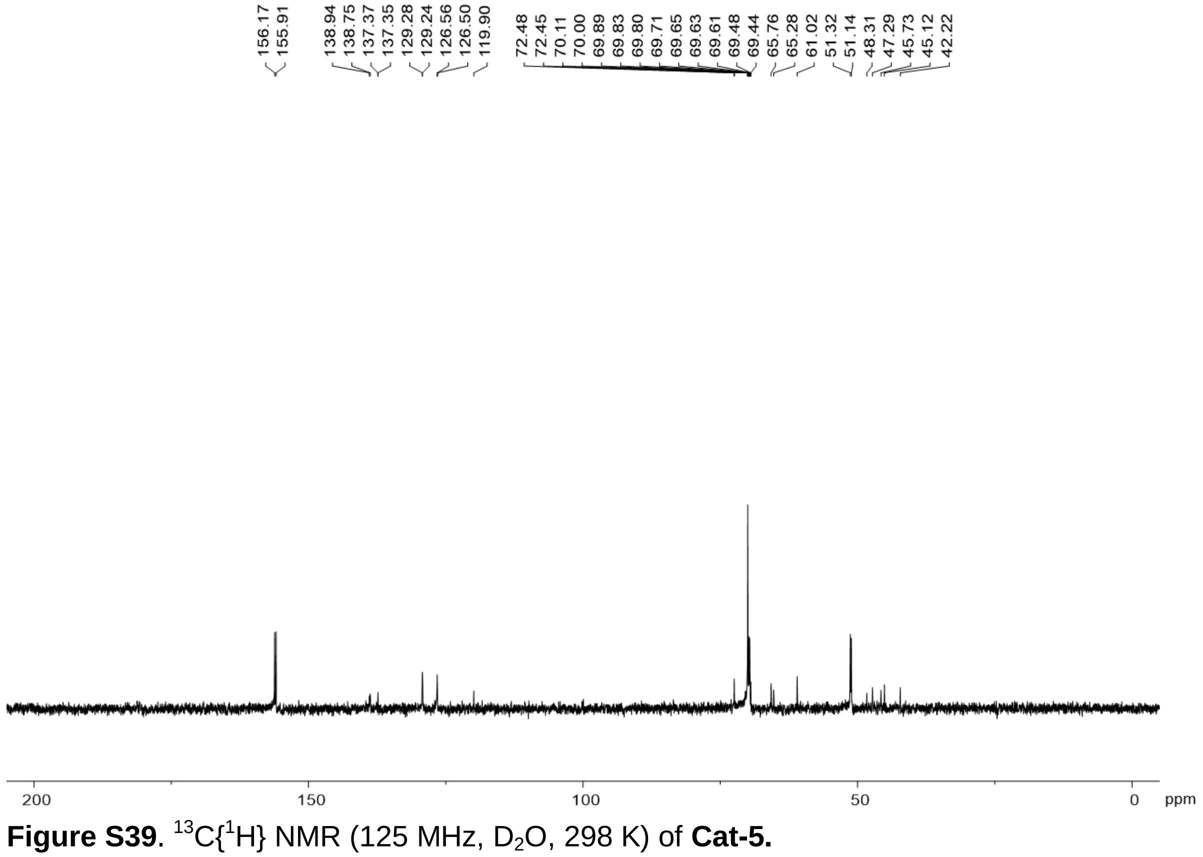

Figure S39. $^{13}C\{^{1}H\}$ NMR (125 MHz, D_2O, 298 K) of **Cat-5.**

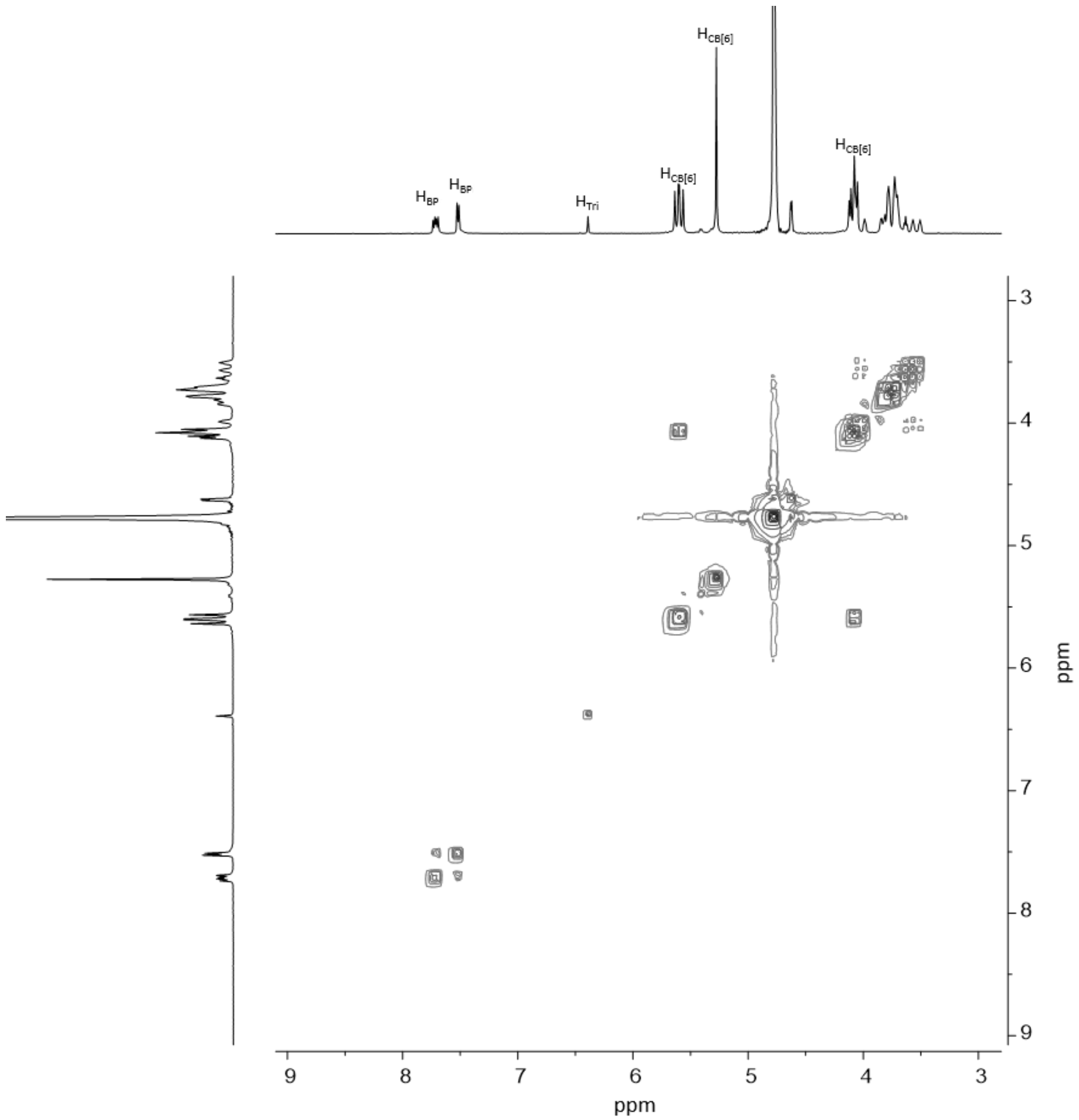

Figure S40. ^{1}H-^{1}H COSY (500 MHz, D_2O, 298 K) of **Cat-5**.

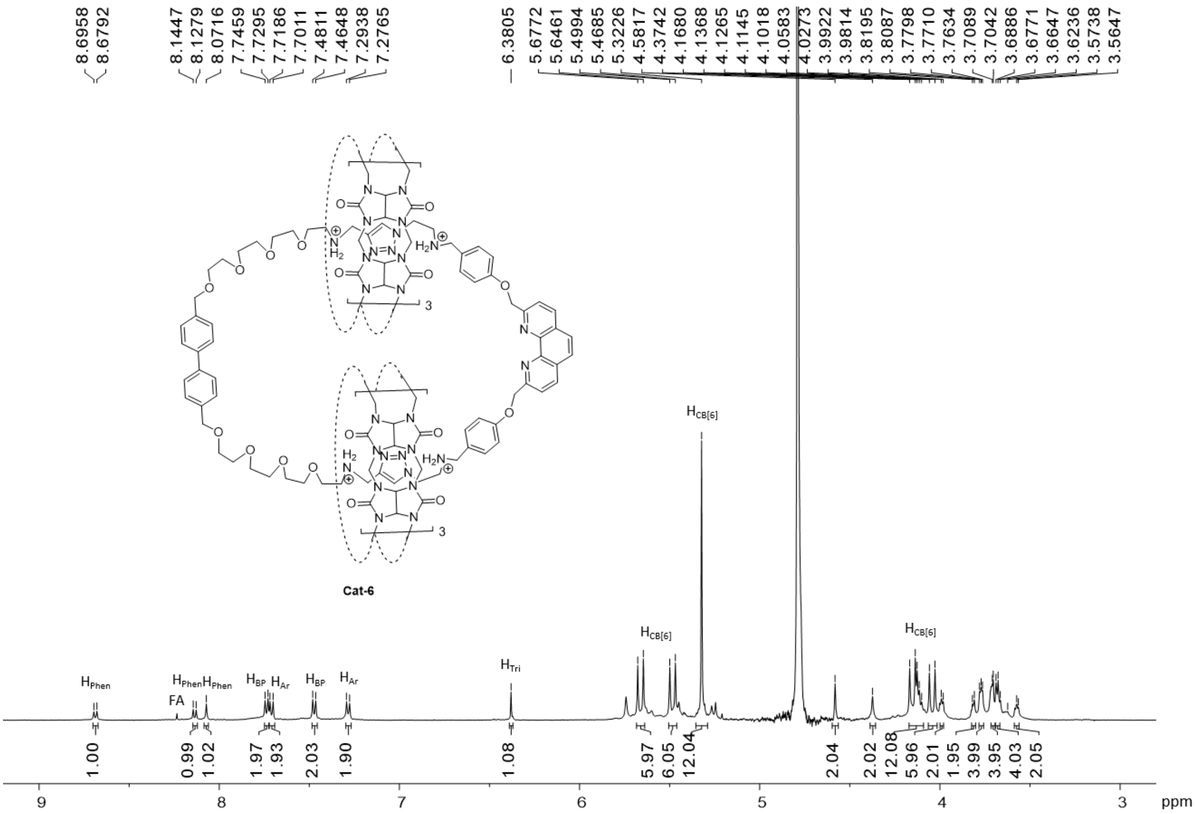

Figure S41. ^{1}H NMR (500 MHz, D_2O, 298 K) of **Cat-6.** Residual formate (FA) is from preparative HPLC.

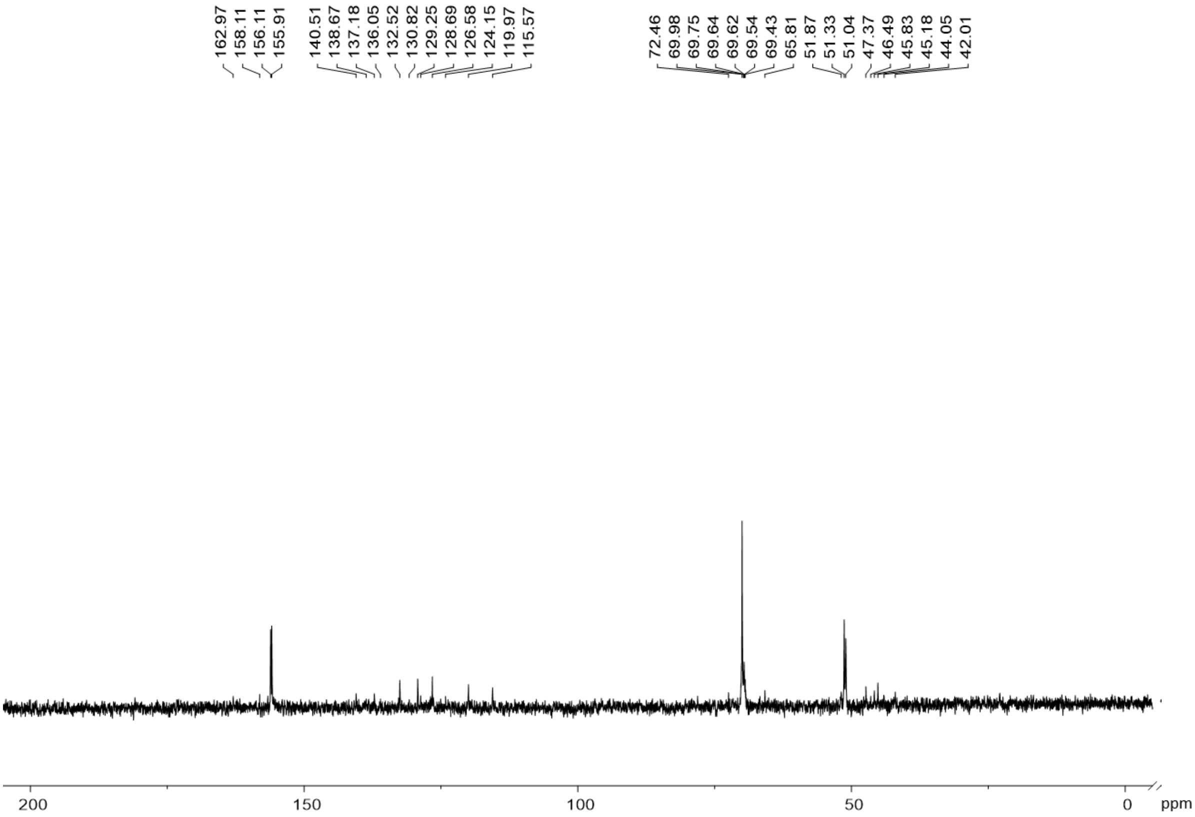

Figure S42. $^{13}C\{^1H\}$ NMR (125 MHz, D_2O, 298 K) of **Cat-6.**

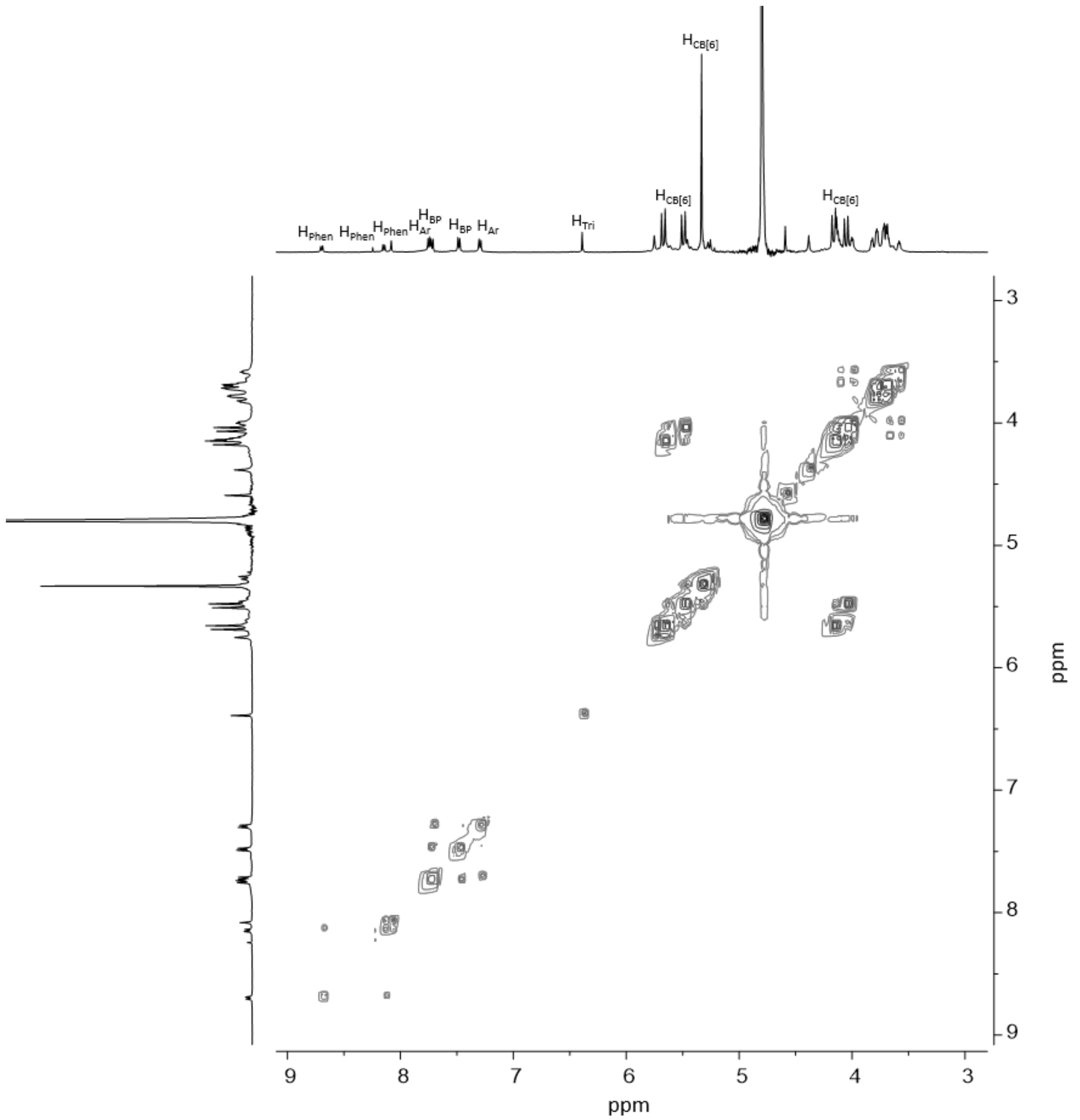

Figure S43. ^{1}H-^{1}H COSY (500 MHz, D_2O, 298 K) of **Cat-6**.

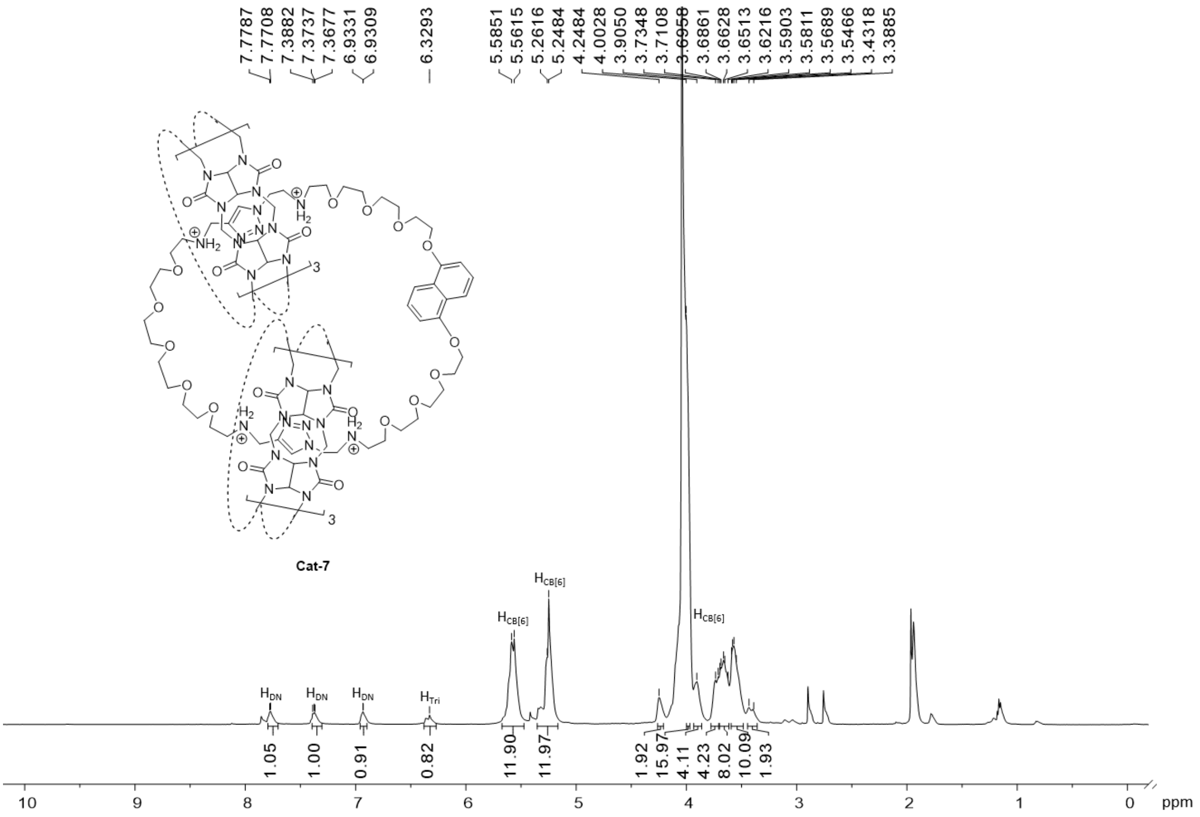

Figure S44. ^{1}H NMR (500 MHz, CD_3CN/D_2O v/v 10:1, 298 K) of **Cat-7.**

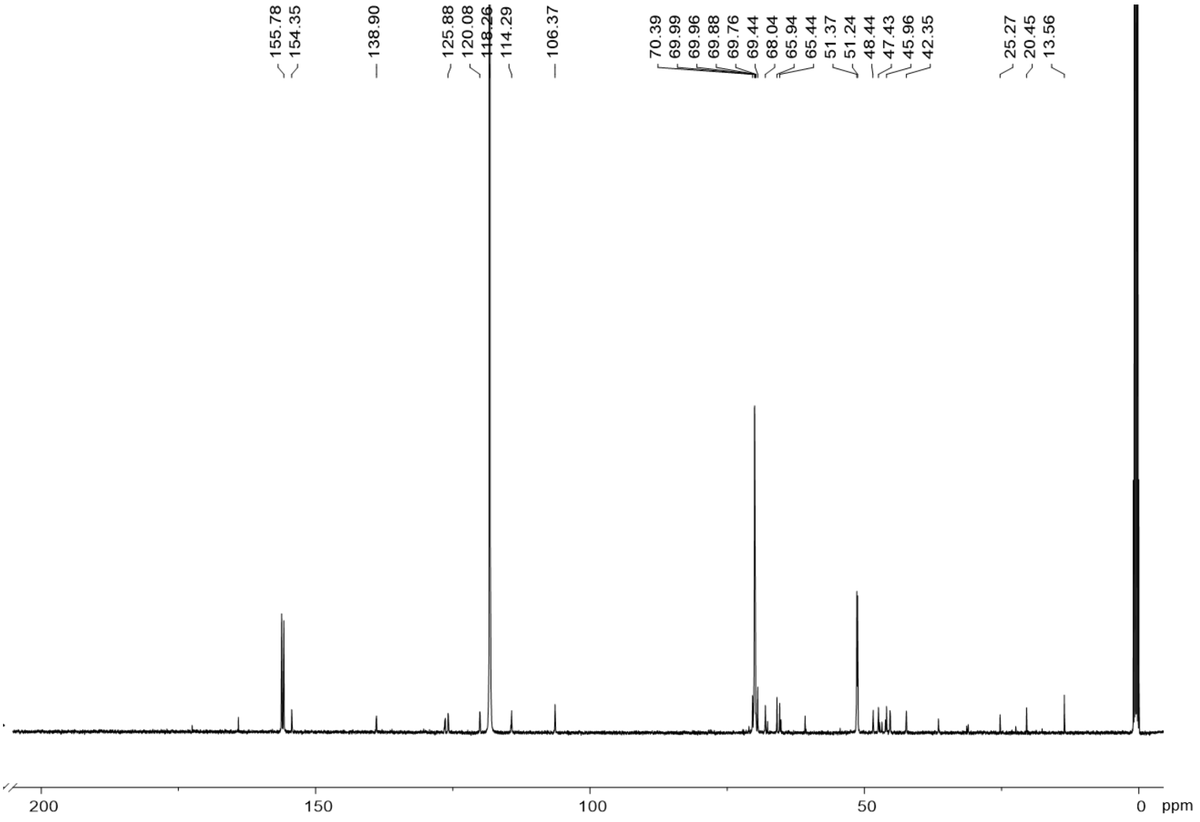

Figure S45. $^{13}C\{^1H\}$ NMR (125 MHz, CD_3CN/D_2O v/v 10:1, 298 K) of **Cat-7.**

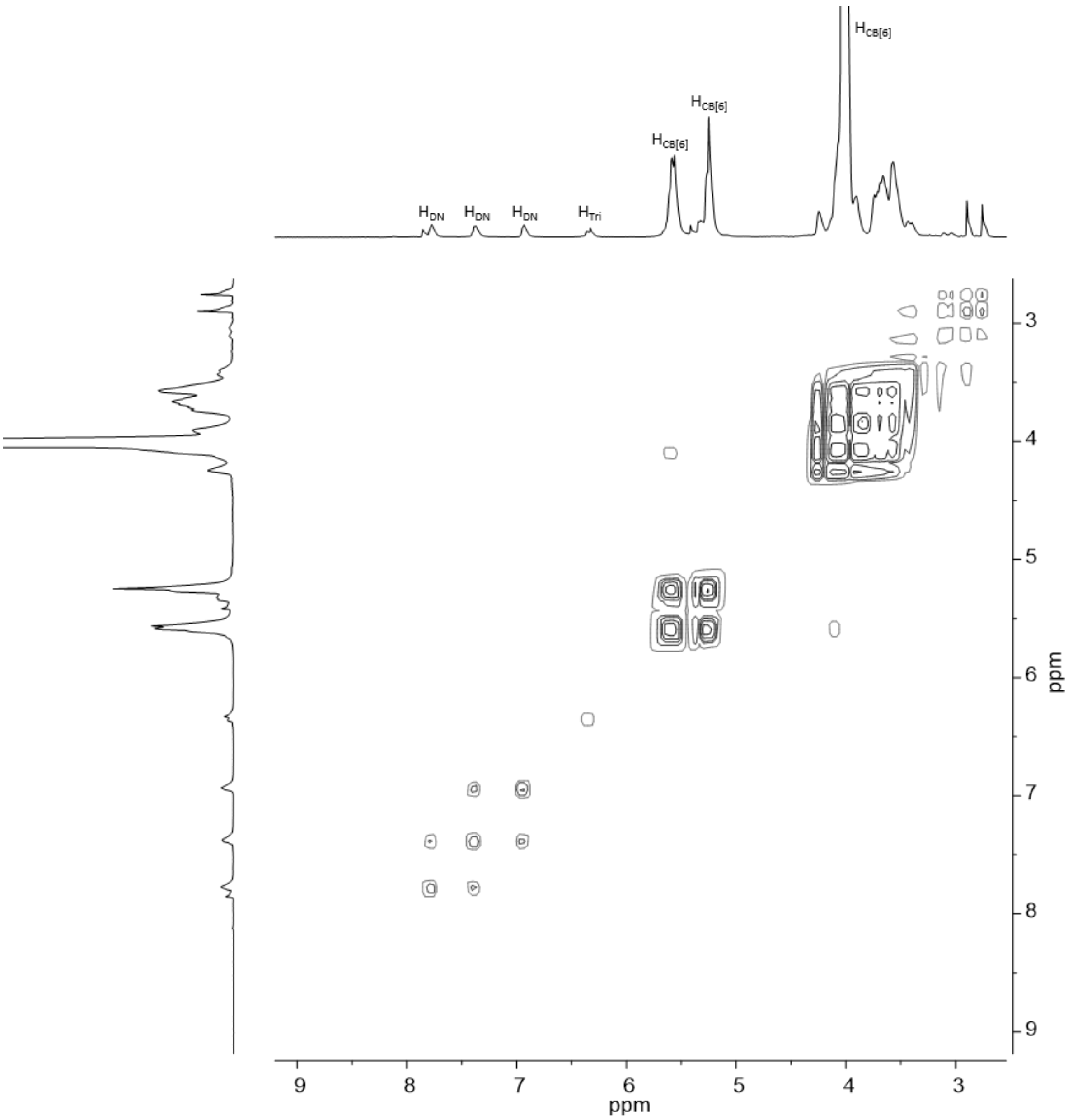

Figure S46. ^1H-^1H COSY (500 MHz, CD_3CN/D_2O v/v 10:1, 298 K) of **Cat-7**.

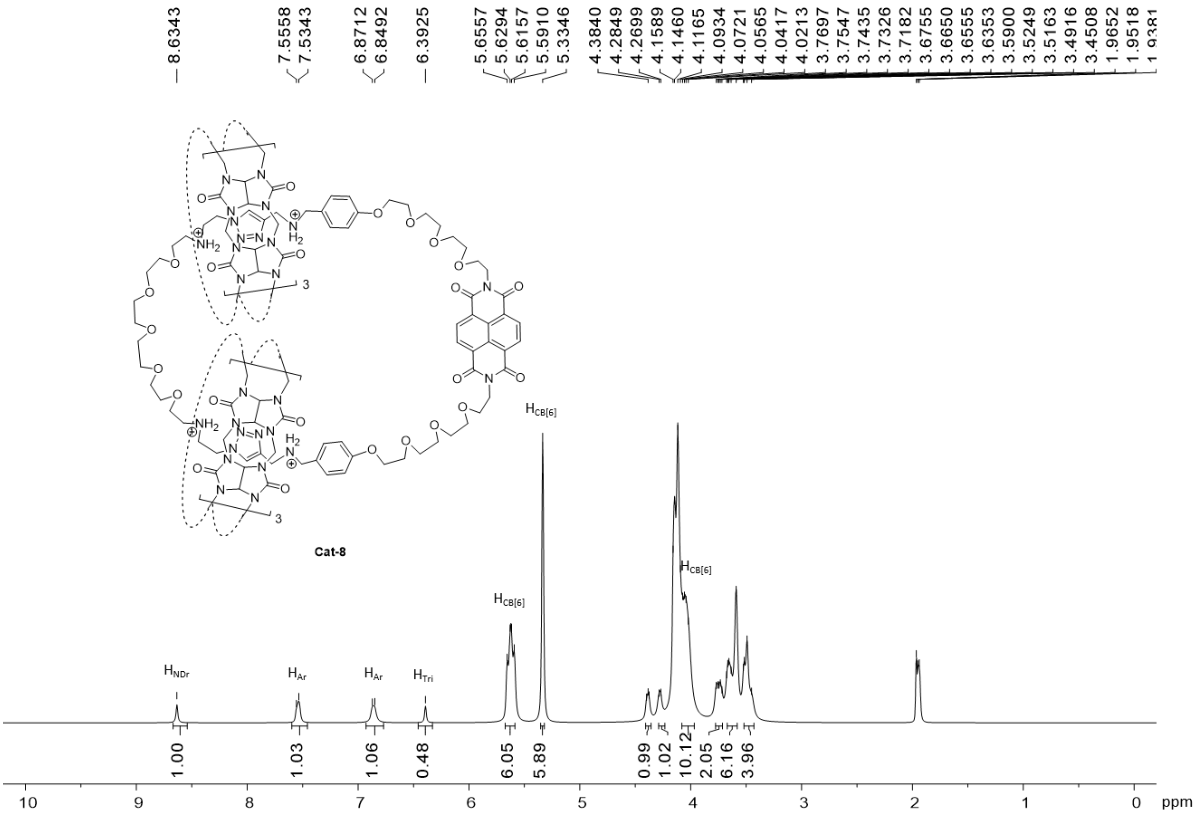

Figure S47. ^{1}H NMR (500 MHz, CD_3CN/D_2O v/v 10:1, 298 K) of **Cat-8.**

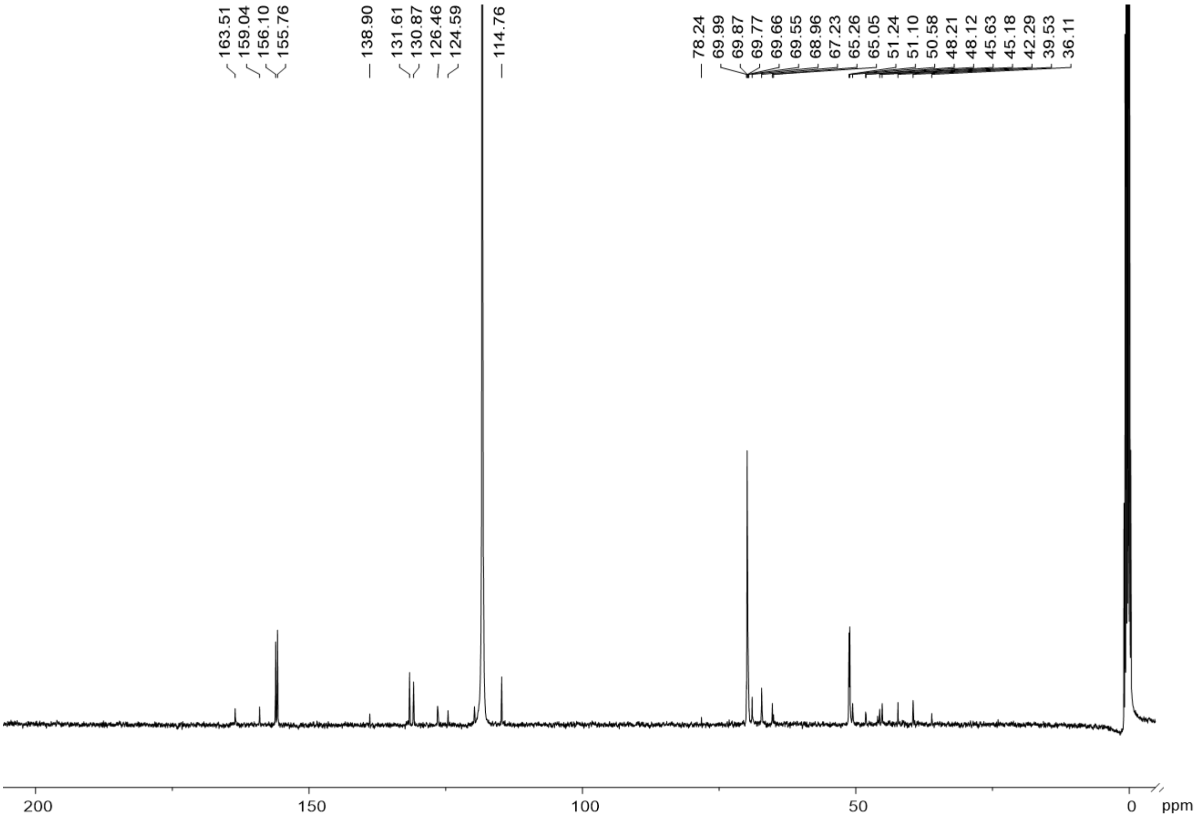

Figure S48. $^{13}C\{^1H\}$ NMR (125 MHz, CD_3CN/D_2O v/v 10:1, 298 K) of **Cat-8.**

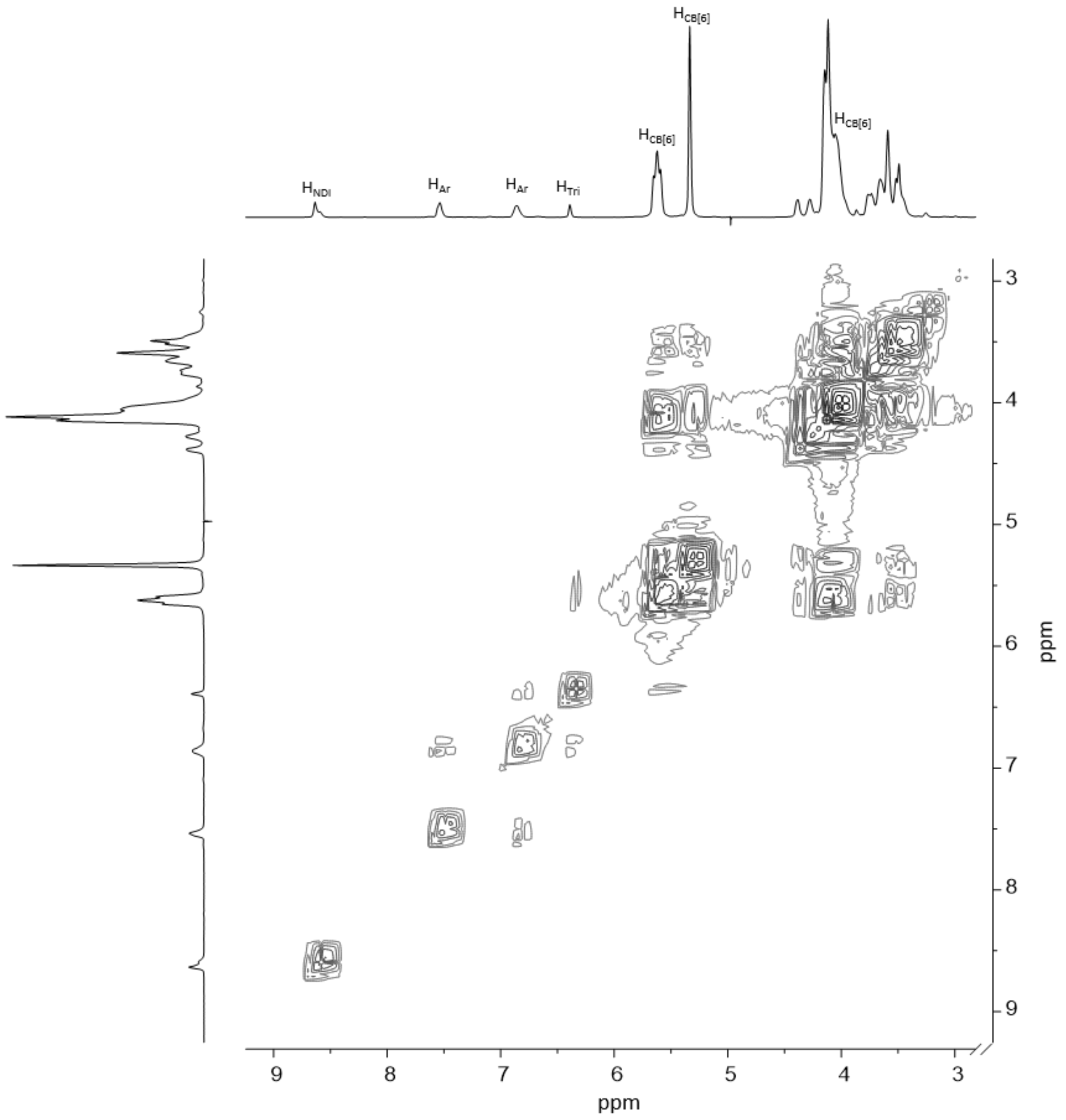

Figure S49. ^{1}H-^{1}H COSY (500 MHz, CD_3CN/D_2O v/v 10:1, 298 K) of **Cat-8**.

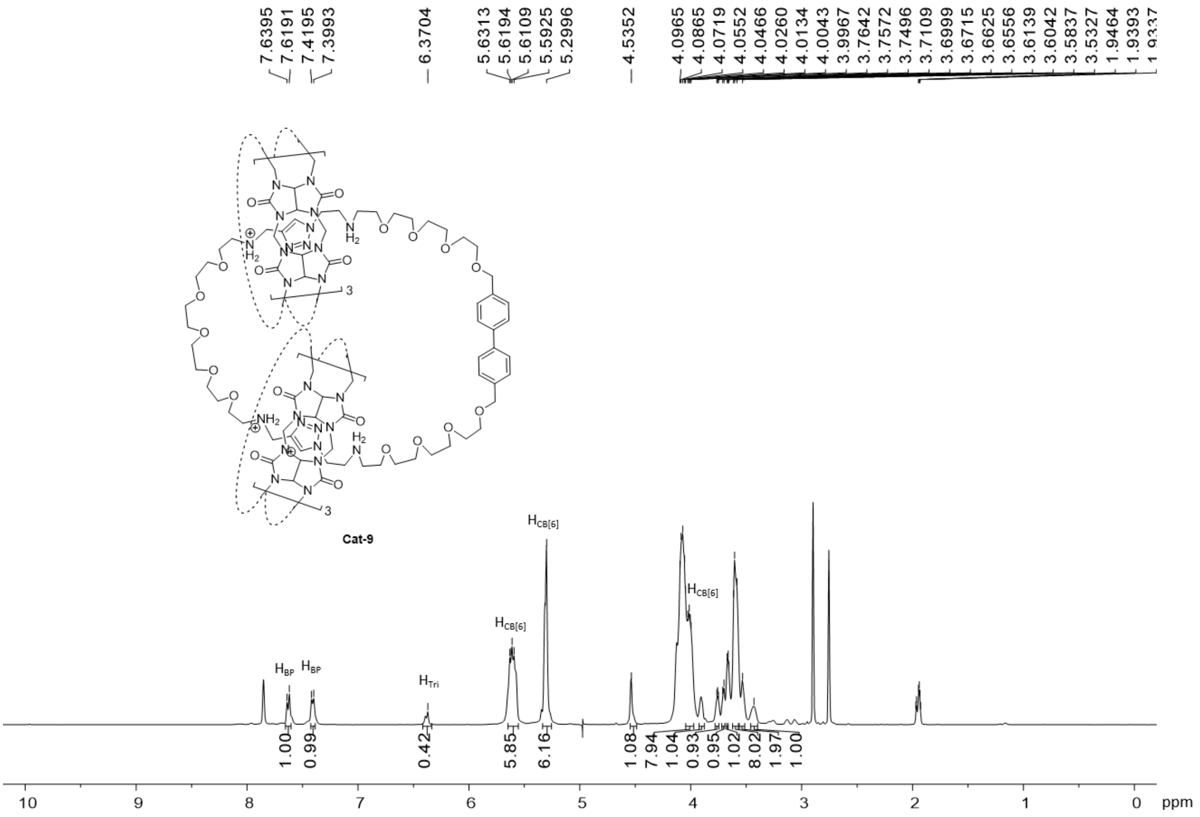

Figure S50. ^{1}H NMR (500 MHz, CD_3CN/D_2O v/v 10:1, 298 K) of **Cat-9.**

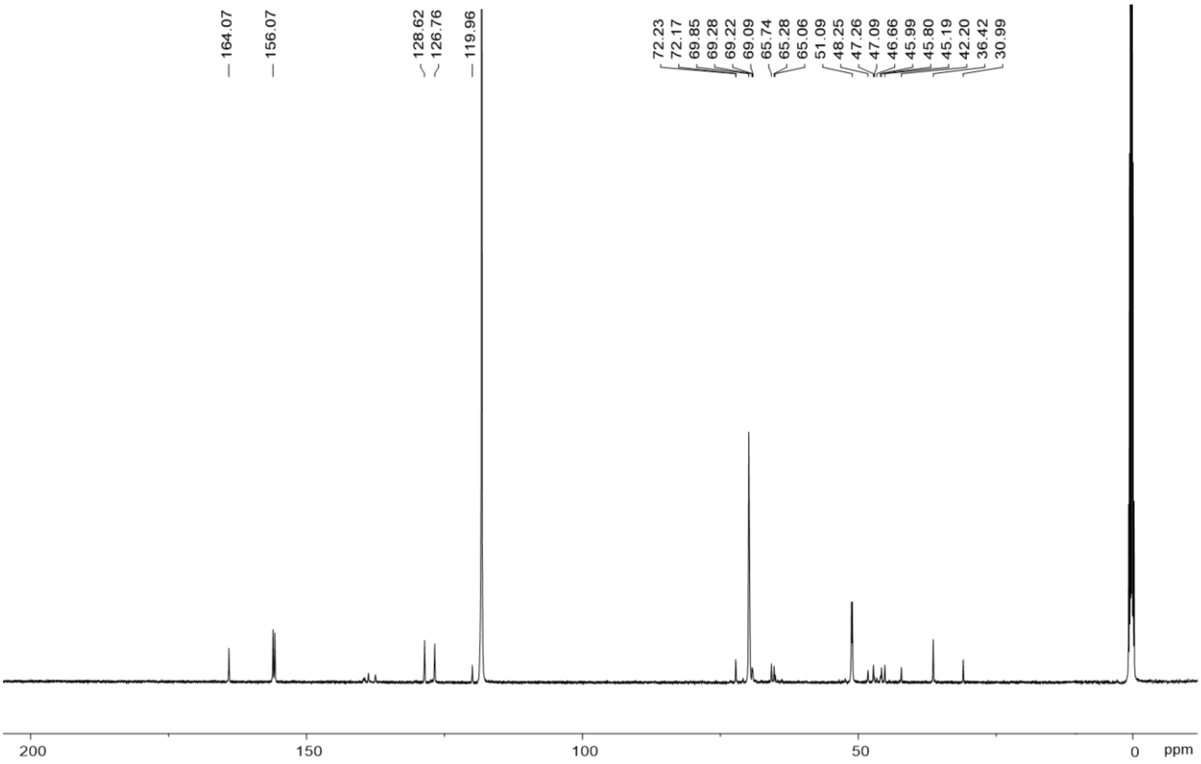

Figure S51. $^{13}C\{^{1}H\}$ NMR (125 MHz, CD_3CN/D_2O v/v 10:1, 298 K) of **Cat-9.**

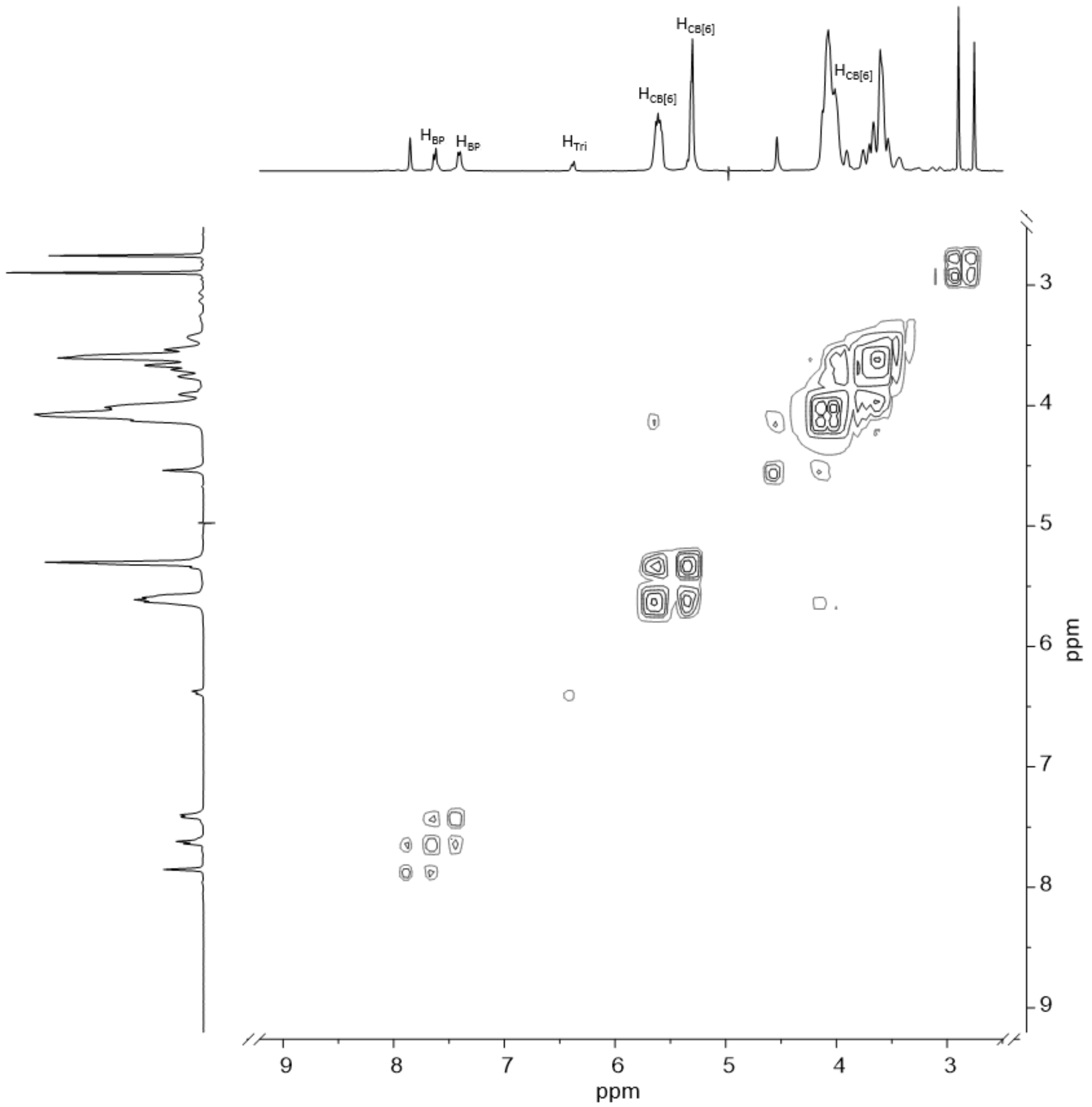

Figure S52. ^1H-^1H COSY (500 MHz, CD_3CN/D_2O v/v 10:1, 298 K) of **Cat-9**.

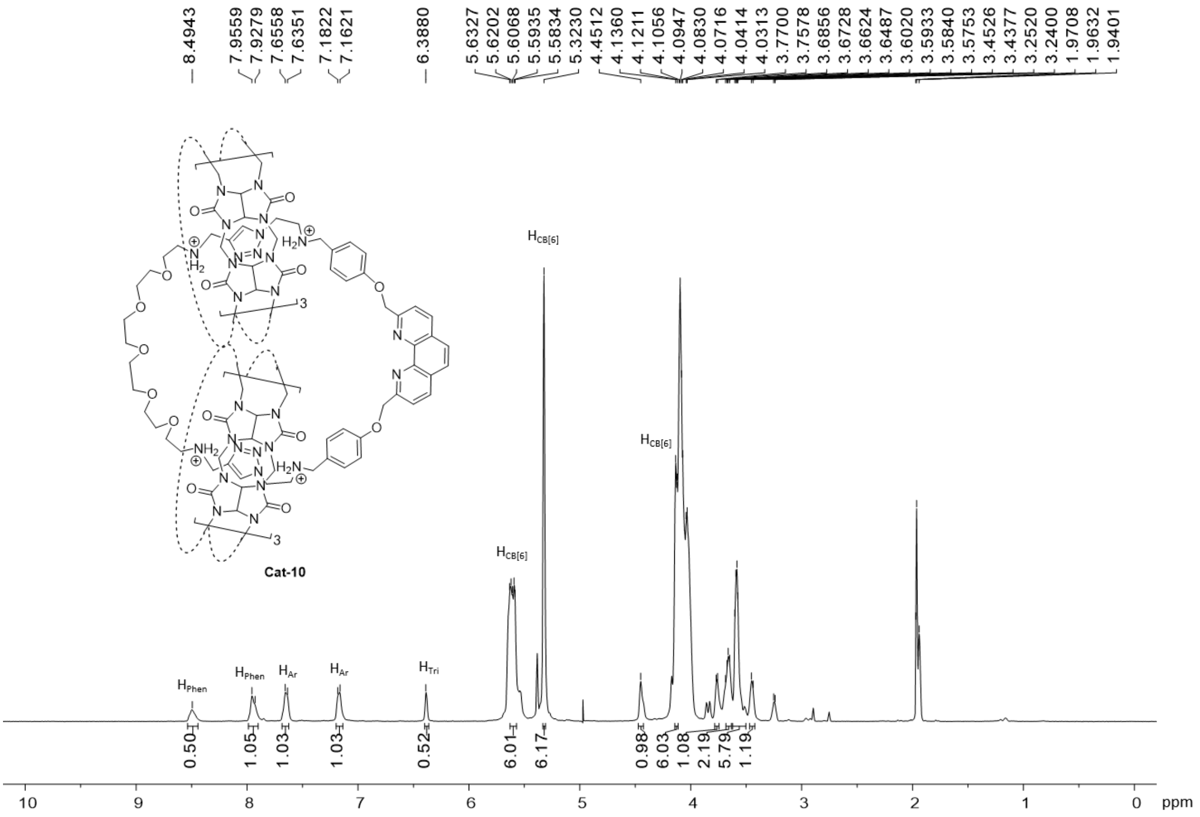

Figure S53. ^{1}H NMR (500 MHz, CD_3CN/D_2O v/v 10:1, 298 K) of **Cat-10.**

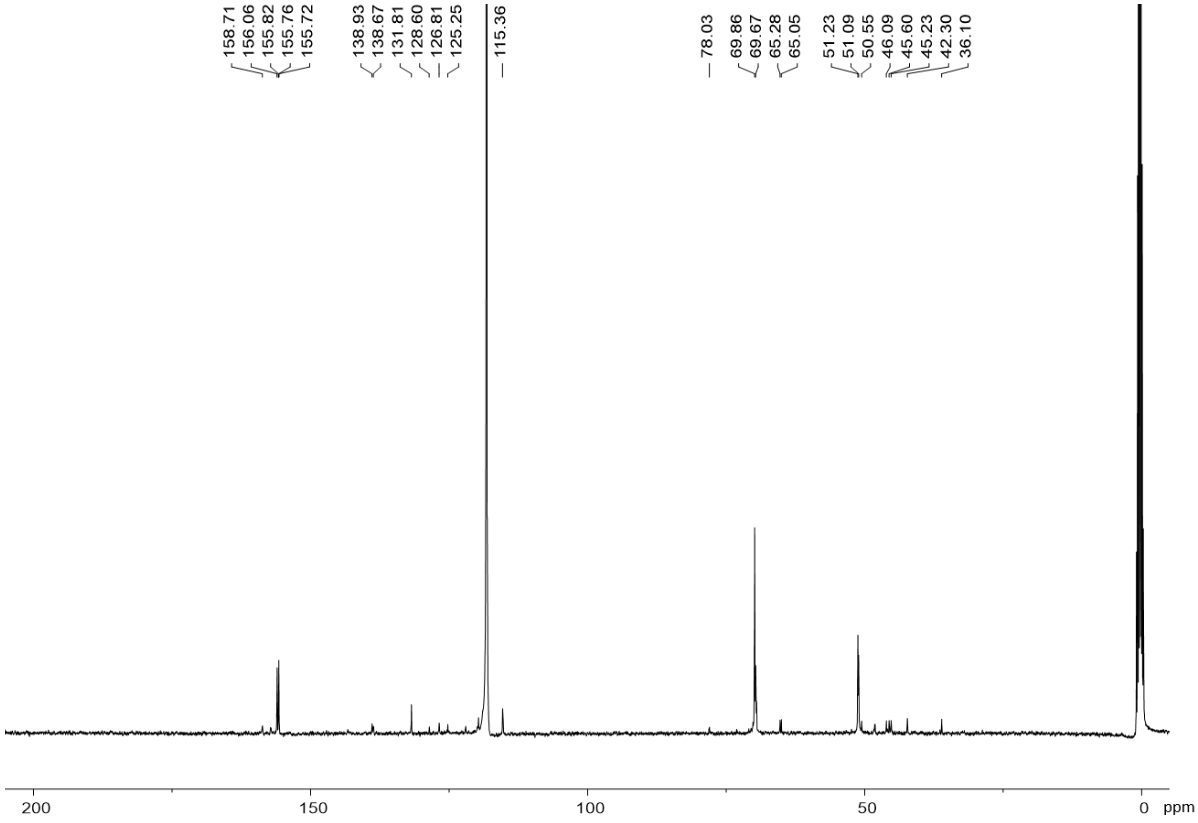

Figure S54. $^{13}C\{^{1}H\}$ NMR (100 MHz, CD_3CN/D_2O v/v 10:1, 298 K) of **Cat-10.**

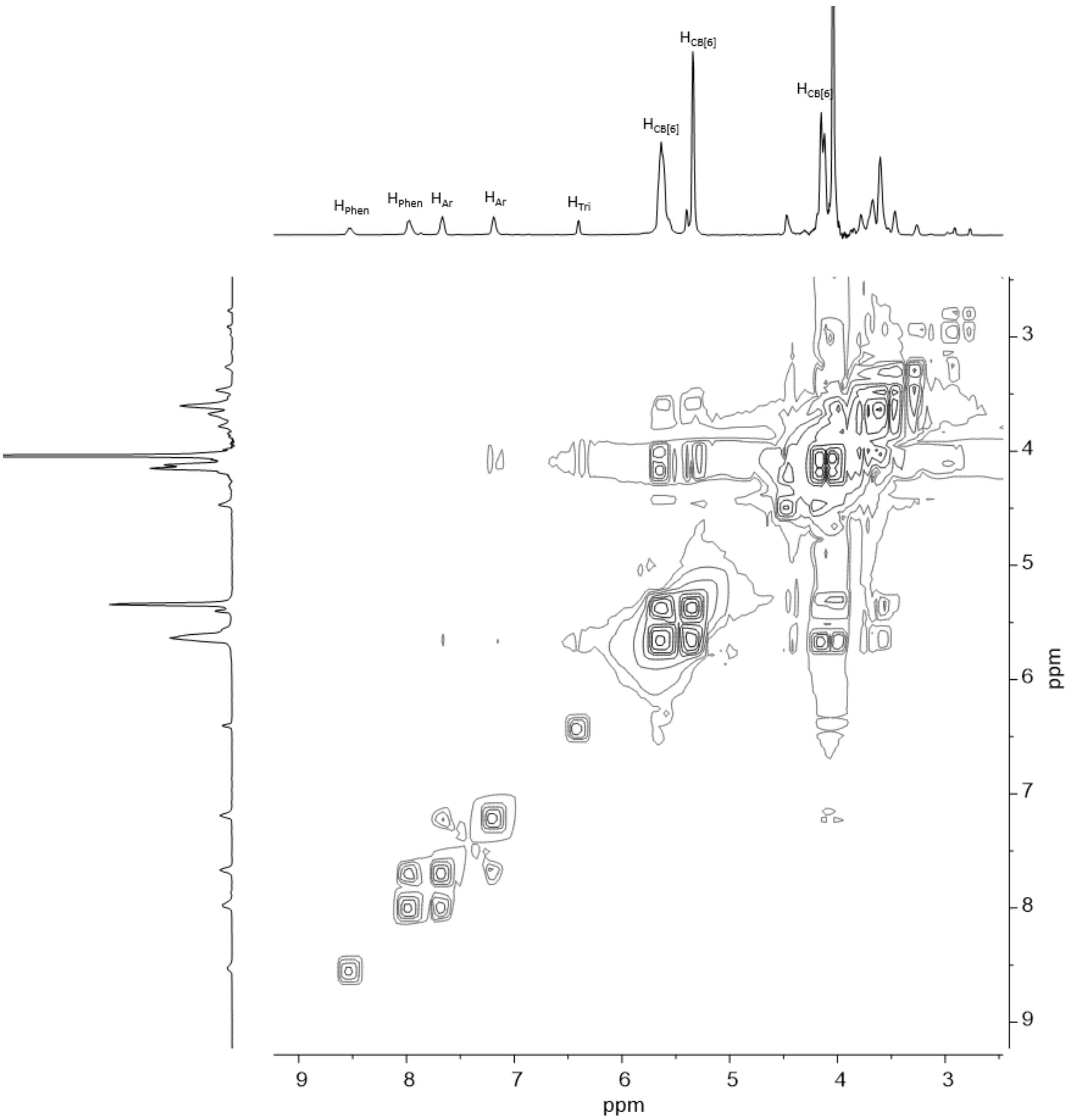

Figure S55. ^{1}H-^{1}H COSY (500 MHz, CD_3CN/D_2O v/v 10:1, 298 K) of **Cat-10**.

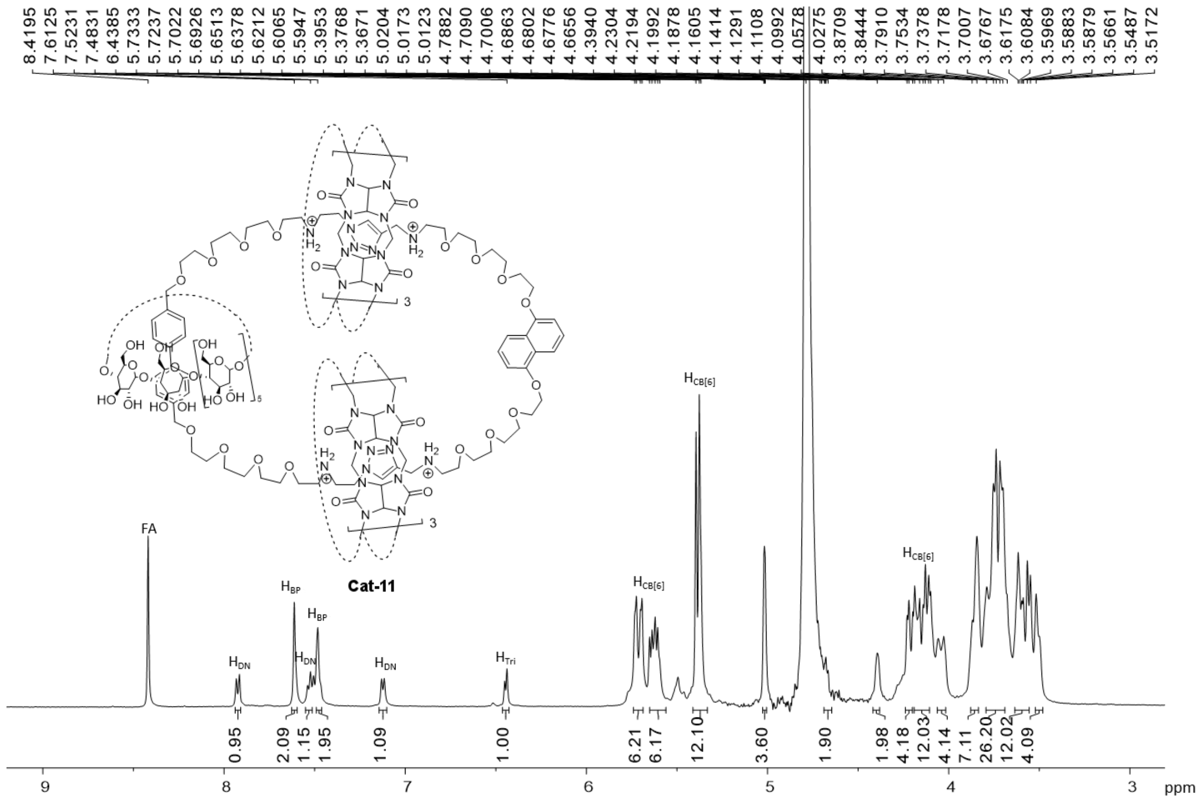

Figure S56. ^{1}H NMR (500 MHz, D_2O, 298 K) of **Cat-11.** Residual formate (FA) is from preparative HPLC.

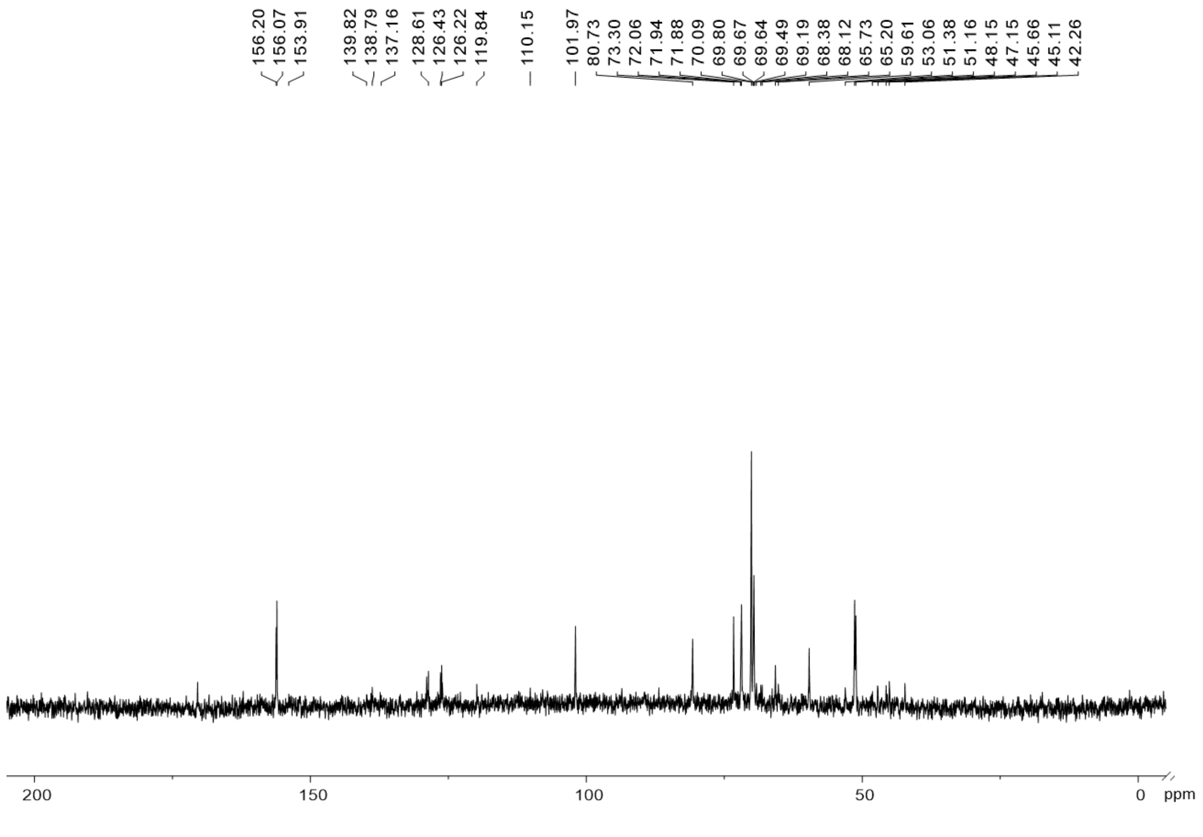

Figure S57. $^{13}C\{^{1}H\}$ NMR (125 MHz, D_2O, 298 K) of **Cat-11.**

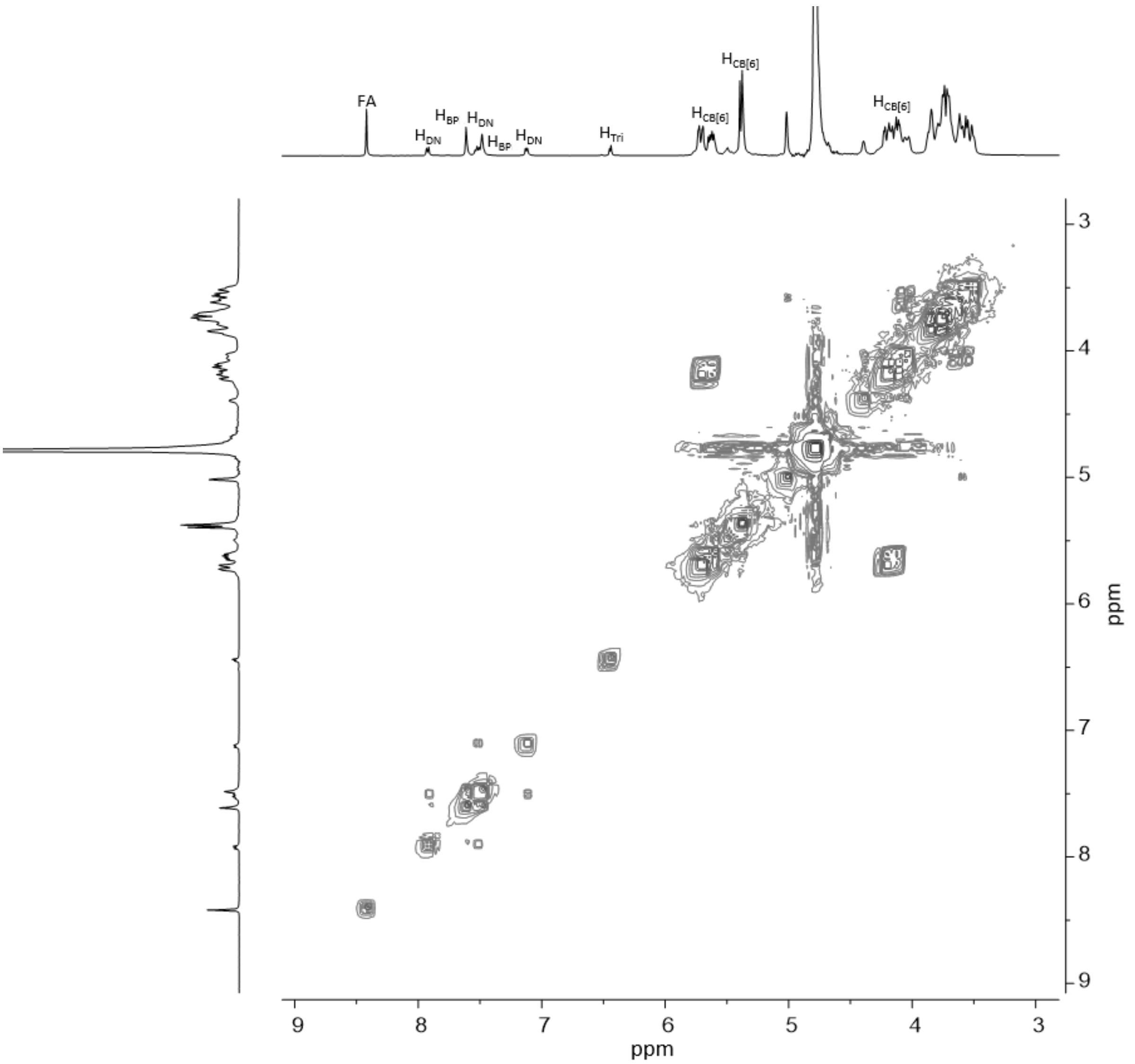

Figure S58. ^{1}H-^{1}H COSY (500 MHz, D_2O, 298 K) of **Cat-11**.

4. ESIMS

Mass spectrometry was performed on a Thermo Scientific LTQ FLEET mass spectrometer or a Finnigan LCQ mass spectrometer. HR-ESIMS were carried out on a Bruker ESI Quadrupole TOF mass spectrometer. MS^2 experiments were carried out on a Thermo Scientific LTQ FLEET mass spectrometer. Isotopic patterns were simulated using IsoPro, version 3.1.

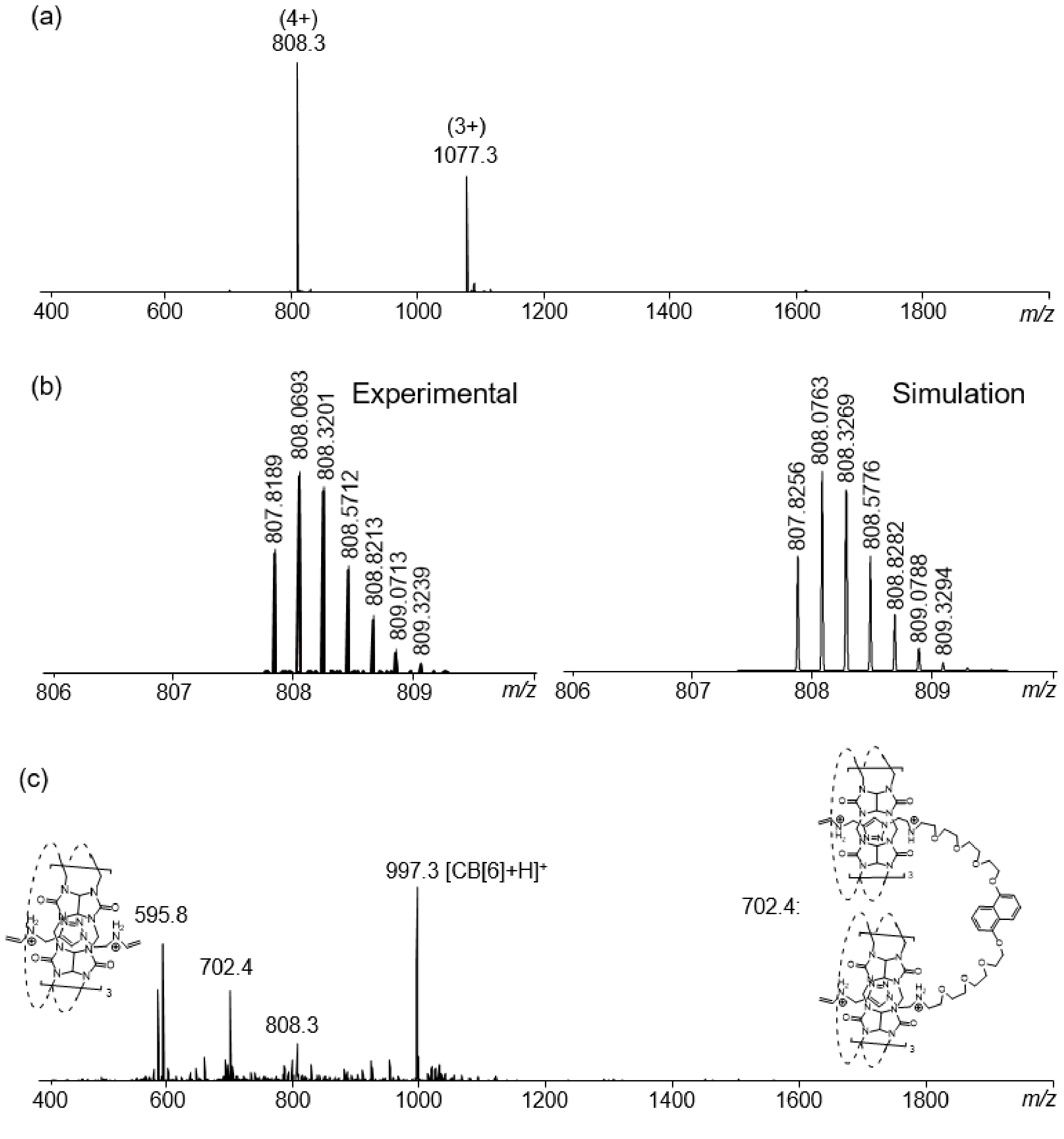

Figure S59. (a) ESI-MS spectrum of **Cat-1**, (b) HRMS of the peak at *m/z* = 808.3 (left: experimental; right: simulation) (c) MS^2 spectrum of **Cat-1** upon fragmentation of the peak at *m/z* = 808.3.

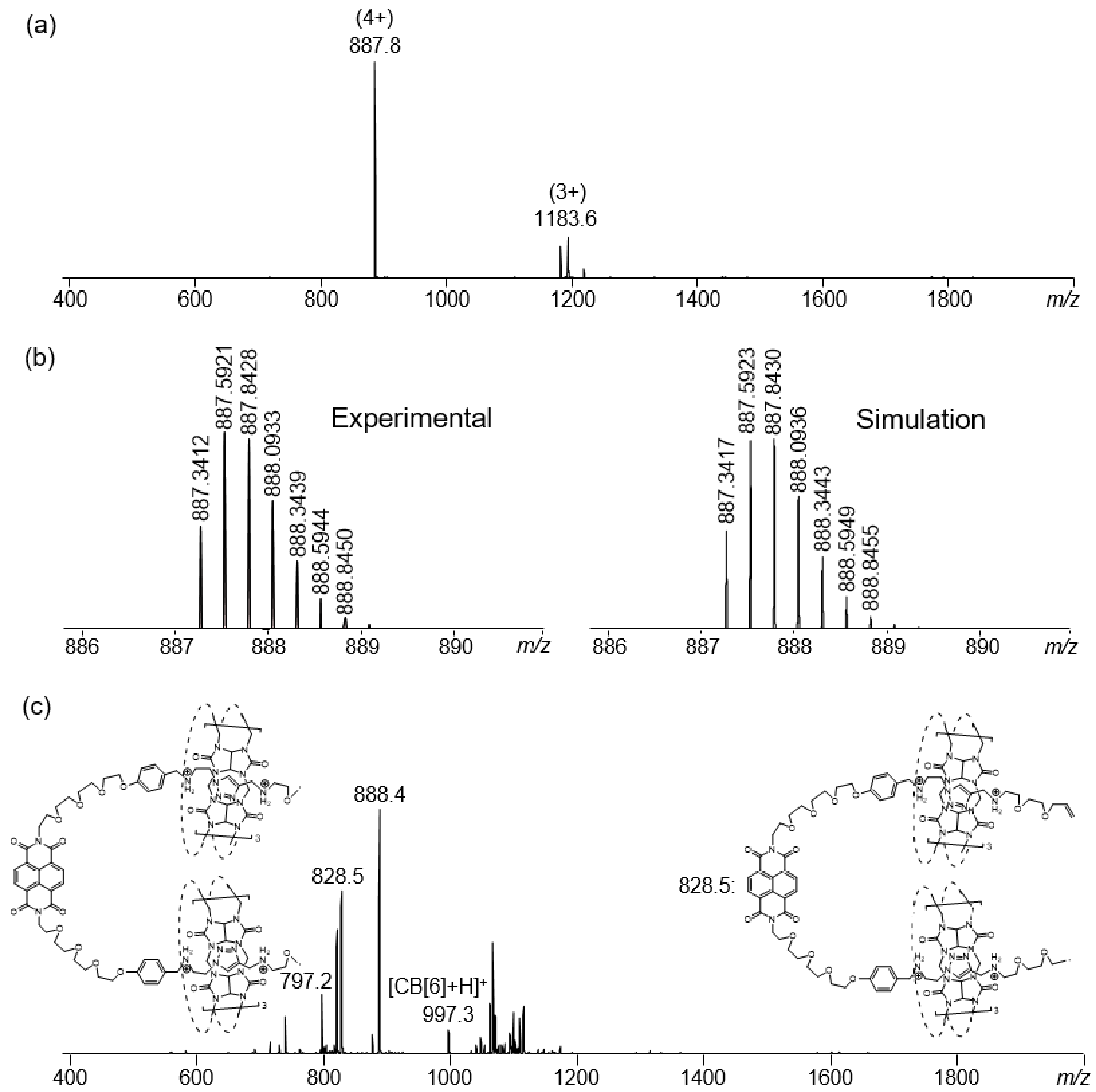

Figure S60. (a) ESI-MS spectrum of **Cat-2**, (b) HRMS of the peak at *m/z* = 887.8 (left: experimental; right: simulation) (c) MS^2 spectrum of **Cat-2** upon fragmentation of the peak at *m/z* = 887.8.

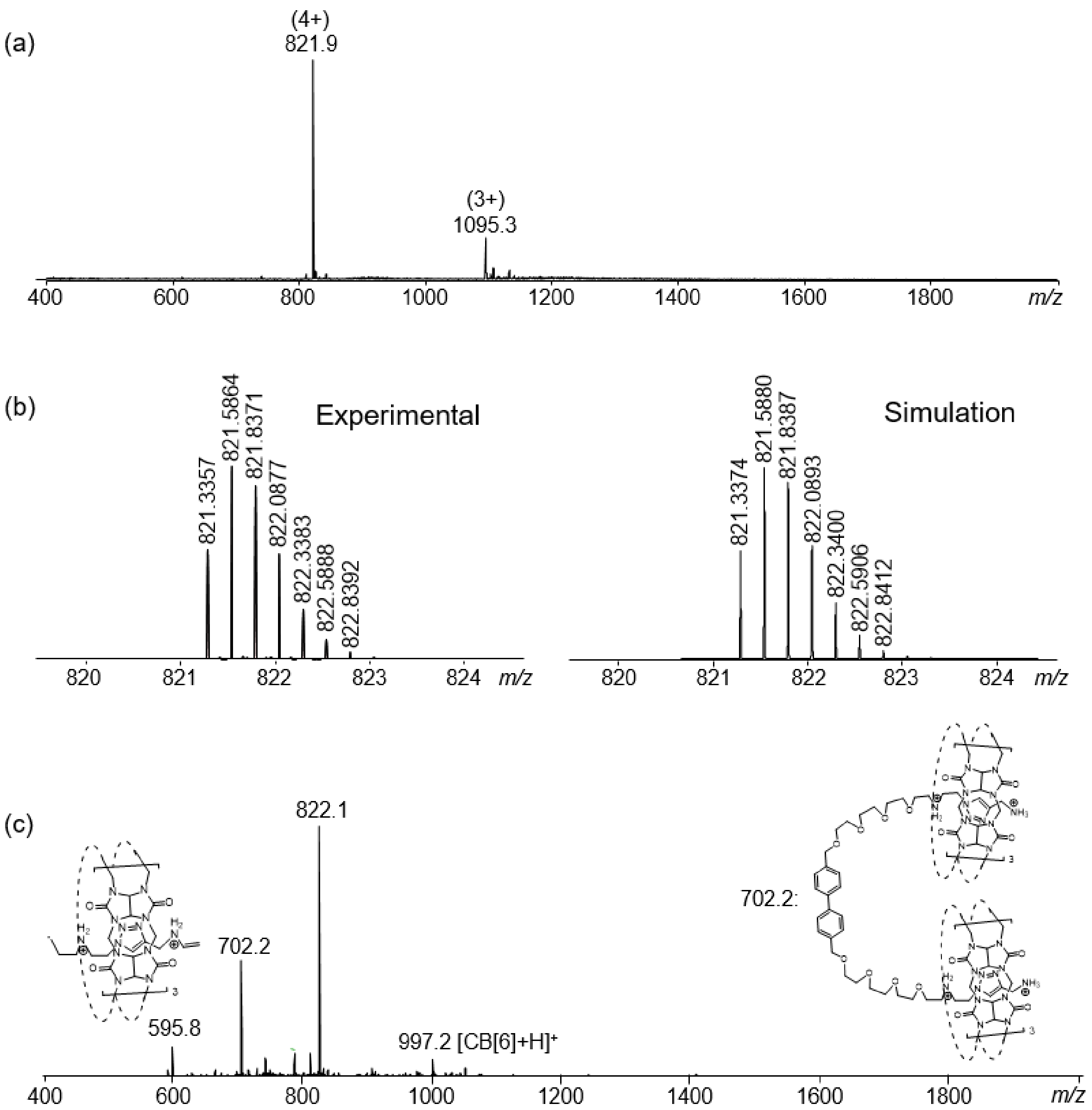

Figure S61. (a) ESI-MS spectrum of **Cat-3**, (b) HRMS of the peak at *m/z* = 821.9 (left: experimental; right: simulation) (c) MS^2 spectrum of **Cat-3** upon fragmentation of the peak at *m/z* = 821.9.

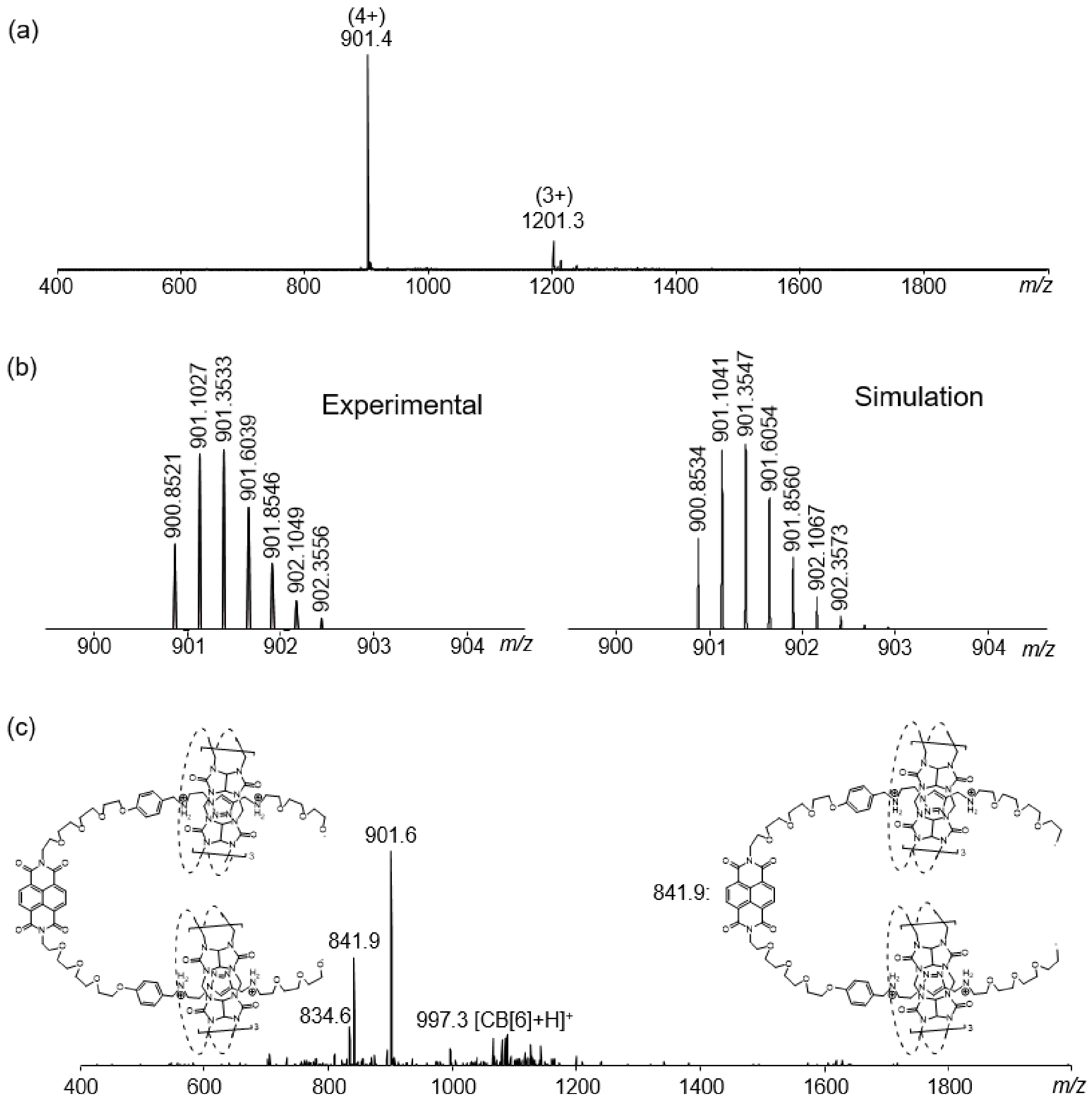

Figure S62. (a) ESI-MS spectrum of **Cat-4**, (b) HRMS of the peak at *m/z* = 901.4 (left: experimental; right: simulation) (c) MS^2 spectrum of **Cat-4** upon fragmentation of the peak at *m/z* = 901.4.

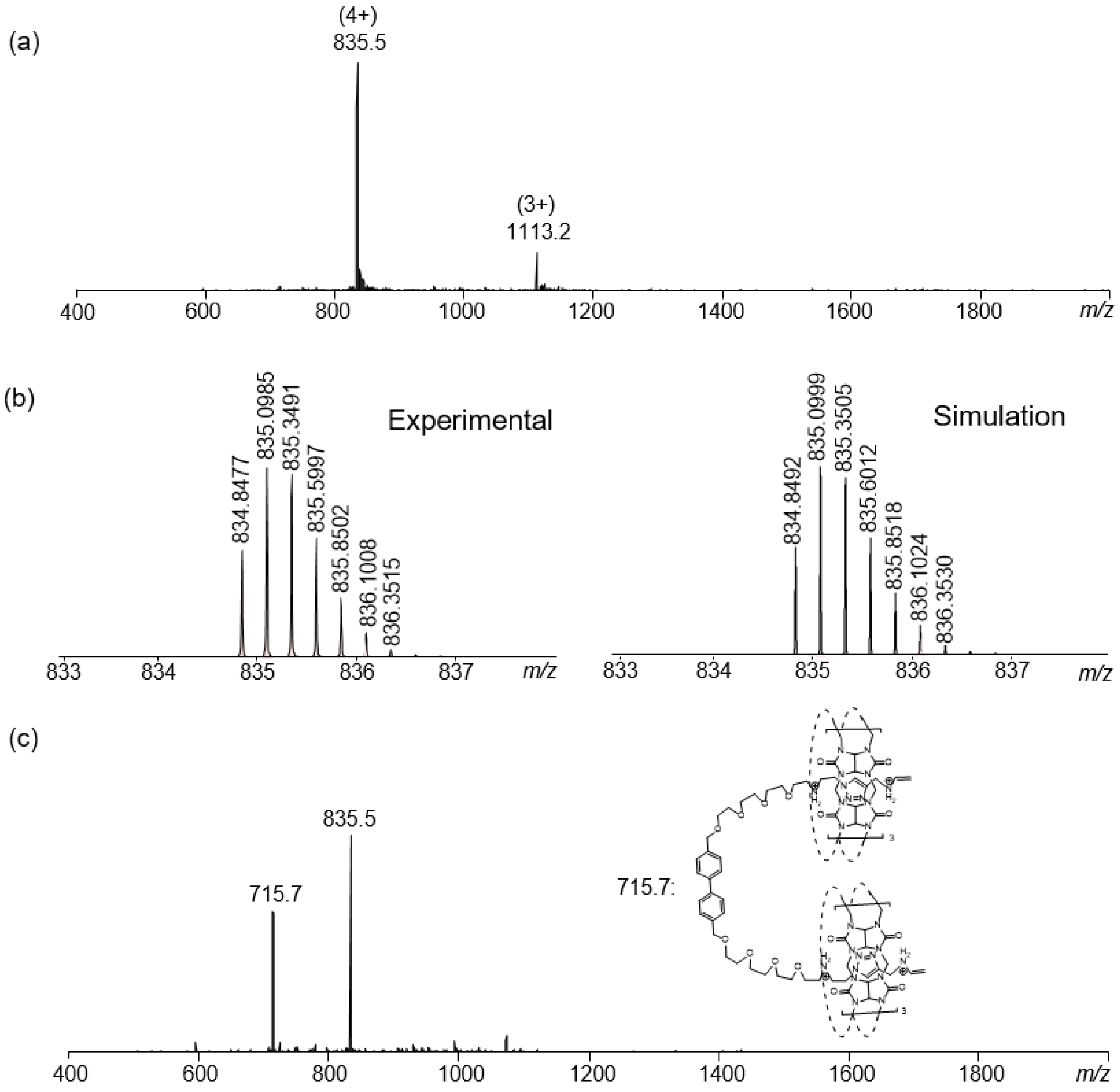

Figure S63. (a) ESI-MS spectrum of **Cat-5**, (b) HRMS of the peak at *m/z* = 835.5 (left: experimental; right: simulation) (c) MS^2 spectrum of **Cat-5** upon fragmentation of the peak at *m/z* = 835.5.

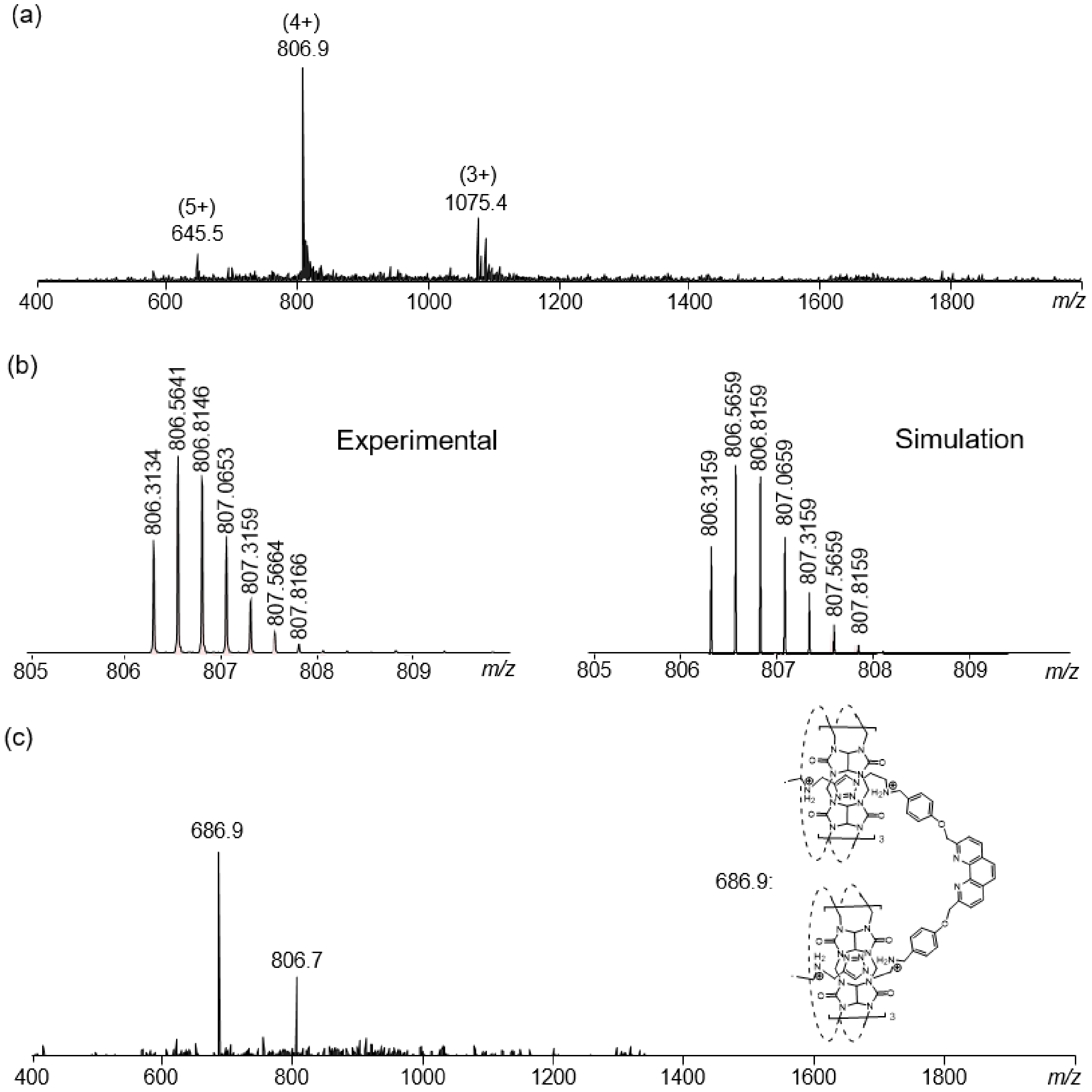

Figure S64. (a) ESI-MS spectrum of **Cat-6**, (b) HRMS of the peak at *m/z* = 806.9 (left: experimental; right: simulation) (c) MS^2 spectrum of **Cat-6** upon fragmentation of the peak at *m/z* = 806.9

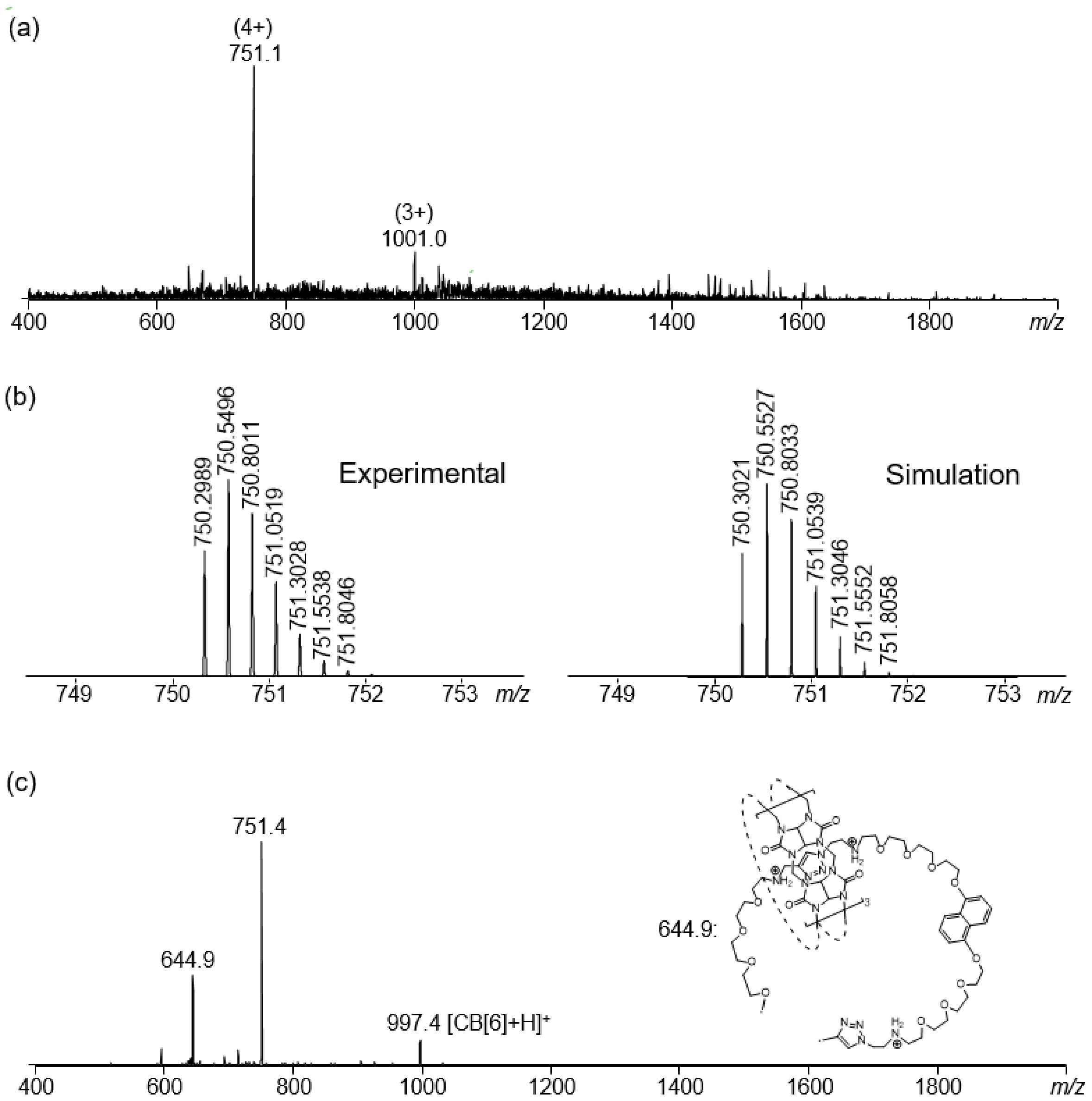

Figure S65. (a) ESI-MS spectrum of **Cat-7**, (b) HRMS of the peak at *m/z* = 751.1 (left: experimental; right: simulation) (c) MS^2 spectrum of **Cat-7** upon fragmentation of the peak at *m/z* = 751.1.

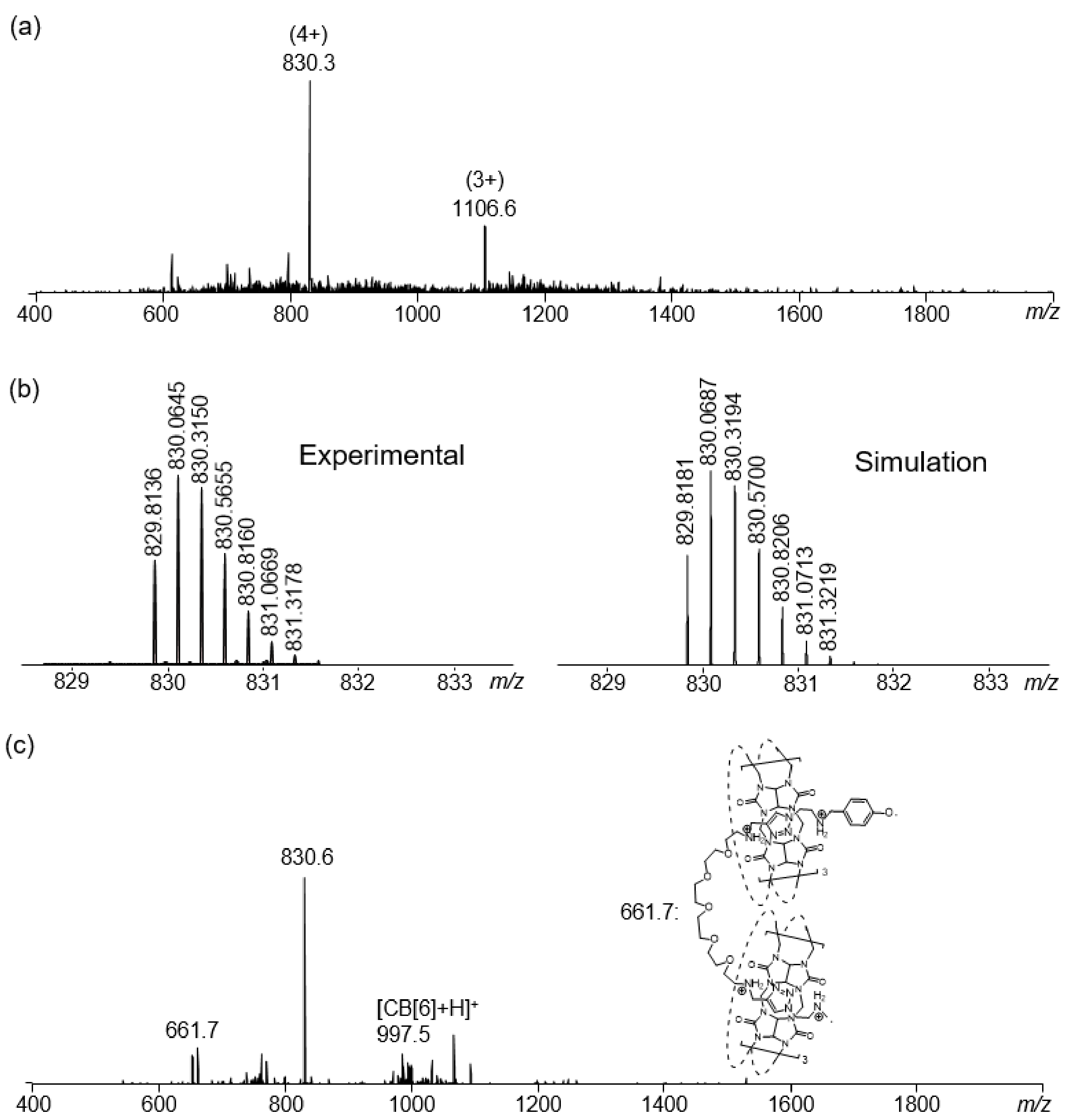

Figure S66. (a) ESI-MS spectrum of **Cat-8**, (b) HRMS of the peak at *m/z* = 830.3 (left: experimental; right: simulation) (c) MS^2 spectrum of **Cat-8** upon fragmentation of the peak at *m/z* = 830.3.

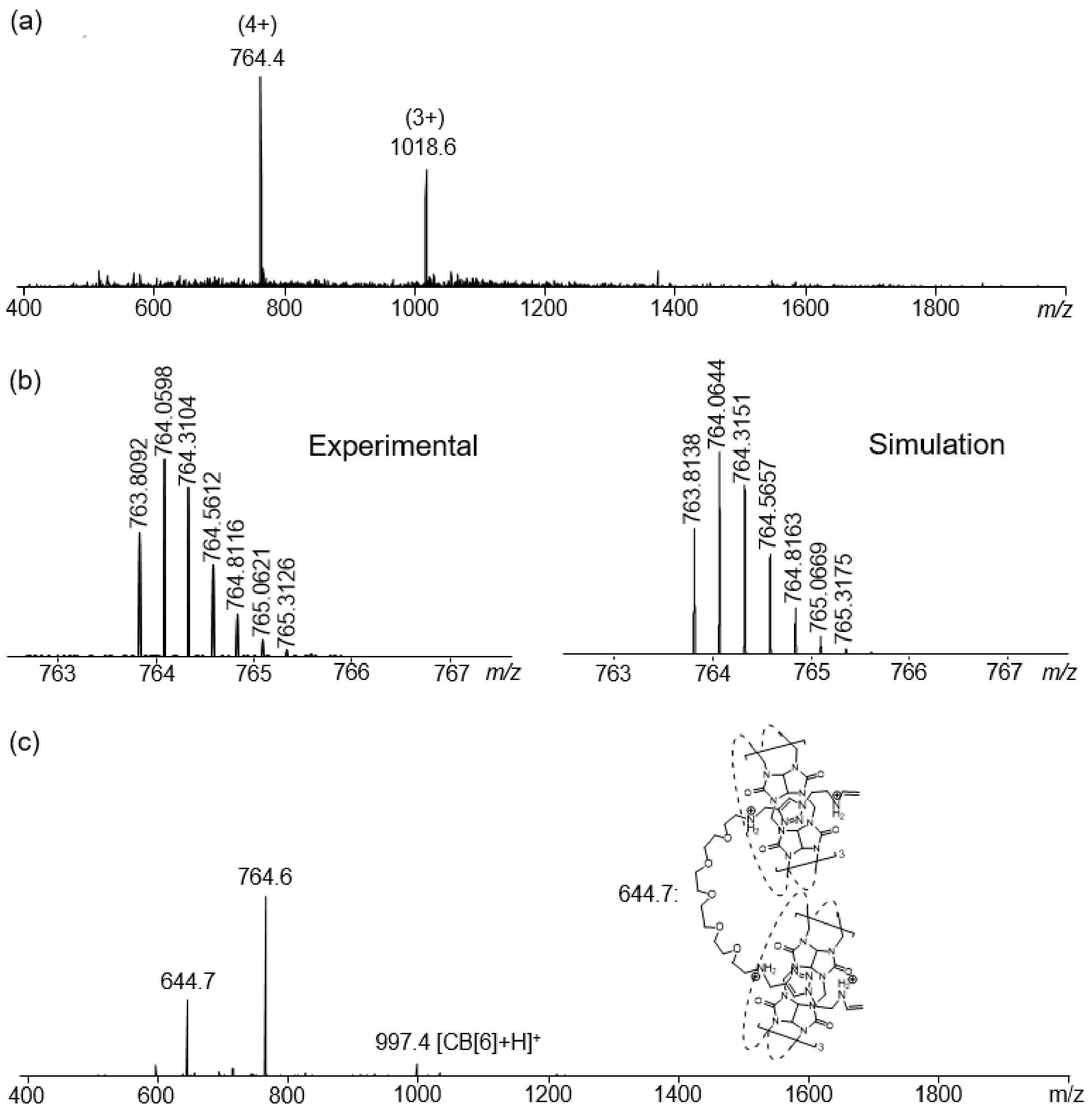

Figure S67. (a) ESI-MS spectrum of **Cat-9**, (b) HRMS of the peak at *m/z* = 764.4 (left: experimental; right: simulation) (c) MS^2 spectrum of **Cat-9** upon fragmentation of the peak at *m/z* = 764.4.

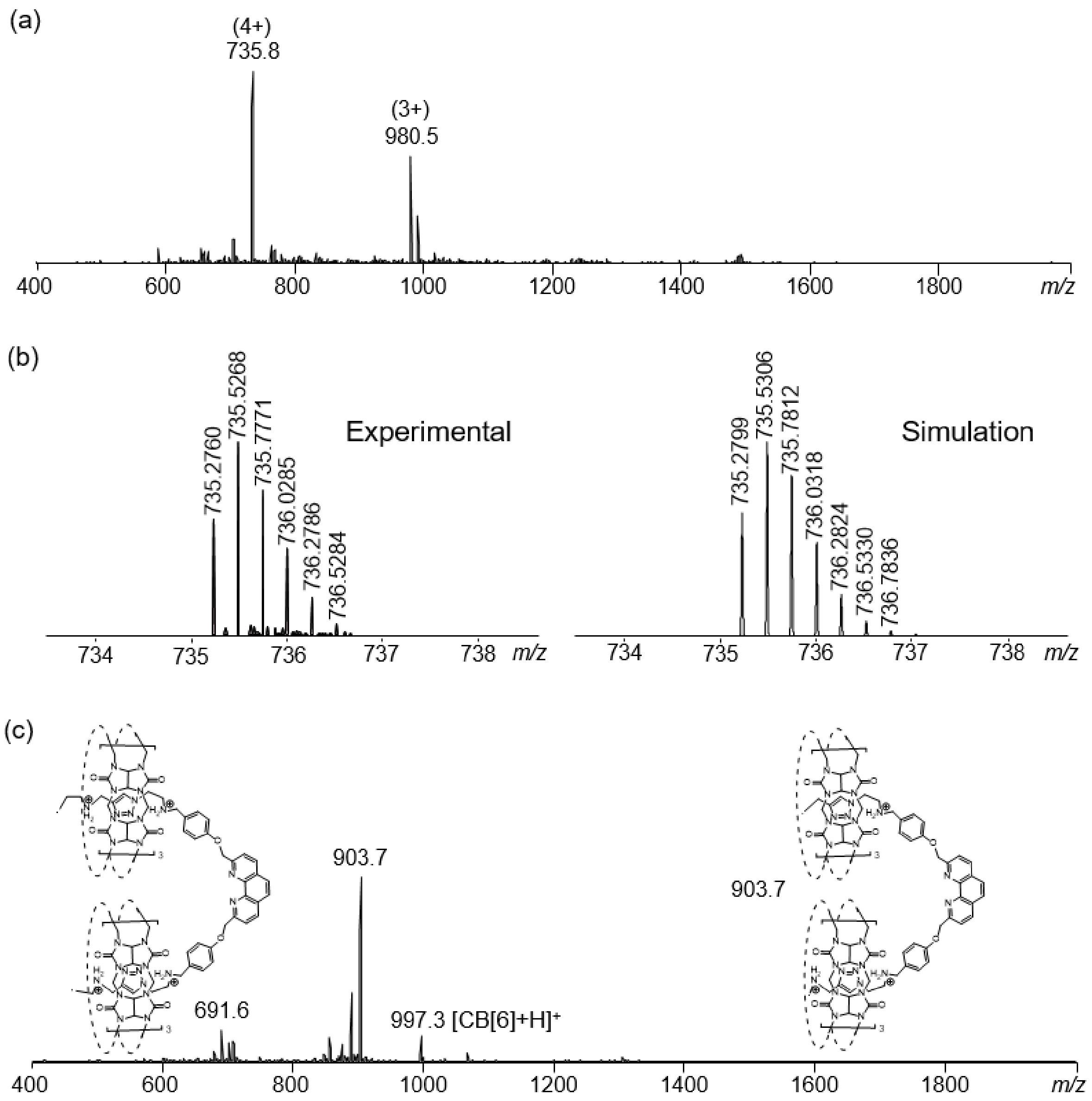

Figure S68. (a) ESI-MS spectrum of **Cat-10**, (b) HRMS of the peak at *m/z* = 735.8 (left: experimental; right: simulation) (c) MS^2 spectrum of **Cat-10** upon fragmentation of the peak at *m/z* = 735.8.

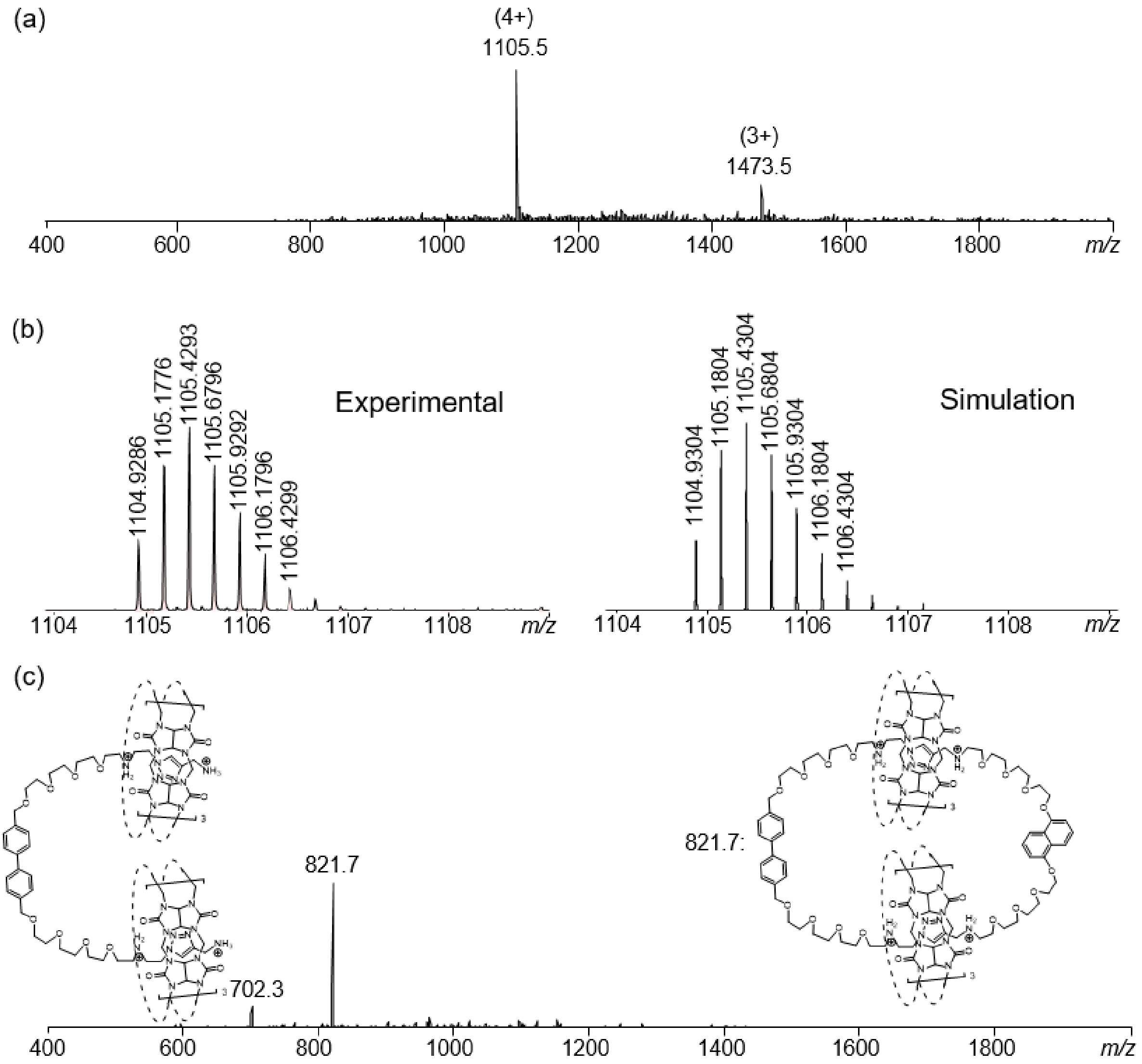

Figure S69. (a) ESI-MS spectrum of **Cat-11**, (b) HRMS of the peak at *m/z* = 1105.5 (left: experimental; right: simulation) (c) MS^2 spectrum of **Cat-11** upon fragmentation of the peak at *m/z* = 1105.5.

5. UV–vis

UV–vis spectra were recorded using an Agilent Cary 60 UV–vis spectrophotometer. The spectra were obtained in either water (for catenanes isolated as formate from preparative HPLC) or CH_3CN (for catenanes isolated as PF_6^- salts).

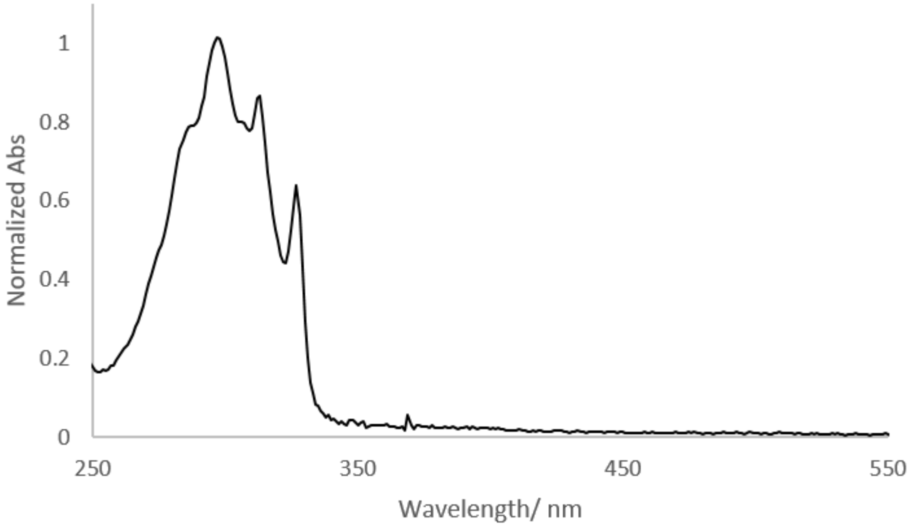

Figure S70. UV–vis spectrum of **Cat-1**.

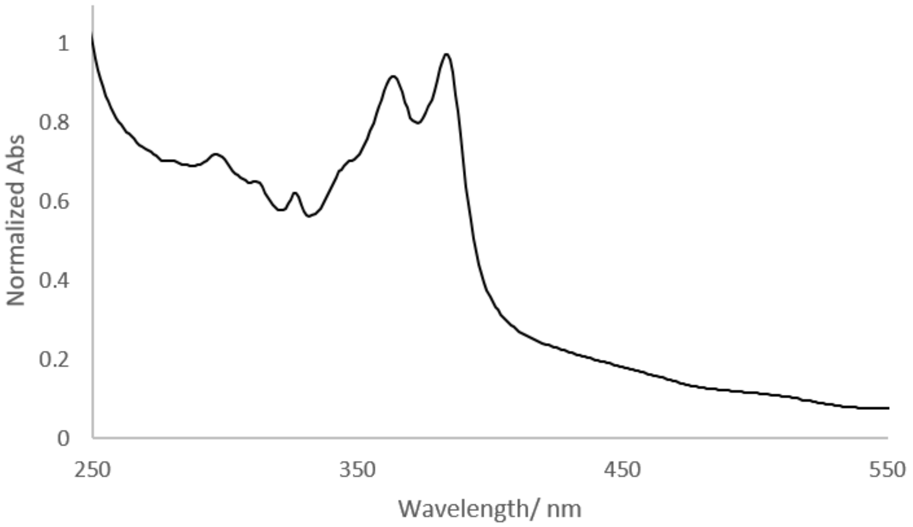

Figure S71. UV–vis spectrum of **Cat-2**.

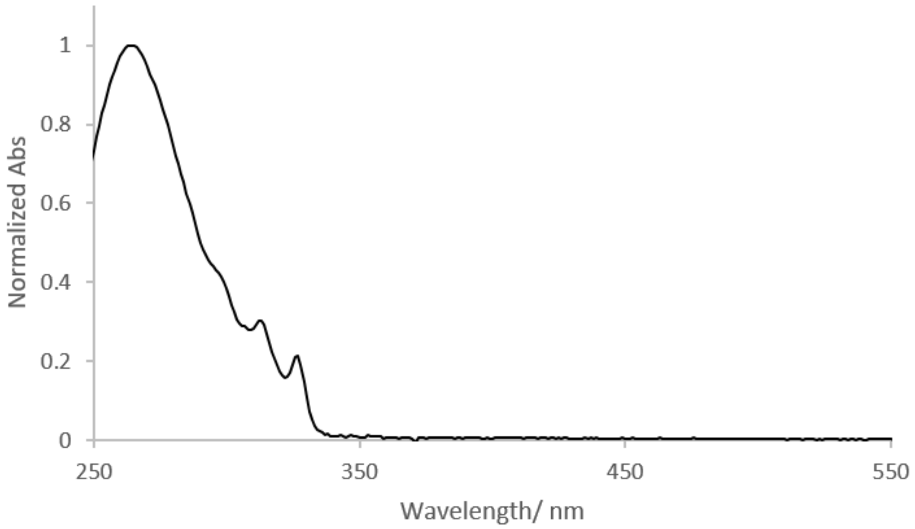

Figure S72. UV–vis spectrum of **Cat-3**.

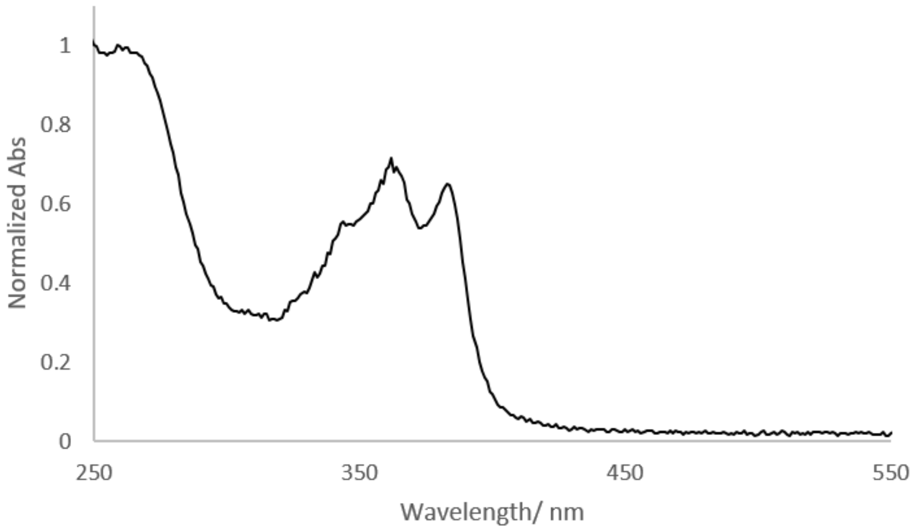

Figure S73. UV–vis spectrum of **Cat-4**.

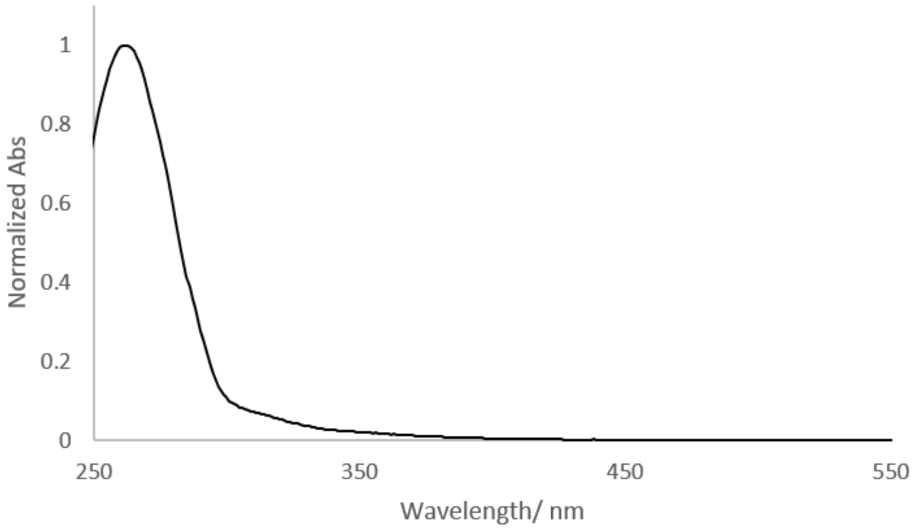

Figure S74. UV–vis spectrum of **Cat-5**.

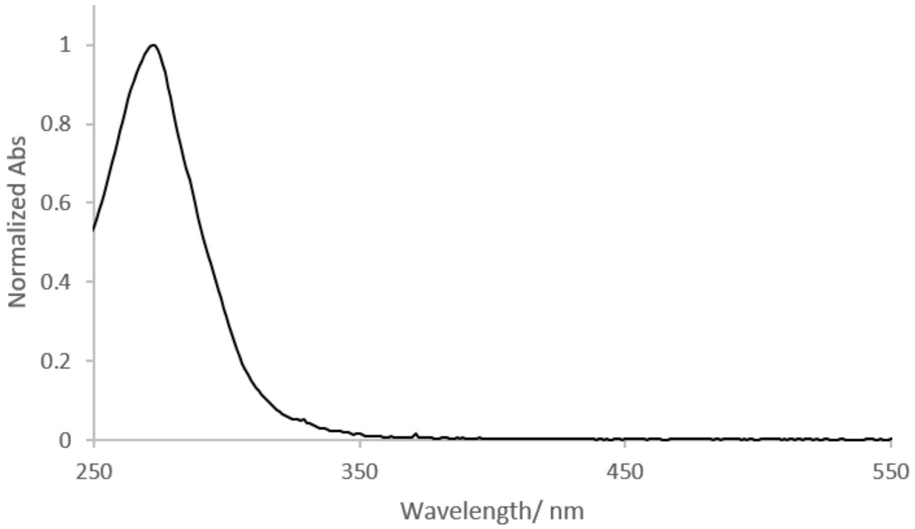

Figure S75. UV–vis spectrum of **Cat-6**.

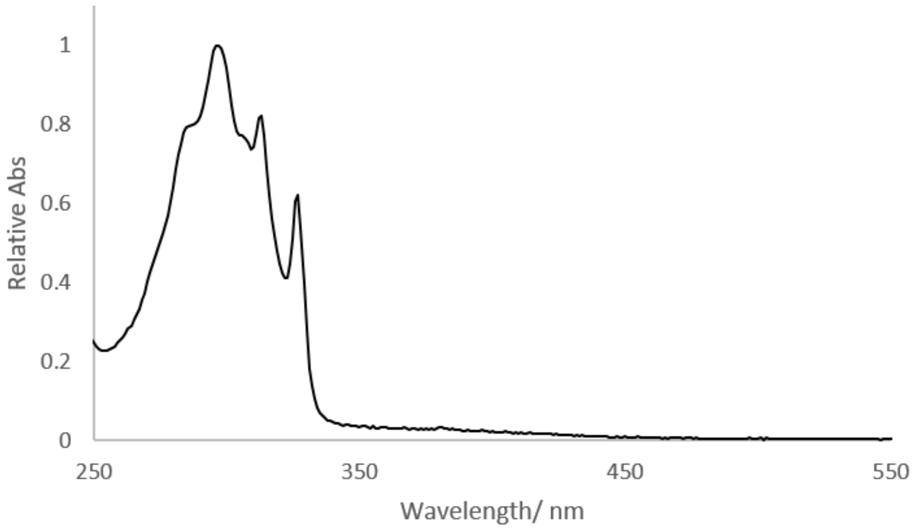

Figure S76. UV–vis spectrum of **Cat-7**.

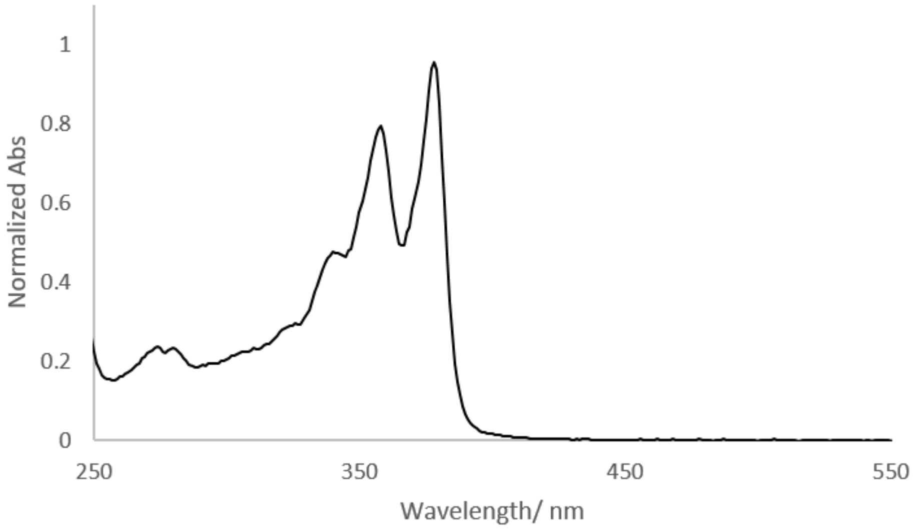

Figure S77. UV–vis spectrum of **Cat-8**.

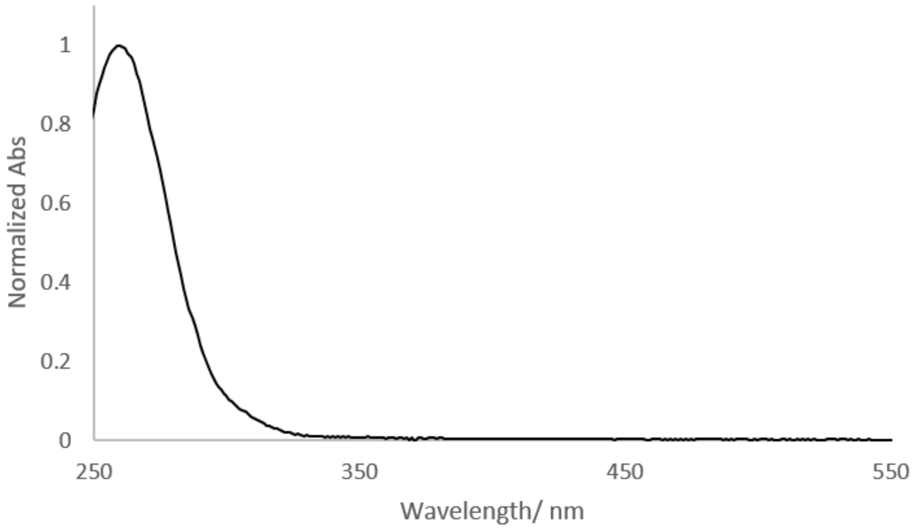

Figure S78. UV–vis spectrum of **Cat-9**.

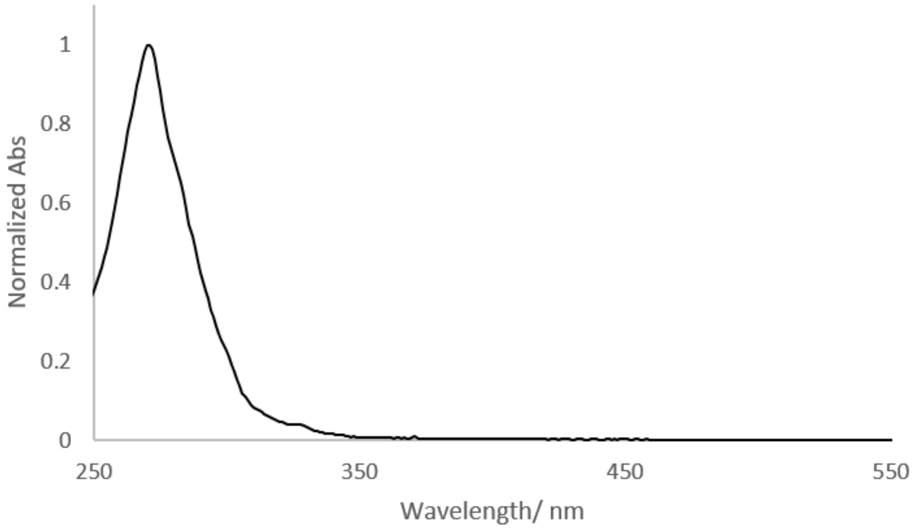

Figure S79. UV–vis spectrum of **Cat-10**.

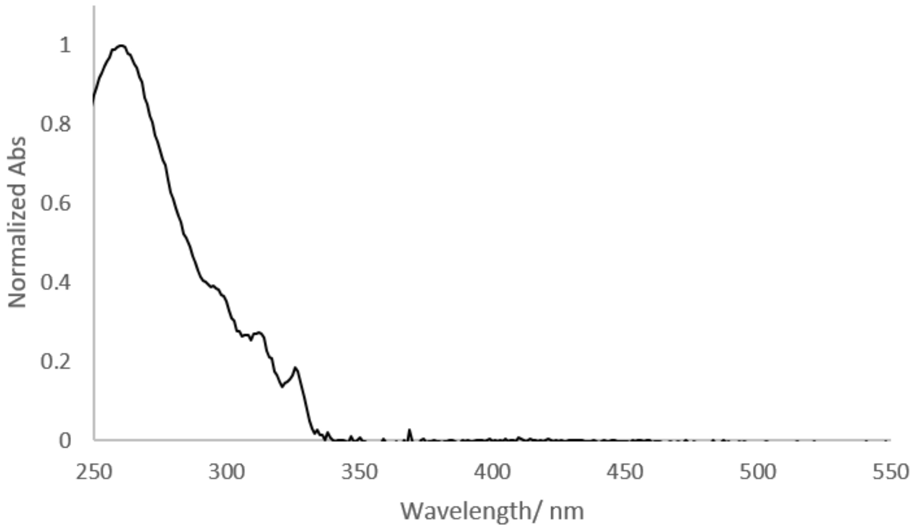

Figure S80. UV–vis spectrum of **Cat-11**.

6. References

1. Ashton, P. R.; Huff, J.; Menzer, S.; Parsons, I. W.; Preece, J. A.; Stoddart, J. F.; Tolley, M. S.; White A. J. P.; Williams, D. J. *Chem. Eur. J.,* **1996**, *2*, 31−44.
2. Basu, S.; Coskun, A.; Friedman, D. C.; Olson, M. A.; Benítez, D.; Tkatchouk, E.; Barin, G.; Yang, J.; Fahrenbach, A. C.; Goddard, W. A. III; Stoddart, J. F. *Chem. Eur. J.*, **2011**, *17*, 2107−2119.
3. Choudhary, U.; Northrop, B. H.; *Organic letters*, **2012**, *14*, 2082-2085.
4. Shan, M.; Carlson, K. E.; Bujotzek, A.; Wellner, A.; Gust, R.; Weber, M.; Katzenellenbogen, J. A.; Haag, R. *ACS chemical biology*, **2013**, *8*, 707-715.
5. Wang, K.; Yee, C. C.; Au-Yeung, H. Y. *Chem. Sci.*, **2016**, *7*, 2787-2792.
6. Angelos, S.; Yang, Y.-W.; Patel, K.; Stoddart J. F.; Zink, J. I. *Angew. Chem. Int. Ed.,* **2008***, 47,* 2222−2226.
